# Prognostic algorithms for post-discharge readmission and mortality among mother-infant dyads: an observational study protocol

**DOI:** 10.3389/fepid.2023.1233323

**Published:** 2023-11-29

**Authors:** Matthew O. Wiens, Jessica Trawin, Yashodani Pillay, Vuong Nguyen, Clare Komugisha, Nathan Kenya-Mugisha, Angella Namala, Lisa M. Bebell, J. Mark Ansermino, Niranjan Kissoon, Beth A. Payne, Marianne Vidler, Astrid Christoffersen-Deb, Pascal M. Lavoie, Joseph Ngonzi

**Affiliations:** ^1^ Institute for Global Health, BC Children’s and Women’s Hospitals, Vancouver, BC, Canada; ^2^ Department of Anesthesiology, Pharmacology & Therapeutics, University of British Columbia, Vancouver, BC, Canada; ^3^ WALIMU, Kampala, Uganda; ^4^ Department of Obstetrics & Gynaecology, Jinja Regional Referral Hospital, Jinja, Uganda; ^5^ Department of Medicine, Division of Infectious Diseases, Medical Practice Evaluation Center, Center for Global Health, Massachusetts General Hospital and Harvard Medical School, Boston, MA, United States; ^6^ Department of Pediatrics, University of British Columbia, Vancouver, BC, Canada; ^7^ Digital Health Research, BC Children’s Hospital Research Institute, Vancouver, BC, Canada; ^8^ School of Population and Public Health, Faculty of Medicine, University of British Columbia, Vancouver, BC, Canada; ^9^ Department of Obstetrics & Gynaecology, Faculty of Medicine, University of British Columbia, Vancouver, BC, Canada; ^10^ Department of Obstetrics and Gynaecology, Mbarara University of Science & Technology, Mbarara, Uganda

**Keywords:** sepsis, discharge, post-discharge, maternal health, neonatal health

## Abstract

**Introduction:**

In low-income country settings, the first six weeks after birth remain a critical period of vulnerability for both mother and newborn. Despite recommendations for routine follow-up after delivery and facility discharge, few mothers and newborns receive guideline recommended care during this period. Prediction modelling of post-delivery outcomes has the potential to improve outcomes for both mother and newborn by identifying high-risk dyads, improving risk communication, and informing a patient-centered approach to postnatal care interventions. This study aims to derive post-discharge risk prediction algorithms that identify mother-newborn dyads who are at risk of re-admission or death in the first six weeks after delivery at a health facility.

**Methods:**

This prospective observational study will enroll 7,000 mother-newborn dyads from two regional referral hospitals in southwestern and eastern Uganda. Women and adolescent girls aged 12 and above delivering singletons and twins at the study hospitals will be eligible to participate. Candidate predictor variables will be collected prospectively by research nurses. Outcomes will be captured six weeks following delivery through a follow-up phone call, or an in-person visit if not reachable by phone. Two separate sets of prediction models will be built, one set of models for newborn outcomes and one set for maternal outcomes. Derivation of models will be based on optimization of the area under the receiver operator curve (AUROC) and specificity using an elastic net regression modelling approach. Internal validation will be conducted using 10-fold cross-validation. Our focus will be on the development of parsimonious models (5–10 predictor variables) with high sensitivity (>80%). AUROC, sensitivity, and specificity will be reported for each model, along with positive and negative predictive values.

**Discussion:**

The current recommendations for routine postnatal care are largely absent of benefit to most mothers and newborns due to poor adherence. Data-driven improvements to postnatal care can facilitate a more patient-centered approach to such care. Increasing digitization of facility care across low-income settings can further facilitate the integration of prediction algorithms as decision support tools for routine care, leading to improved quality and efficiency. Such strategies are urgently required to improve newborn and maternal postnatal outcomes.

**Clinical trial registration:**

https://clinicaltrials.gov/, identifier (NCT05730387).

## 1. Introduction

Worldwide, approximately 290,000 maternal and 2.4 million neonate deaths occurred in 2020 (1, 2). Sub-Saharan Africa accounts for the largest burden of these deaths, which could be prevented through improved care coverage and quality (2, 3). While important global efforts are underway to improve outcomes immediately following birth, care following facility discharge remains a challenge. The postnatal period accounts for a high proportion of overall maternal and neonatal morbidity, representing a time of exceptional risk for mothers and their infants (4).

In facilities across sub-Saharan Africa, women and their newborn infants are routinely discharged from facility within 24 h of birth. Yet, 30% of mothers and 55% of newborns who die during the first 6 weeks do so more than 24 h after birth, with deaths often occurring within the community setting (5, 6). Despite this, studies focusing specifically on the post-discharge period following delivery, are rare (7, 8). Understanding the risk factors for maternal and newborns complications and death during the post-discharge period is an important first step to designing effective interventions.

Furthermore, maternal and neonate outcomes are inextricably connected. Recent evidence suggests maternal mortality and complications are associated with neonatal mortality and failure to thrive (9–11). Hence, considering the mother and child as a pair or dyad, offers an opportunity to potentially prevent interrelated causes of postpartum maternal and neonate complications (12). However, this approach has rarely been utilized in such studies.

The settings most affected by high rates of post-discharge morbidity and mortality suffer from overburdened and underinvested health systems and impoverished communities. Together, these greatly hinder adherence to the recommended frequency of postnatal visits resulting in many mothers and newborns not receiving timely lifesaving interventions (13). Currently, the World Health Organization (WHO) recommends three in-person postnatal visits for all mothers and newborn infants born at a health facility within the first six weeks of life (14). However, achieving sufficient postpartum follow-up care remains a major challenge in low-income settings, with a significant proportion of mothers and newborns not receiving even a single follow-up visit prior to the first 6-week newborn vaccination (13).

Given the persistent challenges in achieving high levels of post-partum care for mothers and newborns, as well as significant resource challenges within health facilities, there is an important opportunity to tailor recommendations for timing and frequency of follow-up to individual risk (15). This not only conserves the use of scarce resources but allows health workers to emphasize the importance of follow-up with individualized data on risk. Personalized risk scores can help frontline healthcare providers identify high-risk dyads before discharge from hospital, and guide recommendations for essential postnatal care.

The aim of this study, therefore, is to derive post-discharge risk prediction algorithms that identify high-risk dyads prior to hospital discharge, following facility birth. The ultimate goal of this work is to combine risk prediction algorithms with interventions that target prevention or treatment of critical causes of death or illness, inform evidence-based recommendations for postnatal care (PNC), and improve health outcomes for dyads.

## 2. Methods and analysis

### 2.1. Design and setting

This prospective observational study will enroll mother and newborn dyads from two regional referral hospitals in Uganda: The Mbarara Regional Referral Hospital in southwestern Uganda and Jinja Regional Referral Hospital in eastern Uganda. These two facilities serve a catchment covering 2 districts and a population of approximately 973,000 individuals. Together they provide a reasonable representation of the Ugandan population outside of the capital city of Kampala. Study results will be reported using the TRIPOD Checklist for Prediction Model Development and Validation (https://www.tripod-statement.org/) (Supplementary Data Sheet 1) and the STROBE Checklist for Cohort Studies (https://www.strobe-statement.org/) (Supplementary Data Sheet 2).

### 2.2. Study population

This study will be conducted between April 2022 and September 2023, and will enroll women and adolescent girls aged 12 and above delivering single or multiple babies. Only participants living in districts within the catchment area will be eligible for inclusion. Exclusion criteria includes those unable to speak the dominant local languages (Runyankole or Lusoga), refusal or inability to provide consent, residing in a refugee camp, as well as those with no access to a phone during the post-discharge period.

### 2.3. Sampling and randomization

A systematic sampling method for participant selection based on time of arrival will be used to reduce sampling bias. In order to capture a representative sample of all patients arriving within a 24-h period, the proportion of day and night admissions will be determined using hospital records. We will define daytime admissions as patients admitted for delivery between 8am and 8pm, and nighttime admissions as patients admitted for delivery between 8pm and 8am. Nighttime admissions will be assigned numbers based on order of arrival taken from hospital records and proportionately and randomly screened and selected using a random number generator application installed on an android tablet on the morning following their arrival. If the patient is not eligible for enrolment, nurses will continue using the random generator until the target for night enrolment is met. Daytime admissions will be screened and selected using time cut-offs whereby study nurses will be instructed to screen the first patient arriving after each 30-min interval. If the screened patient is not eligible, the next patient arriving after the time cut-off will be selected. These intervals will be staggered on shifts with multiple nurses to improve efficiency and length will be determined based on time spent with each patient during the screening, consenting and enrolment process. The daytime and nighttime assignments will be implemented for practical reasons to accommodate ward staffing, as well as to ensure budget efficiency. This process will allow us to capture a pseudo-random sample that was representative of admissions for delivery within a 24-h period, and not weighted towards either daytime or nighttime arrivals.

### 2.4. Consent

Research nurses will recruit and obtain consent from eligible women after they have been admitted to the facility for delivery and prior to discharge. Eligible women will be informed of the study and asked if they wish to participate. If the eligible participant is a minor, the parent or legal guardians(s) of the participant will be approached first by the research nurse. In accordance with the expectations of the Uganda National Council for Science and Technology Research Guidelines for Research involving Humans, consent will not be sought until after the patient has received an initial assessment by health workers at the time of admission for delivery, and research nurses may wait until after delivery to approach eligible women so as to not interfere with standard care. Women who suffer a loss or who are caring for a severely ill infant will be given the option to defer the interview portion of data collection until the six-week postnatal follow-up period. During the consenting process, all women will be told they are free to withdraw from the study at any point without needing to give a reason for withdrawal. Data collected up to the point of withdrawal will be de-identified and retained for analysis.

### 2.5. Data collection procedures

**Figure 1:**
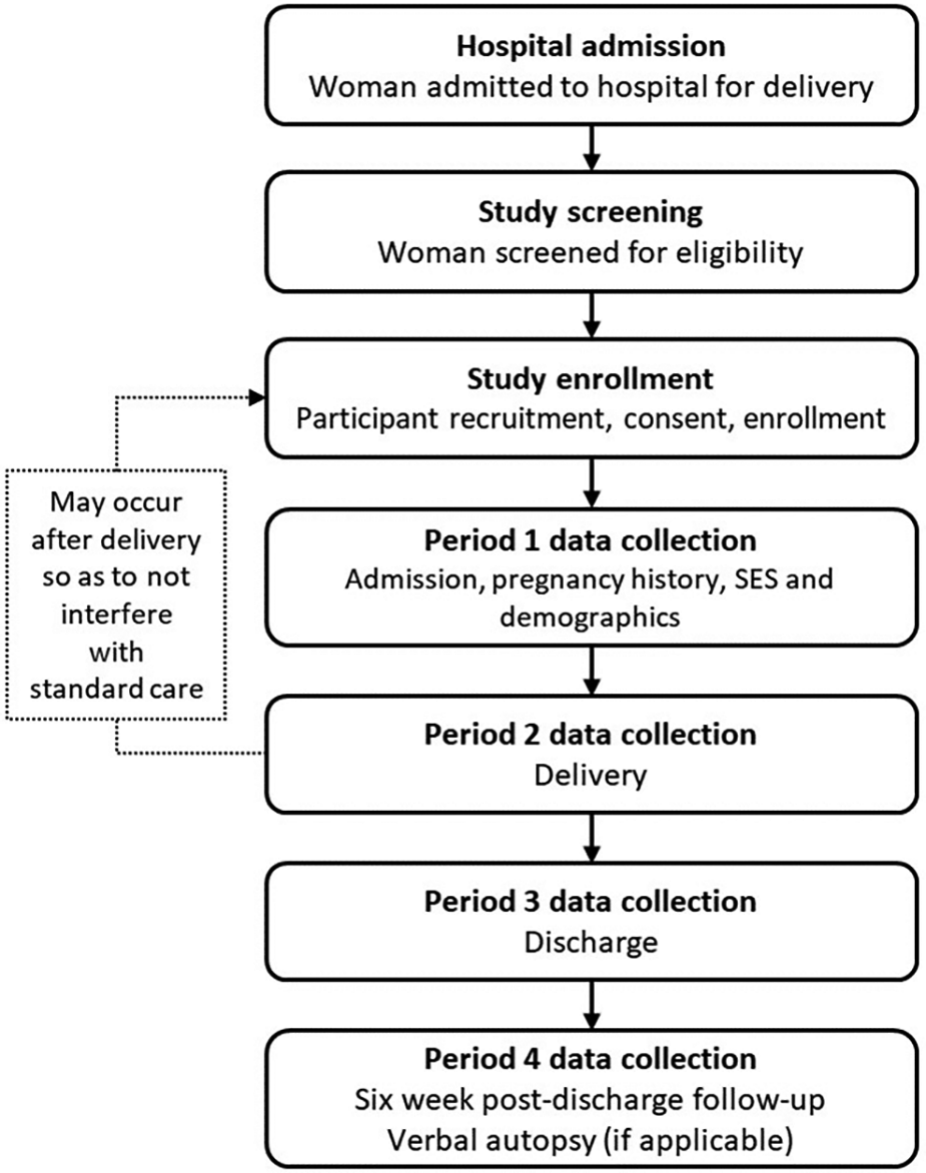
Study procedures.

Following informed consent of the mother, data collection will be represented by four data collection periods: Admission, delivery, discharge and 6-week post-discharge follow-up (Figure 1) (16). We will prioritize data collection from the hospital medical record, followed by interviews with the mother and finally confirmation with the medical team if there are discrepancies, missing information, or questions the mother is unable to answer. For the admission data collection period, the study team will collect data related to both pregnancy history (e.g., parity and co-morbidities), admission data (e.g., vital signs) and basic demographic details (e.g., age and socio-economic details) (Supplementary Data Sheet 3). The delivery phase will represent the delivery details of both the mother (e.g., delivery mode) and the newborn (e.g., resuscitation and birth weight). At discharge, we will obtain and record clinical data for mothers and their newborns discharged alive from the hospital. Blood pressure will be measured using a Microlife Cradle VSA semi-automated blood pressure monitor (Microlife Corporation, Widnau, Switzerland). Oxygen saturation (SpO_2_) and heart rate will be collected using the Masimo iSpO_2_® (Masimo Corporation, California, USA) and Nellcor Oxicable (Medtronic, Minnesota, USA), and respiratory rates will be measured using the RRate Application (17, 18). Maternal hematocrit will be determined using a microhematocrit centrifuge. Anthropometric data of infants (length and weight) will also be measured and recorded by study nurses. All dyads will receive routine care during admission and are discharged at the discretion of their medical teams. In cases where study nurses note danger signs while collecting discharge measurements referrals to the pediatric unit will be made and recorded.

Six weeks following discharge, all mothers will be contacted by phone for the follow-up data collection phase which will capture outcomes, including health seeking, re-admissions and deaths. Verbal autopsies will be conducted in all cases of maternal or newborn death at least one month after a death has occurred. In the event that the mother has died, verbal autopsy data will be collected from the study participant's spouse or other family member. If study participants cannot be reached by phone they will be followed up in person by field officers in order to reduce loss to follow-up.

### 2.6. Identification of candidate predictor variables

Candidate predictor variables were selected based on results from a pilot study conducted at Mbarara Regional Referral Hospital (2019–2020) (15) and updated using literature and consultation with experts on the study team to ensure variables were likely to be predictive and available in low-resourced settings.

### 2.7. Outcomes

We will build two separate sets of prediction models to predict re-admission or death within six weeks of hospital discharge. The primary outcome for the first set of models will be newborn death or readmission while the primary outcome for the second will be maternal death or readmission. Secondary outcomes will include a list of risk factors associated with death and readmission among mothers and newborns, the proportion of mothers and newborns receiving routine postnatal care as well as predictors of postnatal care visits.

### 2.8. Sample size and statistical analysis plan

With a sample size of 6,700 and expected outcome rates of 2.7% for mothers and 4.4% for newborns, we will be able to measure the prevalence of maternal and newborn hospital readmission or death within a margin of error of 1%. We will be able to develop prediction models with up to 30 candidate predictors for both maternal and newborn outcomes separately, ensuring a mean absolute prediction error of no more than 1%, a uniform shrinkage factor of 90%, a Cox-Snell *R*^2^ value of at least 0.10, a maximum *R*^2^ of 0.05 and expected optimism of 0.05. The *R*^2^ value and number of predictor variables are based on findings from our epidemiological study.

We will summarise all risk factors for mothers and newborns that do and do not experience poor outcomes and estimate univariate associations. For newborns, data will be reported by sex. Derivation of prediction models will be based on optimization of the AUROC and specificity using elastic net regression. Internal validation will be assessed using 10-fold cross validation (19, 20). As in our other cohorts, we will focus on developing parsimonious predictive models (e.g., 5–10 predictor variables) with high sensitivity (>80%). This reduces false negatives and maximizes its use in resource-limited settings where frontline health workers are the main providers of postnatal care. AUROC, sensitivity, and specificity will be reported for each model, along with positive and negative predictive values. Site specific metrics will be compared to ensure consistency across settings, and recalibration may be considered if individual site performance is lower than expected. Finally, we will assess combined sensitivity and specificity when each individual model is applied to the dyad. Outside of prediction modelling, our sample size will allow us to detect an odds ratio of at least 1.30 for a given risk factor with 80% power and 5% significance and relative precision of 25%.

### 2.9. Data management

Study data will be managed using customized Research Electronic Data Capture (REDCap) tools hosted at the University of British Columbia, with secure access by all investigators in Uganda and Canada. REDCap is a secure, web-based application designed to support data capture for research studies. Data will be entered into the REDCap database by a trained research nurse or research assistant after each activity is completed and audio recordings are transcribed.

After the study period, a de-identified copy of the data will be prepared for deposition in a repository with open access with proper governance mechanisms. We will make every effort to prevent re-identification of subjects by coding data that has the potential of being identifiable.

## 3. Discussion

Using a prospective cohort of women delivering at two regional referral hospitals in Uganda, this study aims to derive risk prediction models for post-discharge mortality among mother-newborn dyads. A large proportion of post-discharge deaths occurring during the postnatal period are treatable and preventable through early recognition and high-quality care, fostering an emphasis on facility births and ongoing postnatal care as an early intervention (6, 21, 22). Despite the three recommended postnatal care visits in the first six weeks after delivery (14), only 7%–10% of postpartum women and approximately one third of newborns attend at least one postnatal care visit in Uganda (23–25). New approaches to postnatal care are therefore urgently required to improve maternal and infant outcomes during the postnatal period.

Precision health is a strategy that focuses on individualized assessment to optimize care provision at the patient level. Our previous work in pediatric sepsis has shown that linking the risk of post-discharge mortality to the intensity of discharge and post-discharge care has the potential to improve outcomes and also increase care compliance during the post-discharge period (26–28). By enabling effective allocation of scarce resources, such approaches are more likely to be sustainable in economically strained countries.

Building a tool to assess risk must consider the context in which it is to be implemented. In resource limited settings, a key consideration is that the variables used to assess risk (i.e., the candidate predictor variables) should be simple, objective and easily collected during routine care. Second, these tools must be functionally capable of integrating into routine care processes. As health systems in low-resourced settings increasingly adopt digital health systems, the integration of algorithms to augment clinical decisions (such as follow-up intensity) is becoming increasingly feasible (29, 30). As such, the traditional paper-based approaches to risk classification will be replaced by data-driven approaches such as algorithmic scoring. Our analysis plan and algorithmic output is especially conducive to such an approach. Through EMR integration, risk-prediction algorithms tied to effective care pathways have potential to both improve care and resource efficiency, both being critical in settings with low resources and high disease burden.

Individualized risk assessment in the absence of effective interventions is of limited utility. Likewise, an effective intervention in the absence of adherence is also of limited utility. The current recommendations for routine postnatal care, even if effective, are largely absent of benefit to the vast majority of mothers and newborns due to poor adherence. Improved timing of these visits, alongside a more personalized intensity of these visits, may both improve outcomes and adherence. Indeed, risk communication itself may influence outcomes, especially for those at risk who may not accurately perceive their own risk (31, 32). There is currently no way to accurately prognosticate risk among a general population of mothers and newborns. There is a dearth of literature in low-income countries examining overlapping outcomes and care seeking between mothers and infants during the postnatal period which we hope to address (33, 34). Using the data generated from this study, we aim to develop, plan and implement patient centered interventions to reduce mortality and readmission in the postnatal period in low-resourced settings.

### 3.1. Study limitations

This study is subject to several potential limitations. First, loss to follow-up may reduce our effective sample size, thus diminishing our power to detect significance in some variables. The exclusion criteria of no access to a mobile phone will improve follow-up potential, though this does limit generalizability to those with no phone access, who themselves may be more vulnerable. Our prior work, however, suggests that while many women do not own their own phone, fewer than 1% do not have access to a phone. Our prior work doing similar studies also suggests that our loss to follow-up will be less than 5%. Furthermore, our study sample size has compensated for potential loss to follow-up, thus we do not anticipate this to be an issue. Second, heterogeneity in outcomes may impact modelling of post-discharge events. We observed this to be a potential limitation in our prior work as predictors were different between newborns and mothers, and between mothers with caesarean sections and those who delivered vaginally. Therefore, we calculated our sample size estimates to be able to model at least 3 separate outcomes. We anticipate that this will be sufficient to create a set of models which are comprehensive to capture various outcomes. Finally, this study is limited to a single country, which limits its generalizability beyond Uganda. However, it is our intent to expand and validate in other countries if model building is successful in this study.

## Supplementary material

Supplementary Data Sheet 1 TRIPOD Checklist

Supplementary Data Sheet 2 STROBE Statement Checklist

Supplementary Data Sheet 3 Data Dictionary
