# Supplementary material for: Prognostic algorithms for post-discharge readmission and mortality among mother-infant dyads: an observational study protocol
Source: Front Epidemiol. 2023 Nov 29;3:1233323. doi: 10.3389/fepid.2023.1233323 (PMC10911031; doi:10.3389/fepid.2023.1233323)
Supplement: Supplementary Data Sheet 3 — Data Dictionary [file Datasheet3.pdf]

Appendix I: Smart Discharges for Mom & Baby
Data Dictionary Codebook

	#	Variable / Field Name	Field Label<br>***Field Note***	Field Attributes (Field Type, Validation, Choices, Calculations, etc.)
Instrument: **1. Admission Subject Details** (admission_subject_details)				
	24	[admitdate_adm_v2]	Date of admission	text (date_dmy)
	25	[admittime_adm_v2]	Time of admission (USE 24 HOUR TIME - HH:MM)	text (time)
	36	[dobknown_adm_v2]	Is exact date of birth known?	yesno
	37	[dob_adm_v2]<br>Show the field ONLY if:<br>[dobknown_adm_v2] = '1'	Date of birth	
	38	[dob_estage_adm_v2]<br>Show the field ONLY if:<br>[dobknown_adm_v2] = '0'	What is her estimated age in years?	
		Questions: 1-23 and 38-41 capture screening criteria and participant contact details		

Instrument: **2. Admission** (admission)

	41	[time_tohosp_adm]	Section Header: *Admission Details*<br>How long did it take you to travel to this hospital?	dropdown<br>1 less than 30 minutes<br>2 30 minutes - 1 hr<br>3 more than 1hrs and up to 2hrs<br>4 more than 2hrs and less than 4hrs<br>5 4 hours or more
	42	[transport_adm]	Primary mode of transportation to the hospital?	dropdown<br>1 Walk<br>2 Motorcycle<br>3 Public transport (bus, taxi)<br>4 Private transport (special hire, private vehicle)<br>5 Ambulance<br>98 Other
	43	[delay_adm]	Were you delayed >1 hour by any of the following (choose all that apply):	checkbox<br>1 delay_adm___1 Terrain (swamp, flood, water transport delay, etc.)<br>2 delay_adm___2 Cost of transport<br>3 delay_adm___3 General transport delay (waiting for the bus, schedules, travel at night, etc.)<br>4 delay_adm___4 Significant rain/threat of rain<br>98 delay_adm___98 Other<br>99 delay_adm___99 None
	44	[isreferral_adm]	Is this visit a referral?	yesno<br>1 Yes<br>0 No
	45	[referralsrc_adm]<br>Show the field ONLY if:<br>[isreferral_adm] = '1'	Referral source	dropdown<br>1 Other Hospital<br>2 Health Centre/Clinic<br>3 VHT<br>4 Untrained Health Worker<br>5 Traditional Healer<br>6 Traditional Birth Attendant<br>98 Other
	46	[csection_adm]	Section Header: *Admission clinical variables*<br>Is the mother here for elective c-section?	yesno<br>1 Yes<br>0 No
	47	[labordate_adm]<br>Show the field ONLY if:<br>[csection_adm] != '1'	Date labor started:	text (date_dmy)
	48	[labortime_adm]<br>Show the field ONLY if:<br>[csection_adm] != '1'	Time labor started:	text (time)
	49	[takevitals_adm]	Record BP, Temperature, and HR?	yesno<br>1 Yes<br>0 No

	50	[sys_bp_adm]<br><br>Show the field ONLY if:<br>[takevitals_adm] = '1'	Systolic blood pressure at admission	text (integer)
	51	[dia_bp_adm]<br><br>Show the field ONLY if:<br>[takevitals_adm] = '1'	Diastolic blood pressure at admission	text (integer)
	52	[temp_adm]<br><br>Show the field ONLY if:<br>[takevitals_adm] = '1'	Temperature at admission	text (number_1dp, Min: 35, Max: 42)
	53	[hr_adm]<br><br>Show the field ONLY if:<br>[takevitals_adm] = '1'	Heart rate at admission	text (integer)
	54	[distress_adm]	Was the woman in distress upon arrival?	dropdown<br>1 Yes<br>2 No<br>3 Unsure
	55	[admission_complete]	Section Header: *Form Status*<br>Complete?	dropdown<br>0 Incomplete<br>1 Unverified<br>2 Complete

Instrument: **3. Pregnancy History** (pregnancy_history)

	56	[medhx_adm]	Has the women been diagnosed with ANY of the following conditions BEFORE pregnancy (choose all that apply)	checkbox<br>1 medhx_adm___1 HIV (diagnosed BEFORE pregnancy)<br>2 medhx_adm___2 High blood pressure (diagnosed BEFORE pregnancy)<br>3 medhx_adm___3 Prior infertility (>1yr attempting to get pregnant but unsuccesful)<br>4 medhx_adm___4 Diabetes (diagnosed BEFORE pregnancy)<br>5 medhx_adm___5 Kidney disease<br>6 medhx_adm___6 Sickle cell<br>7 medhx_adm___7 Hepatitis B/C<br>8 medhx_adm___8 Tuberculosis<br>9 medhx_adm___9 Chronic mental illness<br>98 medhx_adm___98 Other diagnoses not listed here<br>99 medhx_adm___99 No prior diagnoses
	57	[duedate_adm]	Does the woman know her approximate due date?	yesno<br>1 Yes<br>0 No
	58	[duedate3_adm]<br><br>Show the field ONLY if:<br>[duedate_adm] = '1'	Due date (if known)	text (date_dmy)
	59	[duedate2_adm]<br><br>Show the field ONLY if:<br>[duedate_adm] = '1'	How was the due date defined?	dropdown<br>1 LNMP<br>2 Ultrasound<br>3 Woman herself
	60	[gravid_adm]	Section Header: *Pregnancy History*<br>How many pregnancies has she had in her lifetime (including this one)?	text (integer, Min: 1, Max: 20)

	61	[parity_adm]	How many pregnancies has she had with a baby born greater than 500g at birth delivered after 20 weeks gestation?	text (integer, Min: 0, Max: 20)
	62	[csect_adm]	Has woman had a prior c-section?	yesno<br>1 Yes<br>0 No
	63	[csect2_adm]<br>Show the field ONLY if:<br>[csect_adm] = '1'	How long ago was most recent c-section?	dropdown<br>1 < 18 months ago<br>2 18-36 months ago<br>3 >36 months ago
	64	[preghx_adm]	Has the woman been diagnosed with any of the following conditions DURING THIS pregnancy? (select all that apply)	checkbox<br>1 preghx_adm___1 Gestational diabetes<br>2 preghx_adm___2 Pre-eclampsia<br>3 preghx_adm___3 Eclampsia<br>4 preghx_adm___4 Gestational hypertension<br>5 preghx_adm___5 Antepartum hemorrhage/ vaginal bleeding (1st trimester)<br>6 preghx_adm___6 Antepartum hemorrhage/ vaginal bleeding (2nd trimester)<br>7 preghx_adm___7 Antepartum hemorrhage/ vaginal bleeding (3rd trimester)<br>8 preghx_adm___8 PPROM<br>9 preghx_adm___9 Preterm labour<br>10 preghx_adm___10 Malaria<br>11 preghx_adm___11 HIV (diagnosed DURING)<br>12 preghx_adm___12 Urinary tract infection<br>13 preghx_adm___13 Tuberculosis<br>14 preghx_adm___14 Anemia<br>15 preghx_adm___15 COVID-19 (suspected)<br>16 preghx_adm___16 COVID-19 (posititive test)<br>17 preghx_adm___17 Hepatitis B/C<br>18 preghx_adm___18 Mental Health Illness<br>98 preghx_adm___98 Other Infection<br>99 preghx_adm___99 None
	65	[placenta_adm]	Any known/suspected placental disorder (this pregnancy)	checkbox<br>1 placenta_adm___1 Yes, placenta previa<br>2 placenta_adm___2 Yes, placental abruption<br>97 placenta_adm___97 Yes, other (specify)<br>98 placenta_adm___98 No<br>99 placenta_adm___99 Not sure
	66	[placentaother_adm]<br>Show the field ONLY if:<br>[placenta_adm(97)] = '1'	Specify other	text
	67	[medhbp_adm]	Is she currently taking medication for high blood pressure?	dropdown<br>1 Yes<br>2 No<br>99 Not sure

	68	[medarv_adm]<br><br>Show the field ONLY if:<br>[medhx_adm(1)] = '1' or [preg hx_adm(11)] = '1'	Is she currently taking ARVs for HIV treatment?	dropdown<br>1 Yes<br>2 No<br>99 Not sure
	69	[covidvax_adm]	Has woman been vaccinated against COVID-19	dropdown<br>1 Yes - Fully vaccinated (completion DURING pregnancy)<br>2 Yes - Fully vaccinated (completion BEFORE pregnancy)<br>3 Yes - Currently partially vaccinated<br>4 No - Not vaccinated
	70	[prevadm_adm]	Section Header: *Hospitalization During Pregnancy*<br>Has the woman been admitted to hospital DURING this pregnancy for any reason?	yesno<br>1 Yes<br>0 No
	71	[lngth_prevadm_adm]<br><br>Show the field ONLY if:<br>[prevadm_adm]='1'	How long ago was this admission?	dropdown<br>1 1 - 7 days (in the past week)<br>2 7 - 28 days (from one week to one month ago)<br>3 28 days - 6 months (from one month to six months ago)<br>4 > 6 months ago (more than six months ago)
	72	[rs_prevadm_adm]<br><br>Show the field ONLY if:<br>[prevadm_adm]='1'	What was the reason for this admission?	checkbox<br>1 rs_prevadm_adm___1 Gestational diabetes<br>2 rs_prevadm_adm___2 Pre-eclampsia<br>3 rs_prevadm_adm___3 Eclampsia<br>4 rs_prevadm_adm___4 Gestational hypertension<br>5 rs_prevadm_adm___5 Antepartum hemorrhage/ vaginal bleeding<br>6 rs_prevadm_adm___6 PPROM<br>7 rs_prevadm_adm___7 Preterm labour<br>8 rs_prevadm_adm___8 Malaria<br>9 rs_prevadm_adm___9 HIV<br>10 rs_prevadm_adm___10 Urinary tract infection<br>11 rs_prevadm_adm___11 Tuberculosis<br>12 rs_prevadm_adm___12 Anemia<br>13 rs_prevadm_adm___13 Abuse/Fight<br>14 rs_prevadm_adm___14 motor accident (car, boda, etc.)<br>98 rs_prevadm_adm___98 Other Infection<br>99 rs_prevadm_adm___99 Other (specify)
	73	[rs_prevadm2_adm]<br><br>Show the field ONLY if:<br>[rs_prevadm_adm(99)] = '1' or [rs_prevadm_adm(98)] = '1'	Specify other	text
	74	[prevadm2_adm]<br><br>Show the field ONLY if:<br>[prevadm_adm] = '1'	For how many days was the woman admitted	text (integer, Min: 1, Max: 100)
	75	[uti_abx_adm]<br><br>Show the field ONLY if:<br>[rs_prevadm_adm(10)] = '1' or [rs_prevadm_adm(11)] = '1' or [rs_prevadm_adm(8)] = '1' or [rs_prevadm_adm(98)] = '1'	Was she given IV antibiotics/antimalarials during the admission?	yesno<br>1 Yes<br>0 No

	#	Variable / Field Name	Field Label	Field Attributes
	76	[numberanc_adm]	Section Header: *ANC visit history*<br>How many ANC visits did you attend (best estimate if unsure)?	dropdown<br>0 0<br>1 1<br>2 2<br>3 3<br>4 4<br>5 5<br>6 6<br>7 7<br>8 8<br>9 >8
	77	[ancprovider_adm]	Which types of healthcare providers provided your ANC care (choose all that apply)	checkbox<br>1 ancprovider_adm___1 Obstetrician<br>2 ancprovider_adm___2 Medical Officer<br>3 ancprovider_adm___3 Clinical Officer<br>4 ancprovider_adm___4 Nurse<br>5 ancprovider_adm___5 midwife<br>6 ancprovider_adm___6 Traditional Birth Attendant<br>7 ancprovider_adm___7 Other
	78	[comment_adm]	Comment	notes
	79	[pregnancy_history_complete]	Section Header: *Form Status*<br>Complete?	dropdown<br>0 Incomplete<br>1 Unverified<br>2 Complete

Instrument: **4. SES and Demographics** (ses_and_demographics)

	#	Variable / Field Name	Field Label	Field Attributes
	80	[housenum_ses]	Section Header: *SES and Demographics*<br>How many people will be living in your household (including you AND new baby)?	text (integer, Min: 0, Max: 25)
	81	[numchild_ses]	How many children are you living with in your household (including the new baby)?	text (integer, Min: 0, Max: 15)
	82	[marry_ses]	What is your current marital status	dropdown<br>1 Married monogamous<br>2 Married polygamous<br>3 Single<br>4 Separated/divorced<br>5 Widowed
	83	[livfather_ses]	Do you live with the father of this baby?	yesno<br>1 Yes<br>0 No
	84	[schoolyrs_ses]	What is the highest level of education you (mother) have completed?	dropdown<br>1 No school<br>2 < = P3<br>3 P4-P7<br>4 S1-S6<br>5 Post secondary (including post S4 technical school)<br>6 Don't know

	85	[nutri_adm]	Section Header: *SES Index Questions*<br>During your pregnancy, did you or any household member have to eat less food than you felt you needed?	dropdown<br>1 Yes<br>2 No<br>99 Not Sure
	86	[sesindex_flooring]	Which types of flooring does ANY room in your home have (choose ALL that apply)?	checkbox<br>1 sesindex_flooring___1 Earth/Dung/Sand<br>2 sesindex_flooring___2 Temporary carpet<br>3 sesindex_flooring___3 Permanent flooring (tile, finished wood)<br>4 sesindex_flooring___4 Cement<br>98 sesindex_flooring___98 Other
	87	[sesindex_toilet]	What kind of toilet do members of your household usually use?	checkbox<br>1 sesindex_toilet___1 Flush toilet system (sitting or squatting)<br>2 sesindex_toilet___2 Ventilated Improved Pit (VIP) Latrine<br>3 sesindex_toilet___3 Composting toilet<br>4 sesindex_toilet___4 Pit latrine<br>5 sesindex_toilet___5 Bucket toilet<br>6 sesindex_toilet___6 No toilet<br>98 sesindex_toilet___98 Other
	88	[sesindex_toiletshared]<br>Show the field ONLY if: [sesindex_toilet(1)] = "1" OR [sesindex_toilet(2)] = "1" OR [sesindex_toilet(3)] = "1"	Do you share this toilet with anyone outside your household (i.e. those NOT living together with you)?	yesno<br>1 Yes<br>0 No
	89	[sesindex_cooking]	Which of the following energy sources for cooking food do you use (choose all that apply)?	checkbox<br>1 sesindex_cooking___1 Electricity<br>2 sesindex_cooking___2 Petrol/diesel/propane<br>3 sesindex_cooking___3 Kerosene/paraffin<br>4 sesindex_cooking___4 Coal/charcoal<br>5 sesindex_cooking___5 Wood<br>6 sesindex_cooking___6 Straw/shrubs/grass<br>7 sesindex_cooking___7 Biomass or woodchips<br>8 sesindex_cooking___8 Dung<br>9 sesindex_cooking___9 Other
	90	[sesindex_safewater]	What is the main source of drinking water for members of your household?	dropdown<br>1 Piped water<br>2 Borehole<br>3 Protected spring<br>4 Well/dam<br>5 Rainwater<br>6 River/lake<br>7 Bottled water<br>98 Other
	91	[sesindex_otherwater]<br>Show the field ONLY if: [sesindex_safewater] = '98'	Specify other:	text
	92	[sesindex_safewaterdistance]	Does it take you more than 30 minutes to walk from your home to the water source and back?	yesno<br>1 Yes<br>0 No

	93	[sesindex_assets]	Does your household have any of the following items:	checkbox<br>1 sesindex_assets___1 Electricity (from the grid)<br>2 sesindex_assets___2 A television<br>3 sesindex_assets___3 A computer<br>4 sesindex_assets___4 A refrigerator<br>99 sesindex_assets___99 None
	94	[sesindex_assets2]	Do you (or someone in your household) own any of the following items:	checkbox<br>1 sesindex_assets2___1 A smart phone<br>2 sesindex_assets2___2 A non-smart phone<br>3 sesindex_assets2___3 A bicycle<br>4 sesindex_assets2___4 A motorcycle<br>5 sesindex_assets2___5 A car or truck<br>99 sesindex_assets2___99 None
	95	[sesindex_room]	How many rooms does your home contain?	radio<br>1 One<br>2 Two<br>3 Three<br>4 More than three
	96	[child_death_ses]	Have you had any children who have died?	yesno<br>1 Yes<br>0 No
	97	[comment_ses]	Comment	notes
	98	[ses_and_demographics_complete]	Section Header: *Form Status*<br>Complete?	dropdown<br>0 Incomplete<br>1 Unverified<br>2 Complete
Instrument: **5. Delivery Maternal** (delivery_maternal)				
	99	[numbabe_del]	How many babies were delivered?	text (integer, Min: 1, Max: 3)
	100	[prom_del]	Rupture of membranes more than 24hrs before delivery?	dropdown<br>1 Yes<br>2 No<br>3 Not sure
	101	[rom_del]<br>Show the field ONLY if:<br>[prom_del] = '1'	For how many hours were membranes ruptured prior to delivery?	dropdown<br>1 0 - 4 hours<br>2 4 - 8 hours<br>3 8 -12 hours<br>4 12 - 24 hours<br>5 > 24 hours
	102	[deldate_del]	Delivery date	text (date_dmy)
	103	[deltime_del]	Delivery time (USE 24 HOUR TIME - HH:MM)	text (time)
	104	[delmode_del]	What was the mode of delivery?	dropdown<br>1 Vaginal<br>2 Assisted vaginal (vacuum or forceps)<br>3 Caesarean (with labour)<br>4 Caesarean (without labour)
	105	[episiotomy_del]	Was the woman given an episiotomy?	dropdown<br>1 Yes<br>2 No<br>99 Not sure

	106	[tear_del]	Was there any noted vaginal or perineal tearing?	dropdown<br>1 Yes<br>2 No<br>99 Not sure
	107	[degreetear_del]<br>Show the field ONLY if:<br>[tear_del] = '1'	What was the recorded degree of tearing?	dropdown<br>1 1<br>2 2<br>3 3<br>4 4
	108	[induce_del]	Was the start of labour induced?	dropdown<br>1 Yes<br>2 No<br>99 Not sure
	109	[inductype_del]<br>Show the field ONLY if:<br>[induce_del] = '1'	Which method of induction was used (select all that apply)?	checkbox<br>1 inductype_del___1 Membrane strip and sweep<br>2 inductype_del___2 Manual rupture of membranes<br>3 inductype_del___3 Foley catheter<br>4 inductype_del___4 Prostaglandins<br>5 inductype_del___5 Oxytocin<br>98 inductype_del___98 Other
	110	[pph_del]	Was a PPH noted?	dropdown<br>1 Yes<br>2 No<br>99 Not sure
	111	[transfx_del]	Was the woman given a blood transfusion?	dropdown<br>1 Yes<br>2 No<br>99 Not sure
	112	[unittrans_del]<br>Show the field ONLY if:<br>[transfx_del] = '1'	How many units of blood?	text (integer, Min: 1, Max: 30)
	113	[obstruct_del]	Was obstructed labour noted?	dropdown<br>1 Yes<br>2 No<br>99 Not sure
	114	[meconium_del]	Was any meconium noted in the amniotic fluid during labour?	dropdown<br>1 Yes<br>2 No<br>99 Not sure

	115	[vagexam_del]	Number of vaginal exams (self reported best estimate)	dropdown<br>0 0<br>1 1<br>2 2<br>3 3<br>4 4<br>5 5<br>6 6<br>7 7<br>8 8<br>9 9<br>10 >9
	116	[placenta_del]	Placental complications noted?	dropdown<br>1 Placenta previa<br>2 Placenta Abruption<br>3 Placenta accreta<br>4 Retained placenta<br>99 None
	117	[man_placenta_del]	Manual removal of placenta	yesno<br>1 Yes<br>0 No
	118	[other_del]	Other major event or conditions during delivery?	notes
	119	[csecturgency_del]<br>Show the field ONLY if: [delmode_del] = '3' or [delmode_del] = '4'	Section Header: *Caesarian Section Variables*<br>Surgical urgency (per clinical team)	dropdown<br>1 Immediate threat to life of woman or fetus<br>2 Maternal or fetal compromise which is not immediately life-threatening<br>3 Needing early delivery but no maternal or fetal compromise<br>4 At a time to suit the patient and maternity team<br>99 Not sure
	120	[csect_delay_del]<br>Show the field ONLY if: [delmode_del] = '3' or [delmode_del] = '4'	Estimated decision to delivery time	dropdown<br>1 No delay<br>2 30-60m<br>3 61m - 120m<br>4 >120m<br>5 Not sure
	121	[heme_del]<br>Show the field ONLY if: [delmode_del] = '3' or [delmode_del] = '4'	Pre-surgery hemoglobin level	text
	122	[abx_del]<br>Show the field ONLY if: [delmode_del] = '3' or [delmode_del] = '4'	Were prophylactic antibiotics administered?	dropdown<br>1 Yes - within 1 hour of incision<br>2 Yes - but after incision<br>3 No<br>99 Not sure

	123	[csect_abx_del]<br><br>Show the field ONLY if:<br>[abx_del] = '2' or [abx_del] = '1<br>'	List all antibiotics used for prophylaxis	dropdown<br>1 IV Cefazolin<br>2 IV Ampicillin or Amoxicillin<br>3 IV cloxacilllin<br>4 IV penicillin<br>5 IV ceftriaxone<br>6 IV gentamicin<br>7 IV vancomycin<br>8 IV or PO metronidazole<br>9 IV or PO Clindamycin<br>98 Other (specify)<br>99 None
	124	[otherabx_del]<br><br>Show the field ONLY if:<br>[csect_abx_del] = '98'	Specify other antibiotic	text
	125	[anticoag_del]<br><br>Show the field ONLY if:<br>[delmode_del] = '3' or [delmo<br>de_del] = '4'	Were anticoagulants administered?	yesno<br>1 Yes<br>0 No
	126	[delivery_maternal_complet<br>e]	Section Header: *Form Status*<br>Complete?	dropdown<br>0 Incomplete<br>1 Unverified<br>2 Complete
Instrument: **6. Delivery Neonatal** (delivery_neonatal)				
	127	[infantstatus_del]	Section Header: *Details of Delivery*<br>Was the baby born alive?	yesno<br>1 Yes<br>0 No
	128	[sb20wk_del]<br><br>Show the field ONLY if:<br>[infantstatus_del] = '0'	Is stillbirth assumed to be >20 weeks gestation?	yesno<br>1 Yes<br>0 No
	129	[sb_del]<br><br>Show the field ONLY if:<br>[infantstatus_del] = '0'	Type of stillbirth	dropdown<br>1 Fresh<br>2 Macerated<br>99 Not sure
	130	[sbcongenital_del]<br><br>Show the field ONLY if:<br>[infantstatus_del] = '0'	Presence of visible congenital malformation	dropdown<br>1 Yes<br>2 No<br>3 Not sure
	131	[sbsex_del]<br><br>Show the field ONLY if:<br>[infantstatus_del] = '0'	Sex of the stillborn baby	dropdown<br>1 Male<br>2 Female<br>99 Not sure
	132	[sexbb_del]<br><br>Show the field ONLY if:<br>[infantstatus_del] = '1'	Sex of the baby	dropdown<br>1 Male<br>2 Female<br>98 Other
	133	[apgar1_del]<br><br>Show the field ONLY if:<br>[infantstatus_del] = '1'	Apgar score after 1 minute	text (integer, Min: 1, Max: 10)

	134	[apgar5_del]<br><br>Show the field ONLY if:<br>[infantstatus_del] = '1'	Apgar score after 5 minutes	text (integer, Min: 1, Max: 10)
	135	[cord_delay_del]	Did the patient experience delayed cord clamping (> 1 minute)?	dropdown<br>1 Yes<br>2 No<br>3 Not sure
	136	[weightbb_del]<br><br>Show the field ONLY if:<br>[infantstatus_del] = '1'	Weight of the baby (kg)	text (number)
	137	[height_neo]<br><br>Show the field ONLY if:<br>[infantstatus_del] = '1'	Length (cm)	text (number)
	138	[rescus_del]<br><br>Show the field ONLY if:<br>[infantstatus_del] = '1'	Any rescucitation at birth?	dropdown<br>1 Yes<br>2 No<br>99 Not sure
	139	[resustype_del]<br><br>Show the field ONLY if:<br>[rescus_del] = '1'	What type of resuscitation did the baby receive after birth? (check all that apply)	checkbox<br>1 resustype_del___1 Stimulation only<br>2 resustype_del___2 Oxygen<br>3 resustype_del___3 Bag mask<br>4 resustype_del___4 Suction<br>5 resustype_del___5 Drugs<br>6 resustype_del___6 Warming<br>7 resustype_del___7 Chest compression<br>8 resustype_del___8 Don't know<br>98 resustype_del___98 Other<br>99 resustype_del___99 None
	140	[delivery_neonatal_complete]	Section Header: *Form Status*<br>Complete?	dropdown<br>0 Incomplete<br>1 Unverified<br>2 Complete

Instrument: **7. Discharge Interview Maternal** (discharge_interview_maternal)

	141	[disnurse_mat]	Nurse collecting the data	dropdown<br>1 Olivia<br>2 Phionah<br>3 Jonan<br>4 Annet Happy<br>5 Annet Mary<br>6 Miria<br>7 Maureen<br>8 Clare<br>9 Kelemensia<br>10 Immaculate<br>11 Bosco<br>12 Enid Kibone<br>98 Other
	142	[disnurseother_mat]<br><br>Show the field ONLY if:<br>[disnurse_mat] = '98'	Specify other nurse:	text

	143	[adm_mat]	Section Header: *Maternal Clinical Exam Before Discharge*<br>Was mother admitted to higher level of care due to complications AFTER delivery	yesno<br>1 Yes<br>0 No
	144	[dischstat_mat]	Discharge status	dropdown<br>1 Routine<br>2 Against medical advice<br>3 Died<br>99 Other
	145	[sbp_mat]<br>Show the field ONLY if:<br>[dischstat_mat] != '3'	Discharge systolic blood pressure	text (integer)
	146	[dbp_mat]<br>Show the field ONLY if:<br>[dischstat_mat] != '3'	Discharge diastolic blood pressure	text (integer)
	147	[rr_mat]<br>Show the field ONLY if:<br>[dischstat_mat] != '3'	Respiratory rate	text (integer)
	148	[temp_mat]<br>Show the field ONLY if:<br>[dischstat_mat] != '3'	Temporal artery temperature	text (number_1dp, Min: 36, Max: 41)
	149	[best_spo2_mat]<br>Show the field ONLY if:<br>[dischstat_mat] != '3'	Best SpO2	text (integer, Min: 60, Max: 100)
	150	[best_hr_mat]<br>Show the field ONLY if:<br>[dischstat_mat] != '3'	Best Heart rate	text (integer)
	151	[best_oxyradg_mat]<br>Show the field ONLY if:<br>[dischstat_mat] != '3' and [site_adm] = '2'	Rad-G: Best SpO2 from Rad-G	text (integer, Min: 60, Max: 100)
	152	[best_hrradg_mat]<br>Show the field ONLY if:<br>[dischstat_mat] != '3' and [site_adm] = '2'	Rad-G: best heart rate (PR) from Rad-G	text (integer)
	153	[tempradg_mat]<br>Show the field ONLY if:<br>[dischstat_mat] != '3' and [site_adm] = '2'	Rad-G: Temporal artery temperature from Rad-G	text (number_1dp, Min: 36, Max: 41)
	154	[rrradg_mat]<br>Show the field ONLY if:<br>[dischstat_mat] != '3' and [site_adm] = '2'	Rad-G: Respiratory rate (RRp) from Rad-G	text (integer)
	155	[hem_mat]<br>Show the field ONLY if:<br>[dischstat_mat] != '3'	Hematocrit (%)	text (integer)
	156	[destination_mat]<br>Show the field ONLY if:<br>[dischstat_mat] != '3'	Discharge destination	dropdown<br>1 Own home<br>2 Home of relative<br>3 Home of parent (of woman)<br>4 Home of friend<br>5 Mother not yet sure<br>98 Other
	157	[dischdate_mat]	Date of discharge	text (date_dmy)
	158	[deathdate_mat]	(Temporary) Date of death	text

	159	[dischtime_mat]<br>Show the field ONLY if:<br>[dischstat_mat] != '3'	Time of discharge (USE 24 HOUR TIME)	text (time)
	160	[support_mat]<br>Show the field ONLY if:<br>[dischstat_mat] != '3'	Please indicate all those from whom you have substantial support at home after discharge (choose all that apply)	checkbox<br>1 — support_mat___1 — Mother<br>2 — support_mat___2 — Mother-in-law<br>3 — support_mat___3 — Husband/partner/father<br>4 — support_mat___4 — Other relative (sister/cousin, etc)<br>5 — support_mat___5 — Friend<br>99 — support_mat___99 — No substantial support at home
	161	[bf_mat]<br>Show the field ONLY if:<br>[dischstat_mat] != '3'	Any prior history of poor milk production?	dropdown<br>1 — Yes<br>2 — No<br>3 — Not Applicable (first baby)
	162	[symp_mat]<br>Show the field ONLY if:<br>[dischstat_mat] != '3'	Symptoms currently present (check all that apply)	checkbox<br>1 — symp_mat___1 — Headache (continuous pain in the front of the head or behind the eyes)<br>2 — symp_mat___2 — Visual changes (spots, flashing lights or blurry vision)<br>3 — symp_mat___3 — Chest pain (causing difficulty breathing)<br>4 — symp_mat___4 — Shortness of breath<br>5 — symp_mat___5 — Nausea with vomiting<br>6 — symp_mat___6 — Abdominal pain on the right side<br>7 — symp_mat___7 — Foul smelling vaginal discharge<br>8 — symp_mat___8 — Stiff neck<br>9 — symp_mat___9 — Cough<br>10 — symp_mat___10 — Difficulty emptying bladder<br>99 — symp_mat___99 — None
	163	[abx_mat]	During this admission was woman given antibiotics?	checkbox<br>1 — abx_mat___1 — Yes - oral antibiotics<br>2 — abx_mat___2 — Yes - IV antibiotics<br>3 — abx_mat___3 — No antibiotics<br>99 — abx_mat___99 — Not sure
	164	[followup_date_mat]	Scheduled follow-up date (for phone follow-up with research team)	text (date_dmy)
	165	[comment_mat]	Comment	notes
	166	[best_sqi_mat]	Section Header: *HIDDEN SYSTEM SECTION (Please ignore during data entry)*<br>Best SQI	text (integer)
	167	[best_spo2trends_mat]	Best SpO2 Trends file	file
	168	[best_spo2raw_mat]	Best SpO2 Raw bin file	file
	169	[add_sqi_mat]	Additional SQI	text (integer)
	170	[add_spo2trends_mat]	Additional SpO2 Trends file	file
	171	[add_spo2raw_mat]	Additional SpO2 Raw bin file	file
	172	[rrtaps_mat]	Respiratory rate taps	notes

173	[discharge_interview_maternal_complete]	Section Header: *Form Status*<br>Complete?	dropdown<br>0 Incomplete<br>1 Unverified<br>2 Complete
Instrument: **8. Discharge Interview Neonatal** (discharge_interview_neonatal)			
174	[dispbb_neo]	Section Header: *Neonatal Clinical Exam Before Discharge*<br>Disposition of baby?	dropdown<br>1 Discharged<br>2 Admitted<br>3 Died<br>4 Referred<br>5 Discharged, but readmitted after study nurse assessment<br>98 Other
175	[dispbb_other_neo]<br>Show the field ONLY if:<br>[dispbb_neo] = '98'	Specify other:	text
176	[dispdate_neo]	Disposition date	text (date_dmy)
177	[admitsite_neo]<br>Show the field ONLY if:<br>[dispbb_neo] <> '' and [dispbb_neo] <> '1' and [dispbb_neo] <> '3'	Where was the child admitted?	dropdown<br>1 Delivery hospital<br>2 Different hospital
178	[admitdisposition_neo]<br>Show the field ONLY if:<br>[dispbb_neo] <> '' and [dispbb_neo] <> '1' and [dispbb_neo] <> '3'	Admission disposition	dropdown<br>1 Died<br>2 Discharged<br>3 Referred<br>4 Discharge against medical advice/fled
179	[admitdispdate_neo]<br>Show the field ONLY if:<br>[dispbb_neo] <> '' and [dispbb_neo] <> '1' and [dispbb_neo] <> '3'	Admission disposition date	text (date_dmy)
180	[admitdiagnosis_neo]<br>Show the field ONLY if:<br>[dispbb_neo] <> '' and [dispbb_neo] <> '1' and [dispbb_neo] <> '3'	Admission diagnosis	checkbox<br>1 admitdiagnosis_neo___1 Sepsis<br>2 admitdiagnosis_neo___2 Birth Asphyxia<br>3 admitdiagnosis_neo___3 Congenital<br>4 admitdiagnosis_neo___4 Jaundice<br>5 admitdiagnosis_neo___5 prematurity/low birth weight<br>99 admitdiagnosis_neo___99 Other (specify)
181	[admitdiagnosisother_neo]<br>Show the field ONLY if:<br>[admitdiagnosis_neo(99)] = '1'	Other admission diagnosis:	text
182	[babedisch_neo]<br>Show the field ONLY if:<br>[dispbb_neo] = '1'	Is the baby being discharged home with mom?	yesno<br>1 Yes<br>0 No
183	[poop_neo]<br>Show the field ONLY if:<br>[babedisch_neo]='1'	Has your baby pooped?	radio<br>1 Yes<br>2 No<br>3 Don't know

	184	[pee_neo]<br><br>Show the field ONLY if:<br>[babedisch_neo]='1'	Has your baby peed?	radio<br>1 Yes<br>2 No<br>3 Don't know
	185	[bf_neo]<br><br>Show the field ONLY if:<br>[babedisch_neo] = '1'	Is baby latching and suckling well when breastfeeding?	dropdown<br>1 Yes<br>2 No
	186	[jaundice_neo]<br><br>Show the field ONLY if:<br>[babedisch_neo] = '1'	Are the baby's eyes yellow?	dropdown<br>0 No<br>1 Yes<br>2 Yes (a little)
	187	[eyedischarge_neo]<br><br>Show the field ONLY if:<br>[babedisch_neo] = '1'	Does the baby have a discharge in their eyes?	yesno<br>1 Yes<br>0 No
	188	[temp1_neo]<br><br>Show the field ONLY if:<br>[babedisch_neo]='1'	Temporal artery temperature 1	text (number_1dp, Min: 36, Max: 41)
	189	[temp2_neo]<br><br>Show the field ONLY if:<br>[babedisch_neo]='1'	Temporal artery temperature 2	text (number_1dp, Min: 36, Max: 41)
	190	[rr_neo]<br><br>Show the field ONLY if:<br>[babedisch_neo]='1'	Respiratory rate	text (integer)
	191	[foot_o2src_neo]<br><br>Show the field ONLY if:<br>[babedisch_neo]='1'	Foot Oxygen Saturation Source	radio<br>1 Using tablet<br>2 Separate device
	192	[foot_best_spo2_neo]<br><br>Show the field ONLY if:<br>[babedisch_neo]='1'	Best Foot SpO2	text (integer, Min: 60, Max: 100)
	193	[foot_best_hr_neo]<br><br>Show the field ONLY if:<br>[babedisch_neo]='1'	Best Foot Heart rate	text (integer)
	194	[foot_best_oxyradg_neo]<br><br>Show the field ONLY if:<br>[babedisch_neo]='1' and [site_adm] = '2'	Rad-G: Best Foot SpO2 from Rad-G	text (integer, Min: 60, Max: 100)
	195	[foot_best_hrradg_neo]<br><br>Show the field ONLY if:<br>[babedisch_neo]='1' and [site_adm] = '2'	Rad-G: Best Foot Heart rate (PR) from Rad-G	text (integer)
	196	[tempfootradg_neo]<br><br>Show the field ONLY if:<br>[babedisch_neo]='1' and [site_adm] = '2'	Rad-G: Foot Temporal artery temperature from Rad-G	text (number_1dp, Min: 36, Max: 41)
	197	[rrfootradg_neo]<br><br>Show the field ONLY if:<br>[babedisch_neo]='1' and [site_adm] = '2'	Rad-G: Foot Respiratory rate (RRp) from Rad-G	text (integer)
	198	[rhand_o2src_neo]<br><br>Show the field ONLY if:<br>[babedisch_neo]='1'	Right Hand Oxygen Saturation Source	radio<br>1 Using tablet<br>2 Separate device

	199	[rhand_best_spo2_neo]<br><br>Show the field ONLY if:<br>[babedisch_neo]='1'	Best Right Hand SpO2	text (integer, Min: 60, Max: 100)
	200	[rhand_best_hr_neo]<br><br>Show the field ONLY if:<br>[babedisch_neo]='1'	Best Right Hand Heart rate	text (integer)
	201	[rhand_best_oxyradg_neo]<br><br>Show the field ONLY if:<br>[babedisch_neo]='1' and [site_adm] = '2'	Rad-G: Best Right Hand SpO2 from Rad-G	text (integer, Min: 60, Max: 100)
	202	[rhand_best_hrradg_neo]<br><br>Show the field ONLY if:<br>[babedisch_neo]='1' and [site_adm] = '2'	Rad-G: Best Right Hand Heart rate (PR) from Rad-G	text (integer)
	203	[temphandradg_neo]<br><br>Show the field ONLY if:<br>[babedisch_neo]='1' and [site_adm] = '2'	Rad-G: Hand Temporal artery temperature from Rad-G	text (number_1dp, Min: 36, Max: 41)
	204	[rrhandradg_neo]<br><br>Show the field ONLY if:<br>[babedisch_neo]='1' and [site_adm] = '2'	Rad-G: Hand Respiratory rate (RRp) from Rad-G	text (integer)
	205	[abx_neo]<br><br>Show the field ONLY if:<br>[babedisch_neo] = '1'	Was the baby discharged on antibiotics?	yesno<br>1 Yes<br>0 No
	206	[comment_neo]	Comment	notes
	207	[foot_best_sqi_neo]<br><br>Show the field ONLY if:<br>[babedisch_neo]='1'	Section Header: *HIDDEN SYSTEM SECTION (Please ignore during data entry)*<br><br>Best Foot SQI	text (integer)
	208	[foot_best_spo2trends_neo]<br><br>Show the field ONLY if:<br>[babedisch_neo]='1'	Best Foot SpO2 Trends file	file
	209	[foot_best_spo2raw_neo]<br><br>Show the field ONLY if:<br>[babedisch_neo]='1'	Best Foot SpO2 Raw bin file	file
	210	[foot_add_sqi_neo]<br><br>Show the field ONLY if:<br>[babedisch_neo]='1'	Additional Foot SQI	text (integer)
	211	[foot_add_spo2trends_neo]<br><br>Show the field ONLY if:<br>[babedisch_neo]='1'	Additional Foot SpO2 Trends file	file
	212	[foot_add_spo2raw_neo]<br><br>Show the field ONLY if:<br>[babedisch_neo]='1'	Additional Foot SpO2 Raw bin file	file
	213	[rhand_best_sqi_neo]<br><br>Show the field ONLY if:<br>[babedisch_neo]='1'	Best Right Hand SQI	text (integer)
	214	[rhand_best_spo2trends_neo]<br><br>Show the field ONLY if:<br>[babedisch_neo]='1'	Best Right Hand SpO2 Trends file	file
	215	[rhand_best_spo2raw_neo]<br><br>Show the field ONLY if:<br>[babedisch_neo]='1'	Best Right Hand SpO2 Raw bin file	file

	216	[rhand_add_sqi_neo]<br>Show the field ONLY if:<br>[babedisch_neo]='1'	Additional Right Hand SQI	text (integer)
	217	[rhand_add_spo2trends_neo]<br>Show the field ONLY if:<br>[babedisch_neo]='1'	Additional Right Hand SpO2 Trends file	file
	218	[rhand_add_spo2raw_neo]<br>Show the field ONLY if:<br>[babedisch_neo]='1'	Additional Right Hand SpO2 Raw bin file	file
	219	[rrtaps_neo]<br>Show the field ONLY if:<br>[babedisch_neo]='1'	Respiratory rate taps	notes
	220	[discharge_interview_neonatal_complete]	Section Header: *Form Status*<br>Complete?	dropdown<br>0 Incomplete<br>1 Unverified<br>2 Complete
Instrument: **10. Six Week Follow Up Maternal** (six_week_follow_up_maternal)				
	221	[intro_swf]	[firstname_adm_v2] [lastname_adm_v2], DOB: [dob_adm_v2]<br>Admitted at [site_adm] on [admitdate_adm_v2]<br>Discharged on [dischdate_mat]<br>Phone: [phone_adm_v2]<br>Alternative Phone: [otherphone_adm_v2]<br>From [villageresidence_adm_v2] in [parishresidence_adm_v2], [subcountyresidence_adm_v2], [districtresidence_adm_v2]	descriptive
	222	[considerate_swf]<br>Show the field ONLY if:<br>[dischstat_mat] = '3'	Please be considerate: the woman died in hospital	descriptive
	223	[considerate2_swf]<br>Show the field ONLY if:<br>[dispbb_neo] = '3'	Please be considerate: one of the delivered babies died in hospital	descriptive
	224	[physical_swf]<br>Show the field ONLY if:<br>[dischstat_mat] != '3'	Check this box if this is a physical (in-person) follow-up	checkbox<br>1 physical_swf___1
	225	[respondent_swf]<br>Show the field ONLY if:<br>[dischstat_mat] != '3'	Respondent during the interview?	dropdown<br>1 Woman herself<br>98 Other
	226	[otherrespondent_swf]<br>Show the field ONLY if:<br>[respondent_swf] = '98'	Specify other	dropdown<br>1 Husband<br>2 Mother in law<br>3 Mother<br>4 Sibling<br>5 Father<br>6 Father in law<br>7 other relative/Friend<br>98 Other
	227	[home_swf]<br>Show the field ONLY if:<br>[dischstat_mat] != '3'	Is mother based at the same home that you were in prior to delivery (6 weeks after birth)?	yesno<br>1 Yes<br>0 No
	228	[momalive_swf]<br>Show the field ONLY if:<br>[dischstat_mat] != '3'	Section Header: *Maternal Vital Status*<br>Is the woman alive at the time of follow-up interview?	yesno<br>1 Yes<br>0 No

	229	[momdeathdate_swf]<br><br>Show the field ONLY if:<br>[momalive_swf] = '0'	On what date did she die?	text (date_dmy)
	230	[momdeathplace_swf]<br><br>Show the field ONLY if:<br>[momalive_swf] = '0'	Where did she die?	dropdown<br>1 Home<br>2 Hospital<br>3 On the way to hospital<br>98 Other
	231	[momadmit_swf]<br><br>Show the field ONLY if:<br>[momalive_swf] = '1'	Section Header: *Health seeking/readmissions*<br>Was the mother admitted for one or more nights at a facility for any reason after being home post-delivery?	yesno<br>1 Yes<br>0 No
	232	[nummomadmit_swf]<br><br>Show the field ONLY if:<br>[momadmit_swf] = '1'	How many times was the mother admitted in the first 6 weeks after delivery?	dropdown<br>1 1<br>2 2<br>3 3<br>4 >3
	233	[momadmitdate_swf]<br><br>Show the field ONLY if:<br>[momadmit_swf] = '1'	Date of first admission	text (date_dmy)
	234	[momnightsadm_swf]<br><br>Show the field ONLY if:<br>[momadmit_swf] = '1'	For how many nights was she admitted?	text (integer, Min: 1)
	235	[momadmitpathway_swf]<br><br>Show the field ONLY if:<br>[momadmit_swf] = '1'	What was the care-seeking pathway to the first admission?	checkbox<br>1 momadmitpathway_swf___1 Routine PNC visit identified problem<br>2 momadmitpathway_swf___2 Self referral<br>3 momadmitpathway_swf___3 Both self-referral and PNC visit resulted in care seeking<br>98 momadmitpathway_swf___98 Other
	236	[momadmitpathwayother_swf]<br><br>Show the field ONLY if:<br>[momadmitpathway_swf(98)] = '1'	Other pathway:	text

	237	[momadmitsymp_swf]<br><br>Show the field ONLY if:<br>[momadmit_swf] = '1'	What were the symptoms she experienced during this illness? (check all that apply)	checkbox<br>1 momadmitsymp_swf___1 Heavy vaginal bleeding<br>2 momadmitsymp_swf___2 Bleeding from nose/ eyes/ ears<br>3 momadmitsymp_swf___3 Petechiae (small red dots and bruises across the skin)<br>4 momadmitsymp_swf___4 Abnormal tiredness<br>5 momadmitsymp_swf___5 Convulsions<br>6 momadmitsymp_swf___6 Shortness of breath<br>7 momadmitsymp_swf___7 Changes in vision<br>8 momadmitsymp_swf___8 Severe headache < 24 hrs<br>9 momadmitsymp_swf___9 Severe headache >24 hrs<br>10 momadmitsymp_swf___10 Abdominal pain<br>11 momadmitsymp_swf___11 Abdominal tenderness when touched<br>12 momadmitsymp_swf___12 Foul smelling vaginal discharge<br>13 momadmitsymp_swf___13 Fever/ body hotness < 7days<br>14 momadmitsymp_swf___14 Fever/ body hotness >7days<br>15 momadmitsymp_swf___15 Diarrhea < 14 days<br>16 momadmitsymp_swf___16 Diarrhea >14 days<br>17 momadmitsymp_swf___17 Vomiting everything<br>18 momadmitsymp_swf___18 Cough < 14 days<br>19 momadmitsymp_swf___19 Cough >14 days<br>20 momadmitsymp_swf___20 Yellow eyes<br>21 momadmitsymp_swf___21 Loss of consciousness<br>98 momadmitsymp_swf___98 Other (specify)<br>99 momadmitsymp_swf___99 None
	238	[momadmitsympother_swf]<br><br>Show the field ONLY if:<br>[momadmitsymp_swf(98)] = '1 '	Other symptom	text

	#	Variable / Field Name	Field Label	Field Attributes
	239	[momadmitcond_swf]<br>Show the field ONLY if:<br>[momadmit_swf] = '1'	Was she told she had any of the following conditions during this visit? (check all that apply)	checkbox<br>1 momadmitcond_swf___1 Pre-eclampsia<br>2 momadmitcond_swf___2 Eclampsia<br>3 momadmitcond_swf___3 Diabetes<br>4 momadmitcond_swf___4 Fistula<br>5 momadmitcond_swf___5 HIV related illness<br>6 momadmitcond_swf___6 Malaria<br>7 momadmitcond_swf___7 Retained placenta<br>8 momadmitcond_swf___8 Heavy bleeding<br>9 momadmitcond_swf___9 Anemia<br>10 momadmitcond_swf___10 Surgical site infection<br>11 momadmitcond_swf___11 Puerperal sepsis<br>12 momadmitcond_swf___12 Other infection<br>13 momadmitcond_swf___13 Diarrhea<br>14 momadmitcond_swf___14 Vomiting<br>15 momadmitcond_swf___15 PPD/psychosis<br>16 momadmitcond_swf___16 Needed another surgery<br>17 momadmitcond_swf___17 Needed blood transfusion<br>18 momadmitcond_swf___18 Needed oxygen<br>98 momadmitcond_swf___98 Other (specify)<br>99 momadmitcond_swf___99 None
	240	[momadmitcondother_swf]<br>Show the field ONLY if:<br>[momadmitcond_swf(98)] = '1'	Other condition:	text
	241	[momseek_swf]<br>Show the field ONLY if:<br>[momalive_swf] = '1'	Did she seek care at a facility at any time after being home post-discharge that DID NOT result in an admission?	yesno<br>1 Yes<br>0 No
	242	[momseekdate_swf]<br>Show the field ONLY if:<br>[momseek_swf] = '1'	Date of first care seeking visit:	text (date_dmy)
	243	[momseekpathway_swf]<br>Show the field ONLY if:<br>[momseek_swf] = '1'	What was the care-seeking pathway for this visit?	checkbox<br>1 momseekpathway_swf___1 Routine PNC visit identified problem<br>2 momseekpathway_swf___2 Self referral<br>3 momseekpathway_swf___3 Both self-referral and PNC visit resulted in care seeking<br>98 momseekpathway_swf___98 Other
	244	[momseekpathwayother_swf]<br>Show the field ONLY if:<br>[momseekpathway_swf(98)] = '1'	Other pathway:	text

	245	[momseeksymp_swf]<br><br>Show the field ONLY if:<br>[momseek_swf] = '1'	What were the symptoms she experienced during this illness (check all that apply)?	checkbox<br>1 momseeksymp_swf___1 Heavy vaginal bleeding<br>2 momseeksymp_swf___2 Bleeding from nose/ eyes/ ears<br>3 momseeksymp_swf___3 Petechiae (small red dots and bruises across the skin)<br>4 momseeksymp_swf___4 Abnormal tiredness<br>5 momseeksymp_swf___5 Convulsions<br>6 momseeksymp_swf___6 Shortness of breath<br>7 momseeksymp_swf___7 Changes in vision<br>8 momseeksymp_swf___8 Severe headache < 24 hrs<br>9 momseeksymp_swf___9 Severe headache >24 hrs<br>10 momseeksymp_swf___10 Abdominal pain<br>11 momseeksymp_swf___11 Abdominal tenderness when touched<br>12 momseeksymp_swf___12 Foul smelling vaginal discharge<br>13 momseeksymp_swf___13 Fever/ body hotness < 7days<br>14 momseeksymp_swf___14 Fever/ body hotness >7days<br>15 momseeksymp_swf___15 Diarrhea < 14 days<br>16 momseeksymp_swf___16 Diarrhea >14 days<br>17 momseeksymp_swf___17 Vomiting everything<br>18 momseeksymp_swf___18 Cough < 14 days<br>19 momseeksymp_swf___19 Cough >14 days<br>20 momseeksymp_swf___20 Yellow eyes<br>21 momseeksymp_swf___21 Loss of consciousness<br>98 momseeksymp_swf___98 Other (specify)<br>99 momseeksymp_swf___99 None
	246	[momseeksympother_swf]<br><br>Show the field ONLY if:<br>[momseeksymp_swf(98)] = '1'	Other symptom:	text

	247	[momseekcond_swf]<br>Show the field ONLY if:<br>[momseek_swf] = '1'	Was she told she had any of the following conditions during this visit (check all that apply)?	checkbox<br>1 momseekcond_swf___1 Pre-eclampsia<br>2 momseekcond_swf___2 Eclampsia<br>3 momseekcond_swf___3 Diabetes<br>4 momseekcond_swf___4 Fistula<br>5 momseekcond_swf___5 HIV<br>6 momseekcond_swf___6 Malaria<br>7 momseekcond_swf___7 Retained placenta<br>8 momseekcond_swf___8 Heavy bleeding<br>9 momseekcond_swf___9 Anemia<br>10 momseekcond_swf___10 Surgical site infection<br>11 momseekcond_swf___11 Puerperal sepsis<br>12 momseekcond_swf___12 Other infection<br>13 momseekcond_swf___13 Diarrhea<br>14 momseekcond_swf___14 Vomiting<br>15 momseekcond_swf___15 PPD/psychosis<br>16 momseekcond_swf___16 Needed another surgery<br>17 momseekcond_swf___17 Needed blood transfusion<br>18 momseekcond_swf___18 Needed oxygen<br>98 momseekcond_swf___98 Other (specify)<br>99 momseekcond_swf___99 None
	248	[momseekcondother_swf]<br>Show the field ONLY if:<br>[momseekcond_swf(98)] = '1'	Other condition:	text
	249	[pnc_mom_swf]<br>Show the field ONLY if:<br>[dischstat_mat] != '3'	Section Header: *PNC Visits*<br>How many PNC visits were conducted in the first 6 weeks after delivery to assess the condition of the mother (NOT for the baby)?	dropdown<br>0 0<br>1 1<br>2 2<br>3 3<br>4 >3
	250	[mompnc1_swf]<br>Show the field ONLY if:<br>[pnc_mom_swf] > 0	How many days after delivery did the first visit take place?	text (integer, Min: 1, Max: 42)
	251	[mompnc2_swf]<br>Show the field ONLY if:<br>[pnc_mom_swf] > 1	How many days after delivery did the second visit take place?	text (integer, Min: 1, Max: 42)
	252	[mompnc3_swf]<br>Show the field ONLY if:<br>[pnc_mom_swf] > 2	How many days after delivery did the third visit take place?	text (integer, Min: 1, Max: 42)
	253	[comment_swf]	Comment	notes
	254	[numbabyinstr_swf]	This woman delivered [numbabe_del] baby (babies). Please complete [numbabe_del] six-week follow-up neonatal instances.	descriptive
	255	[six_week_follow_up_maternal_complete]	Section Header: *Form Status*<br>Complete?	dropdown<br>0 Incomplete<br>1 Unverified<br>2 Complete

Instrument: **11. Six Week Follow Up Neonatal** (six_week_follow_up_neonatal)

	256	[babenum_swf]	Baby #[current-instance] of [numbabe_del]	descriptive

	257	[babedied_swf]<br>Show the field ONLY if:<br>[dispbb_neo][current-instance] = '3' or [admitdisposition_neo][current-instance] = '1'	This baby died in hospital. Please mark this form as "Complete", and continue to the next baby if applicable.	descriptive
	258	[babephysical_swf]<br>Show the field ONLY if:<br>[dispbb_neo][current-instance] != '3' and [admitdisposition_neo][current-instance] != '1'	Check this box if this is a physical (in-person) follow-up	checkbox<br>1 babephysical_swf___1
	259	[babealive_swf]<br>Show the field ONLY if:<br>[dispbb_neo][current-instance] != '3' and [admitdisposition_neo][current-instance] != '1'	Section Header: *Vital Status*<br>Is the newborn alive at the time of follow-up interview?	yesno<br>1 Yes<br>0 No
	260	[babedeathdate_swf]<br>Show the field ONLY if:<br>[babealive_swf] = '0'	On what date did the baby die?	text (date_dmy)
	261	[babedeathplace_swf]<br>Show the field ONLY if:<br>[babealive_swf] = '0'	Where did the baby die?	dropdown<br>1 Home<br>2 Hospital<br>3 on the way to hospital<br>98 Other
	262	[babedeathplaceother_swf]<br>Show the field ONLY if:<br>[babedeathplace_swf] = '98'	Specify other	text
	263	[babeadmit_swf]<br>Show the field ONLY if:<br>[babealive_swf] = '1'	Section Header: *Health seeking/readmissions*<br>Was the newborn admitted for one or more nights at a facility for any reason after being home post-delivery?	yesno<br>1 Yes<br>0 No
	264	[numbabeadmit_swf]<br>Show the field ONLY if:<br>[babeadmit_swf] = '1'	How many times was the baby admitted in the first 6 weeks after birth	dropdown<br>1 1<br>2 2<br>3 3<br>4 >3
	265	[babeadmitage_swf]<br>Show the field ONLY if:<br>[babeadmit_swf] = '1'	How many days old was the baby during the first admission?	text (integer, Min: 1, Max: 42)
	266	[babenightsadm_swf]<br>Show the field ONLY if:<br>[babeadmit_swf] = '1'	For how many nights were they admitted?	text (integer, Min: 1)
	267	[babeadmitpathway_swf]<br>Show the field ONLY if:<br>[babeadmit_swf] = '1'	What was the care-seeking pathway to this admission?	checkbox<br>1 babeadmitpathway_swf___1 Routine well-baby visit identified problem<br>2 babeadmitpathway_swf___2 Self referral due to parental concern<br>3 babeadmitpathway_swf___3 Both self-referral and well-baby visit resulted in care seeking<br>98 babeadmitpathway_swf___98 Other

	268	[babeadmitpathwayother_swf]<br><br>Show the field ONLY if:<br>[babeadmitpathway_swf(98)] = '1'	Other pathway:	text
	269	[babeadmitsymp_swf]<br><br>Show the field ONLY if:<br>[babeadmit_swf] = '1'	What were the symptoms the child experienced during this illness (check all that apply)?	checkbox<br>1 babeadmitsymp_swf___1 Skin pustules<br>2 babeadmitsymp_swf___2 Respiratory distress<br>3 babeadmitsymp_swf___3 Watery stool<br>4 babeadmitsymp_swf___4 Fever/hotness of body<br>5 babeadmitsymp_swf___5 Bilious vomit<br>6 babeadmitsymp_swf___6 projectile vomit<br>7 babeadmitsymp_swf___7 Not waking up to feed or abnormally sleepy<br>8 babeadmitsymp_swf___8 Swelling of both feet<br>9 babeadmitsymp_swf___9 Changes in urine color<br>10 babeadmitsymp_swf___10 Making less urine than usual<br>11 babeadmitsymp_swf___11 Blood in stool<br>12 babeadmitsymp_swf___12 Seizure/convulsions<br>13 babeadmitsymp_swf___13 Coma<br>14 babeadmitsymp_swf___14 Yellow soles<br>15 babeadmitsymp_swf___15 Difficulty wtih breastfeeding<br>16 babeadmitsymp_swf___16 Cough<br>17 babeadmitsymp_swf___17 Umbilical cord problem (pain, discharge, etc.)<br>18 babeadmitsymp_swf___18 Not gaining weight<br>98 babeadmitsymp_swf___98 Other<br>99 babeadmitsymp_swf___99 None
	270	[babeadmitsympother_swf]<br><br>Show the field ONLY if:<br>[babeadmitsymp_swf(98)] = '1 '	Other symptom:	text
	271	[transfusion_swf]<br><br>Show the field ONLY if:<br>[babeadmit_swf] = '1'	Was baby transfused during any admission post-discharge	yesno<br>1 Yes<br>0 No

	#	Variable / Field Name	Field Label	Field Attributes
	272	[babeadmitcond_swf]<br><br>Show the field ONLY if:<br>[babeadmit_swf] = '1'	Were you told the child had any of the following conditions?	checkbox<br>1 babeadmitcond_swf___1 Sepsis<br>2 babeadmitcond_swf___2 Respiratory illness<br>3 babeadmitcond_swf___3 Oncologic disease (cancer)<br>4 babeadmitcond_swf___4 Congenital abnormality<br>5 babeadmitcond_swf___5 Endocrine/ metabolic disease<br>6 babeadmitcond_swf___6 Failure to thrive<br>7 babeadmitcond_swf___7 Trauma/ injury<br>8 babeadmitcond_swf___8 HIV<br>9 babeadmitcond_swf___9 Sickle Cell<br>10 babeadmitcond_swf___10 Anemia<br>11 babeadmitcond_swf___11 Dehydration<br>98 babeadmitcond_swf___98 Other<br>99 babeadmitcond_swf___99 None
	273	[babeadmitcondother_swf]<br><br>Show the field ONLY if:<br>[babeadmitcond_swf(98)] = '1'	Other condition:	text
	274	[matsymp_babeoutcome]<br><br>Show the field ONLY if:<br>[babeadmit_swf]=1	Was the mother experiencing any signifcant illness at the time when baby was admitted?	yesno<br>1 Yes<br>0 No
	275	[babefeed_outcome]<br><br>Show the field ONLY if:<br>[babealive_swf] = '1'	Is the baby being exclusively breastfed	yesno<br>1 Yes<br>0 No
	276	[babeseek_swf]<br><br>Show the field ONLY if:<br>[babealive_swf] = '1'	Did you seek care for the newborn at a facility at any time after being home post-discharge that DID NOT result in an admission?	yesno<br>1 Yes<br>0 No
	277	[babeseekage_swf]<br><br>Show the field ONLY if:<br>[babeseek_swf] = '1'	How many days old was the baby during the first such visit?	text (integer, Min: 1, Max: 42)
	278	[babeseekpathway_swf]<br><br>Show the field ONLY if:<br>[babeseek_swf] = '1'	What was the care-seeking pathway for this visit?	checkbox<br>1 babeseekpathway_swf___1 Routine well-baby visit identified problem<br>2 babeseekpathway_swf___2 Self referral due to parental concern<br>3 babeseekpathway_swf___3 Both self-referral and well-baby visit resulted in care seeking<br>98 babeseekpathway_swf___98 Other
	279	[babeseekpathwayother_swf]<br><br>Show the field ONLY if:<br>[babeseekpathway_swf(98)] = '1'	Other pathway:	text
	280	[pnc_baby_swf]<br><br>Show the field ONLY if:<br>[dispbb_neo][current-instance] != '3' and [admitdisposition_neo][current-instance] != '1'	Section Header: *Well-baby visits (i.e. PNC visits)*<br><br>How many well-baby visits were conducted in the first 6 weeks (i.e. post-natal care visits for the baby) during which your baby was physically examined	dropdown<br>0 0<br>1 1<br>2 2<br>3 3<br>4 >3

	281	[babepnc1_swf]<br><br>Show the field ONLY if:<br>[pnc_baby_swf] > 0	How many days old was this baby at the first visit?	text (integer, Min: 1, Max: 42)
	282	[babepnc2_swf]<br><br>Show the field ONLY if:<br>[pnc_baby_swf] > 1	How many days old was this baby at the second visit?	text (integer, Min: 1, Max: 42)
	283	[babepnc3_swf]<br><br>Show the field ONLY if:<br>[pnc_baby_swf] > 2	How many days old was this baby at the third visit?	text (integer, Min: 1, Max: 42)
	284	[comment2_swf]	Comment	notes
	285	[six_week_follow_up_neonatal_complete]	Section Header: *Form Status*<br>Complete?	dropdown<br>0 Incomplete<br>1 Unverified<br>2 Complete

Instrument: **12. Six Week Follow Up Wellness Checks** (six_week_follow_up_wellness_checks)

	286	[momchecks_swf]<br><br>Show the field ONLY if:<br>[momalive_swf] = '1'	Did you know you needed to attend a routine wellness check for yourself after being sent home?	yesno<br>1 Yes<br>0 No
	287	[babechecks_swf]<br><br>Show the field ONLY if:<br>[babealive_swf][first-instance] = '1' or [babealive_swf][last-instance] = '1'	Did you know you needed to attend a routine wellness check for your baby after being sent home?	yesno<br>1 Yes<br>0 No
	288	[wellnesstimesthink_swf]	How many times do you think you need to attend a routine wellness check for you or your baby after being sent home?	radio<br>0 0<br>1 1<br>2 2<br>3 3<br>4 4<br>5 More than 4
	289	[wellnesssource_swf]<br><br>Show the field ONLY if:<br>[momchecks_swf] = '1' or [babechecks_swf] = '1'	How did you know to do this?	checkbox<br>1 wellnesssource_swf___1 Information from a healthcare worker at discharge<br>2 wellnesssource_swf___2 Previous pregnancy or experience<br>3 wellnesssource_swf___3 Guidance from a family member or friend<br>4 wellnesssource_swf___4 Date listed on the discharge form<br>98 wellnesssource_swf___98 Other
	290	[wellnesssourceother_swf]<br><br>Show the field ONLY if:<br>[wellnesssource_swf(98)] = '1'	Specify any other ways you knew to do this.	text
	291	[wellnesstimes_swf]<br><br>Show the field ONLY if:<br>[momchecks_swf] = '1' or [babechecks_swf] = '1'	How many times were you told to seek routine wellness checks?	radio<br>0 0<br>1 1<br>2 2<br>3 3<br>4 4<br>5 More than 4<br><br>Field Annotation: @HIDDEN

<table>
<tr><td>292</td><td>[wellnesswhen_swf]

Show the field ONLY if:
[momchecks_swf] = '1' or [babechecks_swf] = '1'</td><td>What did the information you received tell you about when you should attend a routine wellness check (time periods relate to days/hours after birth)?</td><td>checkbox<table>
<tr><td>1</td><td>wellnesswhen_swf___1</td><td>1 day (24 hours)</td></tr>
<tr><td>2</td><td>wellnesswhen_swf___2</td><td>3 days (48-72 hours)</td></tr>
<tr><td>3</td><td>wellnesswhen_swf___3</td><td>6 days</td></tr>
<tr><td>4</td><td>wellnesswhen_swf___4</td><td>7-14 days</td></tr>
<tr><td>5</td><td>wellnesswhen_swf___5</td><td>6 weeks</td></tr>
<tr><td>98</td><td>wellnesswhen_swf___98</td><td>Other</td></tr>
<tr><td>99</td><td>wellnesswhen_swf___99</td><td>None</td></tr>
</table></td></tr>
<tr><td>293</td><td>[wellnesswhenother_swf]

Show the field ONLY if:
[wellnesswhen_swf(98)] = '1'</td><td>Specify the other times you were told to do this.</td><td>text</td></tr>
<tr><td>294</td><td>[wellnessreasonnot_swf]</td><td>Is there any reason you may not have attended?</td><td>checkbox<table>
<tr><td>1</td><td>wellnessreasonnot_swf___1</td><td>I did not think it was necessary</td></tr>
<tr><td>2</td><td>wellnessreasonnot_swf___2</td><td>I did not know</td></tr>
<tr><td>3</td><td>wellnessreasonnot_swf___3</td><td>Cost of transport</td></tr>
<tr><td>4</td><td>wellnessreasonnot_swf___4</td><td>Distance to nearest facility</td></tr>
<tr><td>5</td><td>wellnessreasonnot_swf___5</td><td>Bad weather or roads</td></tr>
<tr><td>6</td><td>wellnessreasonnot_swf___6</td><td>No one to look after other children (child care)</td></tr>
<tr><td>7</td><td>wellnessreasonnot_swf___7</td><td>Previous bad experience with the health workers</td></tr>
<tr><td>8</td><td>wellnessreasonnot_swf___8</td><td>Fears or insecurities</td></tr>
<tr><td>9</td><td>wellnessreasonnot_swf___9</td><td>Decision is made by my husband/partner or someone else in the family</td></tr>
<tr><td>10</td><td>wellnessreasonnot_swf___10</td><td>Cost of care at the facility</td></tr>
<tr><td>98</td><td>wellnessreasonnot_swf___98</td><td>Other</td></tr>
<tr><td>99</td><td>wellnessreasonnot_swf___99</td><td>None</td></tr>
</table></td></tr>
<tr><td>295</td><td>[wellnessreasonnotother_swf]

Show the field ONLY if:
[wellnessreasonnot_swf(98)] = '1'</td><td>Specify other reason you may not have attended.</td><td>text</td></tr>
</table>

	#	Variable	Field Label	Field Attributes
	296	[wellnessdiscuss_swf]	Were any of the following discussed with you by a healthcare worker regarding seeking care for you and your baby?	checkbox<br>1 | wellnessdiscuss_swf___1 | Seek care for illness or complications<br>2 | wellnessdiscuss_swf___2 | Attend family planning at 6 weeks<br>3 | wellnessdiscuss_swf___3 | Attend vaccination visit at 6 weeks<br>4 | wellnessdiscuss_swf___4 | Attend routine wellness checks throughout the 6 week postnatal period<br>5 | wellnessdiscuss_swf___5 | Healthcare workers would complete home visits for routine wellness checks<br>99 | wellnessdiscuss_swf___99 | None
	297	[six_week_follow_up_wellness_checks_complete]	Section Header: *Form Status*<br>Complete?	dropdown<br>0 | Incomplete<br>1 | Unverified<br>2 | Complete

Instrument: **Verbal Autopsy - Mother** (verbal_autopsy_mother)

	#	Variable	Field Label	Field Attributes
	298	[confirm_studyid_va_m]	Confirm Study ID	text
	299	[date_va_m]	Date of verbal autopsy:	text (date_dmy)
	300	[id10007_m]	Section Header: *Information on the respondent and background about interview*<br>What is the sex of the VA respondent?	radio<br>1 | Female<br>2 | Male<br>3 | Ambiguous/Intersex
	301	[id10008_m]	What is your/the respondent's relationship to the deceased?	dropdown<br>1 | Parent<br>2 | Child<br>3 | Other family member<br>4 | Friend<br>5 | Spouse<br>6 | Health worker<br>7 | Public official<br>8 | Another relationship<br>88 | Refused to answer
	302	[id10009_m]	Did you/the respondent live with the deceased in the period leading to her death?	radio<br>1 | Yes<br>0 | No<br>99 | Doesn't know<br>88 | Refused to answer
	303	[id10013_m]	Did the respondent give consent?	radio<br>1 | Yes<br>0 | No
	304	[ageatdeath]	Mother's age at death (in years):	calc<br>Calculation: if([dischstat_mat] = '3' or [momalive_swf] = '0', if([dobknown_adm_v2]='1', (rounddown(datediff([momdeathdate_swf], [dob_adm_v2], 'y'))), [dob_estage_adm_v2]), '')

	305	[id10487_m]	Section Header: *You will now be filling in the questionnaire for an ADULT.*<br>In the two weeks before death, did she live with or visit someone who had any COVID-19 symptoms or a positive COVID-19 test?	radio<br>1 Yes<br>0 No<br>99 Doesn't know<br>88 Refused to answer
	306	[id10488_m]	In the two weeks before death, did she travel to an area where COVID-19 is known to be present?<br>*Based on self-report of the respondent. If there is doubt, note the location in the narrative and check with the respective supervisor.*	radio<br>1 Yes<br>0 No<br>99 Doesn't know<br>88 Refused to answer
	307	[momadmit_va]<br>Show the field ONLY if:<br>[dischstat_mat] != '3'	Section Header: *Health seeking/readmissions*<br>Was the mother admitted for one or more nights at a facility for any reason after being home post-delivery?	yesno<br>1 Yes<br>0 No
	308	[nummomadmit_va]<br>Show the field ONLY if:<br>[momadmit_va] = '1'	How many times was the mother admitted in the first 6 weeks after delivery?	dropdown<br>1 1<br>2 2<br>3 3<br>4 >3
	309	[momadmitdate_va]<br>Show the field ONLY if:<br>[momadmit_va] = '1'	Date of first admission	text (date_dmy)
	310	[momnightsadm_va]<br>Show the field ONLY if:<br>[momadmit_va] = '1'	For how many nights was she admitted?	text (integer, Min: 1)
	311	[momadmitpathway_va]<br>Show the field ONLY if:<br>[momadmit_va] = '1'	What was the care-seeking pathway to the first admission?	checkbox<br>1 momadmitpathway_va___1 Routine PNC visit identified problem<br>2 momadmitpathway_va___2 Self referral<br>3 momadmitpathway_va___3 Both self-referral and PNC visit resulted in care seeking<br>98 momadmitpathway_va___98 Other
	312	[momadmitpathwayother_va]<br>Show the field ONLY if:<br>[momadmitpathway_va(98)] = '1'	Other pathway:	text

	313	[momadmitsymp_va]<br><br>Show the field ONLY if:<br>[momadmit_va] = '1'	What were the symptoms she experienced during this illness? (check all that apply)	checkbox

1	momadmitsymp_va___1	Heavy vaginal bleeding
2	momadmitsymp_va___2	Bleeding from nose/ eyes/ ears
3	momadmitsymp_va___3	Petechiae (small red dots and bruises across the skin)
4	momadmitsymp_va___4	Abnormal tiredness
5	momadmitsymp_va___5	Convulsions
6	momadmitsymp_va___6	Shortness of breath
7	momadmitsymp_va___7	Changes in vision
8	momadmitsymp_va___8	Severe headache < 24 hrs
9	momadmitsymp_va___9	Severe headache >24 hrs
10	momadmitsymp_va___10	Abdominal pain
11	momadmitsymp_va___11	Abdominal tenderness when touched
12	momadmitsymp_va___12	Foul smelling vaginal discharge
13	momadmitsymp_va___13	Fever/ body hotness < 7days
14	momadmitsymp_va___14	Fever/ body hotness >7days
15	momadmitsymp_va___15	Diarrhea < 14 days
16	momadmitsymp_va___16	Diarrhea >14 days
17	momadmitsymp_va___17	Vomiting everything
18	momadmitsymp_va___18	Cough < 14 days
19	momadmitsymp_va___19	Cough >14 days
20	momadmitsymp_va___20	Yellow eyes
21	momadmitsymp_va___21	Loss of consciousness
98	momadmitsymp_va___98	Other (specify)
99	momadmitsymp_va___99	None

	314	[momadmitsympother_va]<br><br>Show the field ONLY if:<br>[momadmitsymp_va(98)] = '1'	Other symptom	text

	315	[momadmitcond_va]<br><br>Show the field ONLY if:<br>[momadmit_va] = '1'	Was she told she had any of the following conditions during this visit? (check all that apply)	checkbox<br>1 | momadmitcond_va___1 | Pre-eclampsia<br>2 | momadmitcond_va___2 | Eclampsia<br>3 | momadmitcond_va___3 | Diabetes<br>4 | momadmitcond_va___4 | Fistula<br>5 | momadmitcond_va___5 | HIV related illness<br>6 | momadmitcond_va___6 | Malaria<br>7 | momadmitcond_va___7 | Retained placenta<br>8 | momadmitcond_va___8 | Heavy bleeding<br>9 | momadmitcond_va___9 | Anemia<br>10 | momadmitcond_va___10 | Surgical site infection<br>11 | momadmitcond_va___11 | Puerperal sepsis<br>12 | momadmitcond_va___12 | Other infection<br>13 | momadmitcond_va___13 | Diarrhea<br>14 | momadmitcond_va___14 | Vomiting<br>15 | momadmitcond_va___15 | PPD/psychosis<br>16 | momadmitcond_va___16 | Needed another surgery<br>17 | momadmitcond_va___17 | Needed blood transfusion<br>18 | momadmitcond_va___18 | Needed oxygen<br>98 | momadmitcond_va___98 | Other (specify)<br>99 | momadmitcond_va___99 | None
	316	[momadmitcondother_va]<br><br>Show the field ONLY if:<br>[momadmitcond_va(98)] = '1'	Other condition:	text
	317	[momseek_va]<br><br>Show the field ONLY if:<br>[dischstat_mat] != '3'	Did she seek care at a facility at any time after being home post-delivery that DID NOT result in an admission?	yesno<br>1 | Yes<br>0 | No
	318	[momseekdate_va]<br><br>Show the field ONLY if:<br>[momseek_va] = '1'	Date of first care seeking visit:	text (date_dmy)
	319	[momseekpathway_va]<br><br>Show the field ONLY if:<br>[momseek_va] = '1'	What was the care-seeking pathway for this visit?	checkbox<br>1 | momseekpathway_va___1 | Routine PNC visit identified problem<br>2 | momseekpathway_va___2 | Self referral<br>3 | momseekpathway_va___3 | Both self-referral and PNC visit resulted in care seeking<br>98 | momseekpathway_va___98 | Other
	320	[momseekpathwayother_va]<br><br>Show the field ONLY if:<br>[momseekpathway_va(98)] = ' 1'	Other pathway:	text

	321	[momseeksymp_va]<br><br>Show the field ONLY if:<br>[momseek_va] = '1'	What were the symptoms she experienced during this illness (check all that apply)?	checkbox<br>1 momseeksymp_va___1 Heavy vaginal bleeding<br>2 momseeksymp_va___2 Bleeding from nose/ eyes/ ears<br>3 momseeksymp_va___3 Petechiae (small red dots and bruises across the skin)<br>4 momseeksymp_va___4 Abnormal tiredness<br>5 momseeksymp_va___5 Convulsions<br>6 momseeksymp_va___6 Shortness of breath<br>7 momseeksymp_va___7 Changes in vision<br>8 momseeksymp_va___8 Severe headache < 24 hrs<br>9 momseeksymp_va___9 Severe headache >24 hrs<br>10 momseeksymp_va___10 Abdominal pain<br>11 momseeksymp_va___11 Abdominal tenderness when touched<br>12 momseeksymp_va___12 Foul smelling vaginal discharge<br>13 momseeksymp_va___13 Fever/ body hotness < 7days<br>14 momseeksymp_va___14 Fever/ body hotness >7days<br>15 momseeksymp_va___15 Diarrhea < 14 days<br>16 momseeksymp_va___16 Diarrhea >14 days<br>17 momseeksymp_va___17 Vomiting everything<br>18 momseeksymp_va___18 Cough < 14 days<br>19 momseeksymp_va___19 Cough >14 days<br>20 momseeksymp_va___20 Yellow eyes<br>21 momseeksymp_va___21 Loss of consciousness<br>98 momseeksymp_va___98 Other (specify)<br>99 momseeksymp_va___99 None
	322	[momseeksympother_va]<br><br>Show the field ONLY if:<br>[momseeksymp_va(98)] = '1'	Other symptom:	text

	323	[momseekcond_va]<br>Show the field ONLY if: [momseek_va] = '1'	Was she told she had any of the following conditions during this visit (check all that apply)?	checkbox<br>1 momseekcond_va___1 Pre-eclampsia<br>2 momseekcond_va___2 Eclampsia<br>3 momseekcond_va___3 Diabetes<br>4 momseekcond_va___4 Fistula<br>5 momseekcond_va___5 HIV<br>6 momseekcond_va___6 Malaria<br>7 momseekcond_va___7 Retained placenta<br>8 momseekcond_va___8 Heavy bleeding<br>9 momseekcond_va___9 Anemia<br>10 momseekcond_va___10 Surgical site infection<br>11 momseekcond_va___11 Puerperal sepsis<br>12 momseekcond_va___12 Other infection<br>13 momseekcond_va___13 Diarrhea<br>14 momseekcond_va___14 Vomiting<br>15 momseekcond_va___15 PPD/psychosis<br>16 momseekcond_va___16 Needed another surgery<br>17 momseekcond_va___17 Needed blood transfusion<br>18 momseekcond_va___18 Needed oxygen<br>98 momseekcond_va___98 Other (specify)<br>99 momseekcond_va___99 None
	324	[momseekcondother_va]<br>Show the field ONLY if: [momseekcond_va(98)] = '1'	Other condition:	text
	325	[id10077_m]	Section Header: *History of injuries/accidents*<br>Did she suffer from any injury or accident that led to her death?	radio<br>1 Yes<br>0 No<br>99 Doesn't know<br>88 Refused to answer
	326	[id10079_m]<br>Show the field ONLY if: [id10077_m]='1'	Section Header: *Injuries and accidents detail*<br>Was it a road traffic accident?	radio<br>1 Yes<br>0 No<br>99 Doesn't know<br>88 Refused to answer
	327	[id10080_m]<br>Show the field ONLY if: [id10079_m] = '1'	What was her role in the road traffic accident?	radio<br>1 Pedestrian<br>2 Driver or passenger in car or light vehicle<br>3 Driver or passenger in bus or heavy vehicle<br>4 Driver or passenger on a motorcycle<br>5 Driver or passenger on a pedal cycle<br>6 Other<br>99 Doesn't know<br>88 Refused to answer

328	[id10081_m]<br><br>Show the field ONLY if:<br>[id10079_m] = '1'	What was the counterpart that was hit during the road traffic accident?	radio	1 Pedestrian<br>2 Stationary object<br>3 Car or light vehicle<br>4 Bus or heavy vehicle<br>5 Motorcycle<br>6 Pedal cycle<br>7 Other<br>99 Doesn't know<br>88 Refused to answer
329	[id10082_m]<br><br>Show the field ONLY if:<br>[id10079_m] = '0' or [id10079_m] = '99' or [id10079_m] = '88'	Was she injured in a non-road transport accident?	radio	1 Yes<br>0 No<br>99 Doesn't know<br>88 Refused to answer
330	[id10083_m]<br><br>Show the field ONLY if:<br>[id10079_m] = '0' or [id10079_m] = '99' or [id10079_m] = '88'	Was she injured in a fall?<br>*This includes accidents and cases where it is unknown if it was an accident or whether there was intentional violence.*	radio	1 Yes<br>0 No<br>99 Doesn't know<br>88 Refused to answer
331	[id10084_m]<br><br>Show the field ONLY if:<br>[id10079_m] = '0' or [id10079_m] = '99' or [id10079_m] = '88'	Was there any poisoning?<br>*This includes accidents and cases where it is unknown if it was an accident or whether there was intentional violence.*	radio	1 Yes<br>0 No<br>99 Doesn't know<br>88 Refused to answer
332	[id10085_m]<br><br>Show the field ONLY if:<br>[id10079_m] = '0' or [id10079_m] = '99' or [id10079_m] = '88'	Did she die of drowning?<br>*This includes accidents and cases where it is unknown if it was an accident or whether there was intentional violence.*	radio	1 Yes<br>0 No<br>99 Doesn't know<br>88 Refused to answer
333	[id10086_m]<br><br>Show the field ONLY if:<br>[id10079_m] = '0' or [id10079_m] = '99' or [id10079_m] = '88'	Was she injured by a bite or sting by venomous animal?<br>*This includes accidents and cases where it is unknown if it was an accident or whether there was intentional violence.*	radio	1 Yes<br>0 No<br>99 Doesn't know<br>88 Refused to answer
334	[id10087_m]<br><br>Show the field ONLY if:<br>[id10086_m] = '0' or [id10086_m] = '99' or [id10086_m] = '88'	Was she injured by an animal or insect (non-venomous)?	radio	1 Yes<br>0 No<br>99 Doesn't know<br>88 Refused to answer
335	[id10088_m]<br><br>Show the field ONLY if:<br>[id10086_m] = '1' or [id10087_m] = '1'	What was the animal/insect?	radio	1 Dog<br>2 Snake<br>3 Insect or scorpion<br>4 Other<br>99 Doesn't know<br>88 Refused to answer

	336	[id10089_m]<br>Show the field ONLY if: [id10079_m] = '0' or [id10079_m] = '99' or [id10079_m] = '88'	Was she injured by burns/fire?	radio<br>1 Yes<br>0 No<br>99 Doesn't know<br>88 Refused to answer
	337	[id10090_m]<br>Show the field ONLY if: [id10079_m] = '0' or [id10079_m] = '99' or [id10079_m] = '88'	Was she subject to violence (homicide, abuse)?	radio<br>1 Yes<br>0 No<br>99 Doesn't know<br>88 Refused to answer
	338	[id10091_m]<br>Show the field ONLY if: [id10079_m] = '0' or [id10079_m] = '99' or [id10079_m] = '88'	Was she injured by a firearm?	radio<br>1 Yes<br>0 No<br>99 Doesn't know<br>88 Refused to answer
	339	[id10092_m]<br>Show the field ONLY if: [id10079_m] = '0' or [id10079_m] = '99' or [id10079_m] = '88'	Was she stabbed, cut or pierced?	radio<br>1 Yes<br>0 No<br>99 Doesn't know<br>88 Refused to answer
	340	[id10093_m]<br>Show the field ONLY if: [id10079_m] = '0' or [id10079_m] = '99' or [id10079_m] = '88'	Was she strangled?	radio<br>1 Yes<br>0 No<br>99 Doesn't know<br>88 Refused to answer
	341	[id10094_m]<br>Show the field ONLY if: [id10079_m] = '0' or [id10079_m] = '99' or [id10079_m] = '88'	Was she injured by a blunt force?	radio<br>1 Yes<br>0 No<br>99 Doesn't know<br>88 Refused to answer
	342	[id10095_m]<br>Show the field ONLY if: [id10079_m] = '0' or [id10079_m] = '99' or [id10079_m] = '88'	Was she injured by a force of nature?	radio<br>1 Yes<br>0 No<br>99 Doesn't know<br>88 Refused to answer
	343	[id10096_m]<br>Show the field ONLY if: [id10079_m] = '0' or [id10079_m] = '99' or [id10079_m] = '88'	Was it electrocution?	radio<br>1 Yes<br>0 No<br>99 Doesn't know<br>88 Refused to answer
	344	[id10097_m]<br>Show the field ONLY if: [id10079_m] = '0' or [id10079_m] = '99' or [id10079_m] = '88'	Did she encounter any other injury?	radio<br>1 Yes<br>0 No<br>99 Doesn't know<br>88 Refused to answer

	345	[id10098_m]<br>Show the field ONLY if: [id10079_m] = '0' or [id10079_m] = '99' or [id10079_m] = '88'	Was the injury accidental?	radio<br>1 Yes<br>0 No<br>99 Doesn't know<br>88 Refused to answer
	346	[id10099_m]<br>Show the field ONLY if: [id10098_m] = '0' or [id10098_m] = '99' or [id10098_m] = '88'	Was the injury self-inflicted?	radio<br>1 Yes<br>0 No<br>99 Doesn't know<br>88 Refused to answer
	347	[id10100_m]<br>Show the field ONLY if: ([id10098_m] = '0' or [id10098_m] = '99' or [id10098_m] = '88') and [id10099_m] = '0' or [id10099_m] = '99' or [id10099_m] = '88'	Was the injury intentionally inflicted by someone else?	radio<br>1 Yes<br>0 No<br>99 Doesn't know<br>88 Refused to answer
	348	[id10120_m]	Section Header: *Duration of Illness*<br>For how long was she ill before death? (in days)	text (integer, Min: 0, Max: 300)
	349	[id10123_m]	Did she die suddenly?<br>*Suddenly means died unexpectedly within 24 hours of being in regular health.*	radio<br>1 Yes<br>0 No<br>99 Doesn't know<br>88 Refused to answer
	350	[id10125_m]	Section Header: *Medical History Associated With Final Illness Remind the respondent that we are asking for the diagnosis assessed by a doctor, health worker, or other health professional during the final illness.*<br>Was there any diagnosis by a health professional of tuberculosis?	radio<br>1 Yes<br>0 No<br>99 Doesn't know<br>88 Refused to answer
	351	[id10126_m]	Was an HIV test ever positive?	radio<br>1 Yes<br>0 No<br>99 Doesn't know<br>88 Refused to answer
	352	[id10127_m]	Was there any diagnosis by a health professional of AIDS?	radio<br>1 Yes<br>0 No<br>99 Doesn't know<br>88 Refused to answer
	353	[id10128_m]	Did she have a recent positive test by a health professional for malaria?	radio<br>1 Yes<br>0 No<br>99 Doesn't know<br>88 Refused to answer
	354	[id10129_m]	Did she have a recent negative test by a health professional for malaria?	radio<br>1 Yes<br>0 No<br>99 Doesn't know<br>88 Refused to answer

	355	[id10130_m]	Was there any diagnosis by a health professional of dengue fever?	radio<br>1 Yes<br>0 No<br>99 Doesn't know<br>88 Refused to answer
	356	[id10131_m]	Was there any diagnosis by a health professional of measles?	radio<br>1 Yes<br>0 No<br>99 Doesn't know<br>88 Refused to answer
	357	[id10132_m]	Was there any diagnosis by a health professional of high blood pressure?	radio<br>1 Yes<br>0 No<br>99 Doesn't know<br>88 Refused to answer
	358	[id10133_m]	Was there any diagnosis by a health professional of heart disease?	radio<br>1 Yes<br>0 No<br>99 Doesn't know<br>88 Refused to answer
	359	[id10134_m]	Was there any diagnosis by a health professional of diabetes?	radio<br>1 Yes<br>0 No<br>99 Doesn't know<br>88 Refused to answer
	360	[id10135_m]	Was there any diagnosis by a health professional of asthma?	radio<br>1 Yes<br>0 No<br>99 Doesn't know<br>88 Refused to answer
	361	[id10136_m]	Was there any diagnosis by a health professional of epilepsy?	radio<br>1 Yes<br>0 No<br>99 Doesn't know<br>88 Refused to answer
	362	[id10137_m]	Was there any diagnosis by a health professional of cancer?	radio<br>1 Yes<br>0 No<br>99 Doesn't know<br>88 Refused to answer
	363	[id10138_m]	Was there any diagnosis by a health professional of Chronic Obstructive Pulmonary Disease (COPD)?	radio<br>1 Yes<br>0 No<br>99 Doesn't know<br>88 Refused to answer

	364	[id10139_m]	Was there any diagnosis by a health professional of dementia?	radio<br>1 Yes<br>0 No<br>99 Doesn't know<br>88 Refused to answer
	365	[id10140_m]	Was there any diagnosis by a health professional of depression?	radio<br>1 Yes<br>0 No<br>99 Doesn't know<br>88 Refused to answer
	366	[id10141_m]	Was there any diagnosis by a health professional of stroke?	radio<br>1 Yes<br>0 No<br>99 Doesn't know<br>88 Refused to answer
	367	[id10142_m]	Was there any diagnosis by a health professional of sickle cell disease?	radio<br>1 Yes<br>0 No<br>99 Doesn't know<br>88 Refused to answer
	368	[id10143_m]	Was there any diagnosis by a health professional of kidney disease?	radio<br>1 Yes<br>0 No<br>99 Doesn't know<br>88 Refused to answer
	369	[id10144_m]	Was there any diagnosis by a health professional of liver disease?	radio<br>1 Yes<br>0 No<br>99 Doesn't know<br>88 Refused to answer
	370	[id10482_m]	Was there any diagnosis by a health professional of COVID-19?	radio<br>1 Yes<br>0 No<br>99 Doesn't know<br>88 Refused to answer
	371	[id10483_m]	Did she have a recent test by a health professional for COVID-19?	radio<br>1 Yes<br>0 No<br>99 Doesn't know<br>88 Refused to answer
	372	[id10484_m]<br>Show the field ONLY if: [id10483_m]='1'	What was the result?	radio<br>1 Positive<br>2 Negative<br>3 Unclear<br>99 Don't know<br>88 Refused to answer

	373	[id10147_m]	Section Header: *General Signs and Symptoms Associated with Final Illness*<br>Did she have a fever?	radio<br>1 Yes<br>0 No<br>99 Doesn't know<br>88 Refused to answer
	374	[id10148_m]<br>Show the field ONLY if: [id10147_m]='1'	How long did the fever last? (in days)	text (number)
	375	[id10149_m]<br>Show the field ONLY if: [id10147_m]='1'	Did the fever continue until death?	radio<br>1 Yes<br>0 No<br>99 Doesn't know<br>88 Refused to answer
	376	[id10150_m]<br>Show the field ONLY if: [id10147_m]='1'	How severe was the fever?	radio<br>1 Mild<br>2 Moderate<br>3 Severe<br>99 Doesn't know<br>88 Refused to answer
	377	[id10151_m]<br>Show the field ONLY if: [id10147_m]='1'	What was the pattern of the fever?	radio<br>1 Continuous<br>2 On and off<br>3 Only at night<br>99 Doesn't know<br>88 Refused to answer
	378	[id10152_m]	Did she have night sweats?	radio<br>1 Yes<br>0 No<br>99 Doesn't know<br>88 Refused to answer
	379	[id10153_m]	Did she have a cough?	radio<br>1 Yes<br>0 No<br>99 Doesn't know<br>88 Refused to answer
	380	[id10154_m]<br>Show the field ONLY if: [id10153_m]='1'	For how long did she have a cough?	text (number)
	381	[id10155_m]<br>Show the field ONLY if: [id10153_m]='1'	Was the cough productive, with sputum?	radio<br>1 Yes<br>0 No<br>99 Doesn't know<br>88 Refused to answer
	382	[id10156_m]<br>Show the field ONLY if: [id10153_m]='1'	Was the cough very severe?	radio<br>1 Yes<br>0 No<br>99 Doesn't know<br>88 Refused to answer

	383	[id10157_m]<br>Show the field ONLY if: [id10153_m]='1'	Did she cough up blood?	radio<br>1 Yes<br>0 No<br>99 Doesn't know<br>88 Refused to answer
	384	[id10159_m]	Did she have any difficulty breathing?	radio<br>1 Yes<br>0 No<br>99 Doesn't know<br>88 Refused to answer
	385	[id10161_m]<br>Show the field ONLY if: [id10159_m]='1'	For how many days did the difficulty breathing last?	text (number)
	386	[id10165_m]<br>Show the field ONLY if: [id10159_m]='1'	Was the difficulty continuous or on and off?	radio<br>1 Continuous<br>2 On and off<br>99 Doesn't know<br>88 Refused to answer
	387	[id10166_m]	During the illness that led to death, did she have fast breathing?	radio<br>1 Yes<br>0 No<br>99 Doesn't know<br>88 Refused to answer
	388	[id10167_m]<br>Show the field ONLY if: [id10166_m]='1'	For how many days did the fast breathing last?	text (number)
	389	[id10168_m]	Did she have breathlessness?	radio<br>1 Yes<br>0 No<br>99 Doesn't know<br>88 Refused to answer
	390	[id10169_m]<br>Show the field ONLY if: [id10168_m]='1'	For how many days did she have breathlessness?	text (number)
	391	[id10170_m]<br>Show the field ONLY if: [id10168_m]='1'	Was she unable to carry out daily routines due to breathlessness?	radio<br>1 Yes<br>0 No<br>99 Doesn't know<br>88 Refused to answer
	392	[id10171_m]<br>Show the field ONLY if: [id10168_m]='1'	Was she breathless while lying flat?	radio<br>1 Yes<br>0 No<br>99 Doesn't know<br>88 Refused to answer
	393	[id10173_m]	During the illness that led to death did she have wheezing?	radio<br>1 Yes<br>0 No<br>99 Doesn't know<br>88 Refused to answer

	394	[id10174_m]	Did she have chest pain?	radio<br>1 Yes<br>0 No<br>99 Doesn't know<br>88 Refused to answer
	395	[id10175_m]<br>Show the field ONLY if: [id10174_m]='1'	Was the chest pain severe?	radio<br>1 Yes<br>0 No<br>99 Doesn't know<br>88 Refused to answer
	396	[id10179_m]<br>Show the field ONLY if: [id10174_m]='1'	How long did the chest pain last?	text (number)
	397	[id10181_m]	Did she have more frequent loose or liquid stools than usual?<br>*Ask the respondent about their understanding of what is diarrhea (having more frequent loose or liquid stools than usual); if unclear, explain to the respondent what diarrhea is.*	radio<br>1 Yes<br>0 No<br>99 Doesn't know<br>88 Refused to answer
	398	[id10182_m]<br>Show the field ONLY if: [id10181_m]='1'	For how many days did she have frequent loose or liquid stools?	text (number)
	399	[id10186_m]	At any time during the final illness was there blood in the stools?	radio<br>1 Yes<br>0 No<br>99 Doesn't know<br>88 Refused to answer
	400	[id10187_m]<br>Show the field ONLY if: [id10186_m]='1'	Was there blood in the stool up until death?	radio<br>1 Yes<br>0 No<br>99 Doesn't know<br>88 Refused to answer
	401	[id10188_m]	Did she vomit?	radio<br>1 Yes<br>0 No<br>99 Doesn't know<br>88 Refused to answer
	402	[id10189_m]	To clarify: Did she vomit in the week preceding the death?	radio<br>1 Yes<br>0 No<br>99 Doesn't know<br>88 Refused to answer
	403	[id10190_m]<br>Show the field ONLY if: [id10188_m]='1' or [id10189_m]='1'	How long before death did she vomit? (in days)	text (number)
	404	[id10192_m]<br>Show the field ONLY if: [id10188_m]='1' or [id10189_m]='1'	Was the vomit black?	radio<br>1 Yes<br>0 No<br>99 Doesn't know<br>88 Refused to answer

	405	[id10193_m]	Did she have any belly (abdominal) problem?<br>*Explain to the respondent that problems could be pain, protruding abdomen or a mass.*	radio<br>1 Yes<br>0 No<br>99 Doesn't know<br>88 Refused to answer
	406	[id10194_m]	Did she have belly (abdominal) pain?	radio<br>1 Yes<br>0 No<br>99 Doesn't know<br>88 Refused to answer
	407	[id10195_m]<br>Show the field ONLY if: [id10194_m]='1'	Was the belly (abdominal) pain severe?	radio<br>1 Yes<br>0 No<br>99 Doesn't know<br>88 Refused to answer
	408	[id10197_m]<br>Show the field ONLY if: [id10194_m]='1'	For how long did she have belly (abdominal) pain? (in days)	text (number)
	409	[id10199_m]<br>Show the field ONLY if: [id10194_m]='1'	Was the pain in the upper or lower belly (abdomen)?	radio<br>1 Upper abdomen<br>2 Lower abdomen<br>3 Upper and lower abdomen<br>99 Doesn't know<br>88 Refused to answer
	410	[id10200_m]	Did she have a more than usually protruding belly (abdomen)?	radio<br>1 Yes<br>0 No<br>99 Doesn't know<br>88 Refused to answer
	411	[id10201_m]<br>Show the field ONLY if: [id10200_m]='1'	For how long before death did she have a more than usually protruding belly (abdomen)? (in days)	text (number)
	412	[id10203_m]<br>Show the field ONLY if: [id10200_m]='1'	How rapidly did she develop the protruding belly (abdomen)?	radio<br>1 Rapidly<br>2 Slowly<br>99 Doesn't know<br>88 Refused to answer
	413	[id10204_m]	Did she have any mass in the belly (abdomen)?	radio<br>1 Yes<br>0 No<br>99 Doesn't know<br>88 Refused to answer
	414	[id10205_m]<br>Show the field ONLY if: [id10204_m]='1'	For how long did she have a mass in the belly (abdomen)? (in days)	text (number)
	415	[id10207_m]	Did she have a severe headache?	radio<br>1 Yes<br>0 No<br>99 Doesn't know<br>88 Refused to answer

	416	[id10208_m]	Did she have a stiff neck during illness that led to death?	radio<br>1 Yes<br>0 No<br>99 Doesn't know<br>88 Refused to answer
	417	[id10209_m]<br>Show the field ONLY if: [id10208_m]='1'	How long before death did she have a stiff neck? (in days)	text (number)
	418	[id10210_m]	Did she have a painful neck during the illness that led to death?	radio<br>1 Yes<br>0 No<br>99 Doesn't know<br>88 Refused to answer
	419	[id10211_m]<br>Show the field ONLY if: [id10210_m]='1'	For how many days before death did she have a painful neck?	text (number)
	420	[id10212_m]	Did she have mental confusion?	radio<br>1 Yes<br>0 No<br>99 Doesn't know<br>88 Refused to answer
	421	[id10213_m]<br>Show the field ONLY if: [id10212_m]='1'	How long did she have mental confusion? (in days)	text (number)
	422	[id10214_m]	Was she unconscious during the illness that led to death?	radio<br>1 Yes<br>0 No<br>99 Doesn't know<br>88 Refused to answer
	423	[id10215_m]<br>Show the field ONLY if: [id10214_m]='1'	Was she unconscious for more than 24 hours before death?	radio<br>1 Yes<br>0 No<br>99 Doesn't know<br>88 Refused to answer
	424	[id10217_m]<br>Show the field ONLY if: [id10214_m]='1'	Did the unconsciousness start suddenly, quickly (at least within a single day)?	radio<br>1 Yes<br>0 No<br>99 Doesn't know<br>88 Refused to answer
	425	[id10218_m]<br>Show the field ONLY if: [id10214_m]='1'	Did the unconsciousness continue until death?	radio<br>1 Yes<br>0 No<br>99 Doesn't know<br>88 Refused to answer
	426	[id10219_m]	Did she have convulsions?	radio<br>1 Yes<br>0 No<br>99 Doesn't know<br>88 Refused to answer

	427	[id10221_m]<br><br>Show the field ONLY if: [id10219_m]='1'	For how many minutes did the convulsions last?<br>*The answer could be given in another unit, but for data entry use minutes. Less than 1 minute=0; 1 hour=60 minutes. Enter "99" for "don't know." Enter "88" for "refuse."*	text
	428	[id10222_m]<br><br>Show the field ONLY if: [id10219_m]='1'	Did she become unconscious immediately after the convulsion?	radio<br>1 Yes<br>0 No<br>99 Doesn't know<br>88 Refused to answer
	429	[id10223_m]	Did she have any urine problems?<br>*Explain to the respondent that urine problems refer to urinating a lot or not at all, and blood in the urine.*	radio<br>1 Yes<br>0 No<br>99 Doesn't know<br>88 Refused to answer
	430	[id10225_m]<br><br>Show the field ONLY if: [id10223_m]='1'	Did she go to urinate more often than usual?	radio<br>1 Yes<br>0 No<br>99 Doesn't know<br>88 Refused to answer
	431	[id10226_m]<br><br>Show the field ONLY if: [id10223_m]='1'	During the final illness did she ever pass blood in the urine?	radio<br>1 Yes<br>0 No<br>99 Doesn't know<br>88 Refused to answer
	432	[id10224_m]<br><br>Show the field ONLY if: [id10223_m]='1'	Did she stop urinating?	radio<br>1 Yes<br>0 No<br>99 Doesn't know<br>88 Refused to answer
	433	[id10227_m]	Did she have sores or ulcers anywhere on the body?	radio<br>1 Yes<br>0 No<br>99 Doesn't know<br>88 Refused to answer
	434	[id10228_m]<br><br>Show the field ONLY if: [id10227_m]='1'	Did she have sores?	radio<br>1 Yes<br>0 No<br>99 Doesn't know<br>88 Refused to answer
	435	[id10229_m]<br><br>Show the field ONLY if: [id10228_m]='1'	Did the sores have clear fluid or pus?	radio<br>1 Yes<br>0 No<br>99 Doesn't know<br>88 Refused to answer
	436	[id10230_m]<br><br>Show the field ONLY if: [id10227_m]='1'	Did she have an ulcer (pit) on the foot?	radio<br>1 Yes<br>0 No<br>99 Doesn't know<br>88 Refused to answer

	437	[id10231_m]<br><br>Show the field ONLY if: [id10230_m]='1'	Did the ulcer on the foot ooze pus?	radio<br>1 Yes<br>0 No<br>99 Doesn't know<br>88 Refused to answer
	438	[id10232_m]<br><br>Show the field ONLY if: [id10231_m]='1'	For how many days did the ulcer on the foot ooze pus?	text
	439	[id10233_m]	During the illness that led to death, did she have any skin rash?	radio<br>1 Yes<br>0 No<br>99 Doesn't know<br>88 Refused to answer
	440	[id10234_m]<br><br>Show the field ONLY if: [id10233_m]='1'	For how many days did she have the skin rash?<br>*The answer could be given in another unit, but for data entry use days. Less than 1 day or 24 hours = 0 days; 1 week=7 days; 1 month=30 days. Enter "99" for "don't know." Enter "88" for "refuse."*	text
	441	[id10235_m]<br><br>Show the field ONLY if: [id10233_m]='1'	Where was the rash?	checkbox<br>1 id10235_m___1 Face<br>2 id10235_m___2 Trunk or abdomen<br>3 id10235_m___3 Extremities<br>4 id10235_m___4 Everywhere
	442	[id10236_m]<br><br>Show the field ONLY if: [id10233_m]='1'	Did she have measles rash (use local term)?	radio<br>1 Yes<br>0 No<br>99 Doesn't know<br>88 Refused to answer
	443	[id10237_m]	Did she ever have shingles or herpes zoster?	radio<br>1 Yes<br>0 No<br>99 Doesn't know<br>88 Refused to answer
	444	[id10238_m]	During the illness that led to death, did her skin flake off in patches?	radio<br>1 Yes<br>0 No<br>99 Doesn't know<br>88 Refused to answer
	445	[id10241_m]	During the illness that led to death, did she bleed from anywhere?	radio<br>1 Yes<br>0 No<br>99 Doesn't know<br>88 Refused to answer
	446	[id10242_m]<br><br>Show the field ONLY if: [id10241_m]='1'	Did she bleed from the nose, mouth or anus?	radio<br>1 Yes<br>0 No<br>99 Doesn't know<br>88 Refused to answer

	447	[id10243_m]	Did she have noticeable weight loss?	radio<br>1 Yes<br>0 No<br>99 Doesn't know<br>88 Refused to answer
	448	[id10244_m]	Was she severely thin or wasted?	radio<br>1 Yes<br>0 No<br>99 Doesn't know<br>88 Refused to answer
	449	[id10245_m]	During the illness that led to death, did she have a whitish rash inside the mouth or on the tongue?	radio<br>1 Yes<br>0 No<br>99 Doesn't know<br>88 Refused to answer
	450	[id10246_m]	Did she have stiffness of the whole body or was unable to open the mouth?	radio<br>1 Yes<br>0 No<br>99 Doesn't know<br>88 Refused to answer
	451	[id10247_m]	Did she have puffiness of the face?	radio<br>1 Yes<br>0 No<br>99 Doesn't know<br>88 Refused to answer
	452	[id10248_m]<br>Show the field ONLY if:<br>[id10247_m]='1'	For how many days did she have puffiness of the face?	text
	453	[id10249_m]	During the illness that led to death, did she have swollen legs or feet?	radio<br>1 Yes<br>0 No<br>99 Doesn't know<br>88 Refused to answer
	454	[id10250_m]<br>Show the field ONLY if:<br>[id10249_m]='1'	How many days did the swelling last?	text
	455	[id10251_m]<br>Show the field ONLY if:<br>[id10249_m]='1'	Did she have both feet swollen?	radio<br>1 Yes<br>0 No<br>99 Doesn't know<br>88 Refused to answer
	456	[id10252_m]	Did she have general puffiness all over her body?	radio<br>1 Yes<br>0 No<br>99 Doesn't know<br>88 Refused to answer

	457	[id10253_m]	Did she have any lumps?	radio<br>1 Yes<br>0 No<br>99 Doesn't know<br>88 Refused to answer
	458	[id10254_m]<br>Show the field ONLY if: [id10253_m]='1'	Did she have any lumps or lesions in the mouth?	radio<br>1 Yes<br>0 No<br>99 Doesn't know<br>88 Refused to answer
	459	[id10255_m]<br>Show the field ONLY if: [id10253_m]='1'	Did she have any lumps on the neck?	radio<br>1 Yes<br>0 No<br>99 Doesn't know<br>88 Refused to answer
	460	[id10256_m]<br>Show the field ONLY if: [id10253_m]='1'	Did she have any lumps on the armpit?	radio<br>1 Yes<br>0 No<br>99 Doesn't know<br>88 Refused to answer
	461	[id10257_m]<br>Show the field ONLY if: [id10253_m]='1'	Did she have any lumps on the groin?	radio<br>1 Yes<br>0 No<br>99 Doesn't know<br>88 Refused to answer
	462	[id10258_m]	Was she in any way paralysed?	radio<br>1 Yes<br>0 No<br>99 Doesn't know<br>88 Refused to answer
	463	[id10259_m]<br>Show the field ONLY if: [id10258_m]='1'	Did she have paralysis of only one side of the body?	radio<br>1 Yes<br>0 No<br>99 Doesn't know<br>88 Refused to answer
	464	[id10260_m]<br>Show the field ONLY if: [id10258_m]='1'	Which were the limbs or body parts paralysed?	checkbox<br>1 id10260_m___1 Right side<br>2 id10260_m___2 Left side<br>3 id10260_m___3 Lower part of body<br>4 id10260_m___4 Upper part of body<br>5 id10260_m___5 One leg only<br>6 id10260_m___6 One arm only<br>7 id10260_m___7 Whole body<br>8 id10260_m___8 Other
	465	[id10260_check_m]<br>Show the field ONLY if: [id10259_m]='1' and (([id10260_m(1)]='1' and [id10260_m(2)]='1') or [id10260_m(7)]='1')	It is not possible to select "only one side paralysed" and "left and right side" or "whole body" together. Please go back and correct the selection.	descriptive

	466	[id10261_m]	Did she have difficulty swallowing?	radio<br>1 Yes<br>0 No<br>99 Doesn't know<br>88 Refused to answer
	467	[id10262_m]<br>Show the field ONLY if: [id10261_m]='1'	For how many days before death did she have difficulty swallowing?	text
	468	[id10263_m]<br>Show the field ONLY if: [id10261_m]='1'	Was the difficulty with swallowing with solids, liquids, or both?	radio<br>1 Solids<br>2 Liquids<br>3 Both<br>99 Doesn't know<br>88 Refused to answer
	469	[id10264_m]	Did she have pain upon swallowing?	radio<br>1 Yes<br>0 No<br>99 Doesn't know<br>88 Refused to answer
	470	[id10265_m]	Did she have yellow discoloration of the eyes?	radio<br>1 Yes<br>0 No<br>99 Doesn't know<br>88 Refused to answer
	471	[id10266_m]<br>Show the field ONLY if: [id10265_m]='1'	For how many days did she have the yellow discoloration?	text
	472	[id10267_m]	Did her hair change in color to a reddish or yellowish color?	radio<br>1 Yes<br>0 No<br>99 Doesn't know<br>88 Refused to answer
	473	[id10268_m]	Did she look pale (thinning/lack of blood) or have pale palms, eyes or nail beds?<br>*Long term deficiency of the blood results in a pale, whitish appearance of the lips, tongue, and eye sac. Sometimes it is referred to as thinning or lack of blood, or pallor.*	radio<br>1 Yes<br>0 No<br>99 Doesn't know<br>88 Refused to answer
	474	[id10270_m]	Did she drink a lot more water than usual?	radio<br>1 Yes<br>0 No<br>99 Doesn't know<br>88 Refused to answer
	475	[id10486_m]	Did she suffer from extreme fatigue?	radio<br>1 Yes<br>0 No<br>99 Doesn't know<br>88 Refused to answer

	476	[id10485_m]	Did she experience a new loss, change or decreased sense of smell or taste?	radio<br>1 Yes<br>0 No<br>99 Doesn't know<br>88 Refused to answer
	477	[id10294_m]	Section Header: *Signs and Symptoms Associated with Pregnancy and Women*<br>Did she have any swelling or lump in the breast?	radio<br>1 Yes<br>0 No<br>99 Doesn't know<br>88 Refused to answer
	478	[id10295_m]	Did she have any ulcers (pits) in the breast?	radio<br>1 Yes<br>0 No<br>99 Doesn't know<br>88 Refused to answer
	479	[id10296_m]	Did she ever have a period or menstruate?<br>*Ask for period and menstruation, and also mention the content of the subquestions already (excessive quantity or little quantity of bleeding, or irregular vaginal bleeding). If anything is mentioned select yes.*	radio<br>1 Yes<br>0 No<br>99 Doesn't know<br>88 Refused to answer
	480	[id10297_m]<br>Show the field ONLY if: [id10296_m]='1'	When she had her period, did she have vaginal bleeding in between menstrual periods?<br>*Important is the excessive quantity of blood.*	radio<br>1 Yes<br>0 No<br>99 Doesn't know<br>88 Refused to answer
	481	[id10298_m]<br>Show the field ONLY if: [id10297_m]='1'	Was the bleeding excessive?	radio<br>1 Yes<br>0 No<br>99 Doesn't know<br>88 Refused to answer
	482	[id10301_m]<br>Show the field ONLY if: [id10296_m]='1'	Was there excessive vaginal bleeding in the week prior to death?<br>*Important is the excessive quantity of blood.*	radio<br>1 Yes<br>0 No<br>99 Doesn't know<br>88 Refused to answer
	483	[id10299_m]<br>Show the field ONLY if: [id10296_m]='1'	Did her menstrual period stop naturally because of menopause or removal of uterus?	radio<br>1 Yes<br>0 No<br>99 Doesn't know<br>88 Refused to answer
	484	[id10302_m]<br>Show the field ONLY if: [id10299_m]='0' or [id10299_m]='99' or [id10299_m]='88'	At the time of death was her period overdue?	radio<br>1 Yes<br>0 No<br>99 Doesn't know<br>88 Refused to answer
	485	[id10303_m]<br>Show the field ONLY if: [id10302_m]='1'	For how many weeks had her period been overdue?<br>*The answer could be given in another unit, but for data entry use weeks. Less than 1 week=0. 7 days=1 week. Enter "99" for "don't know." Enter "88" for "refuse."*	text

	486	[id10300_m]<br><br>Show the field ONLY if: [id10299_m]='1'	Did she have vaginal bleeding after cessation of menstruation?	radio<br>1 Yes<br>0 No<br>99 Doesn't know<br>88 Refused to answer
	487	[id10304_m]<br><br>Show the field ONLY if: [id10299_m]='0' or [id10299_m]='99' or [id10299_m]='88'	Did she have a sharp pain in her belly (abdomen) shortly before death?	radio<br>1 Yes<br>0 No<br>99 Doesn't know<br>88 Refused to answer
	488	[id10305_m]<br><br>Show the field ONLY if: [id10299_m]='0' or [id10299_m]='99' or [id10299_m]='88'	Was she pregnant or in labour at the time of death?<br>*A "yes" response to this question means a foetus or baby remained in the mother's body after she died, even if she was already in labour.*	radio<br>1 Yes<br>0 No<br>99 Doesn't know<br>88 Refused to answer
	489	[id10306_m]<br><br>Show the field ONLY if: [id10305_m]='0' or [id10305_m]='99' or [id10305_m]='88'	Did she die within 6 weeks of delivery, abortion or miscarriage?<br>*A "yes" response to this question means that a foetus or baby was lost or removed or delivered (vaginally or by C-section) within 6 weeks before she died, whether the baby survived or not.*	radio<br>1 Yes<br>0 No<br>99 Doesn't know<br>88 Refused to answer
	490	[id10307_m]<br><br>Show the field ONLY if: ([id10306_m]='0' or [id10306_m]='99' or [id10306_m]='88') and ([id10305_m]='0' or [id10305_m]='99' or [id10305_m]='88')	Did this woman die more than 6 weeks after being pregnant or delivering a baby?	radio<br>1 Yes<br>0 No<br>99 Doesn't know<br>88 Refused to answer
	491	[id10309_m]<br><br>Show the field ONLY if: [id10305_m]='1' or [id10306_m]='1' or [id10307_m]='1'	For how many months was she pregnant?<br>*Important is the distinction beween early and late pregnancy (threshold is 6 months). For don't know, enter "99." For refused, enter "88."*	text
	492	[id10312_m]<br><br>Show the field ONLY if: [id10305_m]='1'	Section Header: *Questions About Possible Maternal Deaths*<br>Did she die during labour or delivery?<br>*Labor is the period of time by which contractions are less than 10 minutes apart.*	radio<br>1 Yes<br>0 No<br>99 Doesn't know<br>88 Refused to answer
	493	[id10313_m]<br><br>Show the field ONLY if: ([id10312_m]='0' or [id10312_m]='99' or [id10312_m]='88') or [id10306_m]='1' or [id10307_m]='1'	Did she die after delivering a baby?	radio<br>1 Yes<br>0 No<br>99 Doesn't know<br>88 Refused to answer
	494	[id10314_m]<br><br>Show the field ONLY if: [id10313_m]='1' and ([id10306_m]='1' or [id10307_m]='1')	Did she die within 24 hours after delivery?	radio<br>1 Yes<br>0 No<br>99 Doesn't know<br>88 Refused to answer
	495	[id10315_m]<br><br>Show the field ONLY if: [id10314_m]='0' or [id10314_m]='99' or [id10314_m]='88'	Did she die within 6 weeks of childbirth?	radio<br>1 Yes<br>0 No<br>99 Doesn't know<br>88 Refused to answer

	496	[id10316_m]<br>Show the field ONLY if: ([id10312_m]='1' or [id10312_m]='99' or [id10312_m]='88') or [id10313_m]='1'	Did she give birth to a live baby (within 6 weeks of her death)?<br>*The important aspect is if the baby was alive.*	radio<br>1 Yes<br>0 No<br>99 Doesn't know<br>88 Refused to answer
	497	[id10317_m]<br>Show the field ONLY if: [id10305_m]='0' or [id10305_m]='99' or [id10305_m]='88' or [id10312_m]='1' or [id10312_m]='99' or [id10312_m]='88' or ([id10305_m]='1' and [id10312_m]='1')	Did she die during or after a multiple pregnancy?	radio<br>1 Yes<br>0 No<br>99 Doesn't know<br>88 Refused to answer
	498	[id10318_m]<br>Show the field ONLY if: ([id10312_m]='0' or [id10312_m]='99' or [id10312_m]='88') and ([id10314_m]='0' or [id10314_m]='99' or [id10314_m]='88') and [id10316_m]='1'	Was she breastfeeding the child in the days before death?	radio<br>1 Yes<br>0 No<br>99 Doesn't know<br>88 Refused to answer
	499	[id10319_m]<br>Show the field ONLY if: ([id10305_m]='0' or [id10305_m]='99' or [id10305_m]='88' or [id10312_m]='0' or [id10312_m]='99' or [id10312_m]='88') or ([id10305_m]='1' and [id10312_m]='1')	How many births, including stillbirths, did she/the mother have before this baby?<br>*For don't know, enter "99." For refused, enter "88."*	text
	500	[id10320_m]<br>Show the field ONLY if: [id10319_m]>0	Had she had any previous Caesarean section?	radio<br>1 Yes<br>0 No<br>99 Doesn't know<br>88 Refused to answer
	501	[id10321_m]<br>Show the field ONLY if: [id10305_m]='0' or [id10305_m]='99' or [id10305_m]='88' or [id10312_m]='0' or [id10312_m]='99' or [id10312_m]='88' or ([id10305_m]='1' and [id10312_m]='1')	During pregnancy, did she suffer from high blood pressure?	radio<br>1 Yes<br>0 No<br>99 Doesn't know<br>88 Refused to answer
	502	[id10322_m]<br>Show the field ONLY if: [id10305_m]='0' or [id10305_m]='99' or [id10305_m]='88' or [id10312_m]='0' or [id10312_m]='99' or [id10312_m]='88' or ([id10305_m]='1' and [id10312_m]='1')	Did she have foul smelling vaginal discharge during pregnancy or after delivery?	radio<br>1 Yes<br>0 No<br>99 Doesn't know<br>88 Refused to answer
	503	[id10323_m]<br>Show the field ONLY if: [id10305_m]='0' or [id10305_m]='99' or [id10305_m]='88' or [id10312_m]='0' or [id10312_m]='99' or [id10312_m]='88' or ([id10305_m]='1' and [id10312_m]='1')	During the last 3 months of pregnancy, did she suffer from convulsions?	radio<br>1 Yes<br>0 No<br>99 Doesn't know<br>88 Refused to answer

	504	[id10324_m]<br>Show the field ONLY if:<br>[id10305_m]='0' or [id10305_m]='99' or [id10305_m]='88' or [id10312_m]='0' or [id10312_m]='99' or [id10312_m]='88' or ([id10305_m]='1' and [id10312_m]='1')	During the last 3 months of pregnancy did she suffer from blurred vision?	radio<br>1 Yes<br>0 No<br>99 Doesn't know<br>88 Refused to answer
	505	[id10325_m]<br>Show the field ONLY if:<br>[id10305_m]='0' or [id10305_m]='99' or [id10305_m]='88' or [id10312_m]='0' or [id10312_m]='99' or [id10312_m]='88' or ([id10305_m]='1' and [id10312_m]='1')	Did bleeding occur while she was pregnant?	radio<br>1 Yes<br>0 No<br>99 Doesn't know<br>88 Refused to answer
	506	[id10326_m]<br>Show the field ONLY if:<br>[id10325_m]='1'	Was there vaginal bleeding during the first 6 months of pregnancy?	radio<br>1 Yes<br>0 No<br>99 Doesn't know<br>88 Refused to answer
	507	[id10327_m]<br>Show the field ONLY if:<br>[id10325_m]='1'	Was there vaginal bleeding during the last 3 months of pregnancy but before labour started?	radio<br>1 Yes<br>0 No<br>99 Doesn't know<br>88 Refused to answer
	508	[id10328_m]<br>Show the field ONLY if:<br>([id10305_m]='1' and [id10312_m]='1') or [id10306_m]='1' or ([id10305_m]='0' and [id10306_m]='1') or ([id10305_m]='0' and [id10306_m]='0' and [id10307_m]='1') or ([id10316_m]='1' or [id10316_m]='0')	Did she have excessive bleeding during labour or delivery?<br>*Here the excessive quantity of blood DURING birth is what we ask for*	radio<br>1 Yes<br>0 No<br>99 Doesn't know<br>88 Refused to answer
	509	[id10329_m]<br>Show the field ONLY if:<br>([id10305_m]='0' or [id10305_m]='99' or [id10305_m]='88') and ([id10312_m]='1' or [id10312_m]='99' or [id10312_m]='88')	Did she have excessive bleeding after delivery or abortion?<br>*Here the excessive quantity of blood AFTER birth is what we ask for*	radio<br>1 Yes<br>0 No<br>99 Doesn't know<br>88 Refused to answer
	510	[id10330_m]<br>Show the field ONLY if:<br>([id10305_m]='1' and [id10312_m]='1') or [id10306_m]='1' or ([id10305_m]='0' and [id10306_m]='1') or ([id10305_m]='0' and [id10306_m]='0' and [id10307_m]='1') or ([id10316_m]='1' or [id10316_m]='0')	Was the placenta completely delivered?	radio<br>1 Yes<br>0 No<br>99 Doesn't know<br>88 Refused to answer
	511	[id10331_m]<br>Show the field ONLY if:<br>([id10305_m]='1' and [id10312_m]='1') or [id10306_m]='1' or ([id10305_m]='0' and [id10306_m]='1') or ([id10305_m]='0' and [id10306_m]='0' and [id10307_m]='1') or ([id10316_m]='1' or [id10316_m]='0')	Did she deliver or try to deliver an abnormally positioned baby?<br>*Enquire the respondent about theirs understanding of what is an abnormally positioned baby; if unclear or wrong, explain that it refers to babys' whose first body part exiting the vagina is not the head.*	radio<br>1 Yes<br>0 No<br>99 Doesn't know<br>88 Refused to answer

	512	[id10332_m]<br><br>Show the field ONLY if: ([id10305_m]='1' and [id10312_m]='1') or [id10306_m]='1' or ([id10305_m]='0' and [id10306_m]='1') or ([id10305_m]='0' and [id10306_m]='0' and [id10307_m]='1') or ([id10316_m]='1' or [id10316_m]='0')	For how many hours was she in labour?<br>*The answer could be given in another unit, but for data entry use hours. Less than 60 minutes = 0 hours. 1 day=24 hours. Enter "99" for "don't know." Enter "88" for "refuse."*	text
	513	[id10333_m]	Did she attempt to terminate the pregnancy?	radio<br>1 Yes<br>0 No<br>99 Doesn't know<br>88 Refused to answer
	514	[id10334_m]<br><br>Show the field ONLY if: [id10316_m]='0' or [id10316_m]='99' or [id10316_m]='88'	Did she recently have a pregnancy that ended in an abortion (spontaneous or induced)?	radio<br>1 Yes<br>0 No<br>99 Doesn't know<br>88 Refused to answer
	515	[id10335_m]<br><br>Show the field ONLY if: [id10334_m]='1'	Did she die during an abortion?	radio<br>1 Yes<br>0 No<br>99 Doesn't know<br>88 Refused to answer
	516	[id10336_m]<br><br>Show the field ONLY if: [id10334_m]='1' and ([id10335_m]='0' or [id10335_m]='99' or [id10335_m]='88') and ([id10305_m]='0' or [id10305_m]='99' or [id10305_m]='88') and [id10312_m]='1'	Did she die within 6 weeks of having an abortion?	radio<br>1 Yes<br>0 No<br>99 Doesn't know<br>88 Refused to answer
	517	[id10337_m]<br><br>Show the field ONLY if: (([id10312_m]='1' or [id10312_m]='99' or [id10312_m]='88') and [id10305_m]='1') or [id10316_m]='1' or ([id10316_m]='0' and ([id10333_m]='1' or [id10334_m]='1' or [id10336_m]='1')) or ([id10316_m]='0' and [id10333_m]='0' and [id10334_m]='0') or ([id10305_m]='0' and [id10306_m]='0' and [id10307_m]='1')	Where did she give birth / complete the miscarriage / perform the abortion?	radio<br>1 Hospital<br>2 Other health facility<br>3 Home<br>4 On route to hospital or facility<br>5 Other<br>99 Doesn't know<br>88 Refused to answer
	518	[id10338_m]<br><br>Show the field ONLY if: (([id10312_m]='1' or [id10312_m]='99' or [id10312_m]='88') and [id10305_m]='1') or [id10316_m]='1' or ([id10316_m]='0' and ([id10333_m]='1' or [id10334_m]='1' or [id10336_m]='1')) or ([id10316_m]='0' and [id10333_m]='0' and [id10334_m]='0') or ([id10305_m]='0' and [id10306_m]='0' and [id10307_m]='1')	Did she receive professional assistance during the delivery?<br>*Explain to the respondent what is meant by professional assistance: delivery attended by a medical professional (doctor, nurse or midwife).*	radio<br>1 Yes<br>0 No<br>99 Doesn't know<br>88 Refused to answer

	519	[id10339_m]<br><br>Show the field ONLY if:<br>(([id10312_m]='1' or [id10312_m]='99' or [id10312_m]='88') and [id10305_m]='1') or [id10316_m]='1' or ([id10316_m]='0' and ([id10333_m]='1' or [id10334_m]='1' or [id10336_m]='1')) or ([id10316_m]='0' and [id10333_m]='0' and [id10334_m]='0') or ([id10305_m]='0' and [id10306_m]='0' and [id10307_m]='1')	Who delivered the baby / completed the miscarriage?	radio<br>1 Doctor<br>2 Midwife<br>3 Nurse<br>4 Relative<br>5 Self (the mother)<br>6 Traditional birth attendant<br>7 Other<br>99 Doesn't know<br>88 Refused to answer
	520	[id10342_m]<br><br>Show the field ONLY if:<br>[id10313_m]='1' or [id10314_m]='1'	Section Header: *How did the mother deliver her baby?*<br>Was the delivery normal vaginal, without forceps or vacuum?	radio<br>1 Yes<br>0 No<br>99 Doesn't know<br>88 Refused to answer
	521	[id10343_m]<br><br>Show the field ONLY if:<br>[id10342_m]='0' or [id10342_m]='99' or [id10342_m]='88'	Was the delivery vaginal, with forceps or vacuum?	radio<br>1 Yes<br>0 No<br>99 Doesn't know<br>88 Refused to answer
	522	[id10344_m]<br><br>Show the field ONLY if:<br>([id10342_m]='0' or [id10342_m]='99' or [id10342_m]='88') and ([id10343_m]='0' or [id10343_m]='99' or [id10343_m]='88')	Was the delivery a Caesarean section?	radio<br>1 Yes<br>0 No<br>99 Doesn't know<br>88 Refused to answer
	523	[id10347_m]<br><br>Show the field ONLY if:<br>[id10313_m]='1' or [id10314_m]='1'	Was the baby born more than one month early?	radio<br>1 Yes<br>0 No<br>99 Doesn't know<br>88 Refused to answer
	524	[id10340_m]<br><br>Show the field ONLY if:<br>[id10334_m]='1' or [id10315_m]='1' or [id10313_m]='1' or ([id10305_m]='1' and ([id10312_m]='1' or [id10312_m]='0')) or ([id10305_m]='0' and [id10306_m]='0' and [id10307_m]='1')	Did she have an operation to remove her uterus shortly before death?	radio<br>1 Yes<br>0 No<br>99 Doesn't know<br>88 Refused to answer
	525	[id10411_m]	Section Header: *Risk Factors*<br>Did she drink alcohol?	radio<br>1 Yes<br>0 No<br>99 Doesn't know<br>88 Refused to answer
	526	[id10412_m]	Did she use tobacco?	radio<br>1 Yes<br>0 No<br>99 Doesn't know<br>88 Refused to answer

	527	[id10413_m]	Did she smoke tobacco (cigarette, cigar, pipe, etc.)?	radio<br>1 Yes<br>0 No<br>99 Doesn't know<br>88 Refused to answer
	528	[id10414_m]<br>Show the field ONLY if: [id10413_m]='1' or ([id10412_m]='1' and [id10413_m]='0')	What kind of tobacco did she use?	checkbox<br>1 id10414_m___1 Cigarettes<br>2 id10414_m___2 Pipe<br>3 id10414_m___3 Chewing tobacco<br>4 id10414_m___4 Local form of tobacco<br>5 id10414_m___5 Other<br>99 id10414_m___99 Doesn't know<br>88 id10414_m___88 Refused to answer
	529	[id10414_check_m]<br>Show the field ONLY if: [id10413_m]='0' and ([id10414_m(1)]='1' or [id10414_m(2)]='1')	It is not possible to select cigarettes or pipe and "no" to "Did she smoke tobacco?". Please go back and correct the selections.	descriptive
	530	[id10415_m]<br>Show the field ONLY if: [id10414_m(1)] = '1'	How many cigarettes did she smoke daily?	text
	531	[id10416_m]<br>Show the field ONLY if: [id10414_m(2)] = '1' or [id10414_m(3)] = '1' or [id10414_m(4)] = '1' or [id10414_m(5)] = '1'	How many times did she use tobacco products each day?	text
	532	[id10418_m]	Section Header: *Health Service Utilisation*<br>Did she receive any treatment for the illness that led to death?	radio<br>1 Yes<br>0 No<br>99 Doesn't know<br>88 Refused to answer
	533	[id10419_m]<br>Show the field ONLY if: [id10418_m]='1'	Did she receive oral rehydration salts?	radio<br>1 Yes<br>0 No<br>99 Doesn't know<br>88 Refused to answer
	534	[id10420_m]<br>Show the field ONLY if: [id10418_m]='1'	Did she receive (or need) intravenous fluids (drip) treatment?	radio<br>1 Yes<br>0 No<br>99 Doesn't know<br>88 Refused to answer
	535	[id10421_m]<br>Show the field ONLY if: [id10418_m]='1'	Did she receive (or need) a blood transfusion?	radio<br>1 Yes<br>0 No<br>99 Doesn't know<br>88 Refused to answer
	536	[id10422_m]<br>Show the field ONLY if: [id10418_m]='1'	Did she receive (or need) treatment/food through a tube passed through the nose?	radio<br>1 Yes<br>0 No<br>99 Doesn't know<br>88 Refused to answer

	537	[id10423_m]<br><br>Show the field ONLY if:<br>[id10418_m]='1'	Did she receive (or need) injectable antibiotics?	radio<br>1 Yes<br>0 No<br>99 Doesn't know<br>88 Refused to answer
	538	[id10424_m]<br><br>Show the field ONLY if:<br>[id10418_m]='1'	Did she receive (or need) antiretroviral therapy (ART)?	radio<br>1 Yes<br>0 No<br>99 Doesn't know<br>88 Refused to answer
	539	[id10425_m]<br><br>Show the field ONLY if:<br>[id10418_m]='1'	Did she have (or need) an operation for the illness?	radio<br>1 Yes<br>0 No<br>99 Doesn't know<br>88 Refused to answer
	540	[id10426_m]<br><br>Show the field ONLY if:<br>[id10418_m]='1' and [id10425_m]='1'	Did she have the operation within 1 month before death?	radio<br>1 Yes<br>0 No<br>99 Doesn't know<br>88 Refused to answer
	541	[id10427_m]<br><br>Show the field ONLY if:<br>[id10418_m]='1'	Was she discharged from hospital very ill?	radio<br>1 Yes<br>0 No<br>99 Doesn't know<br>88 Refused to answer
	542	[id10432_m]	Was care sought outside the home while she had this illness (that led to death)?	radio<br>1 Yes<br>0 No<br>99 Doesn't know<br>88 Refused to answer
	543	[id10433_m]<br><br>Show the field ONLY if:<br>[id10432_m]='1'	Where or from whom did they seek care?	checkbox<br>1 id10433_m___1 Traditional healer<br>2 id10433_m___2 Homeopath<br>3 id10433_m___3 Religious leader<br>4 id10433_m___4 Government hospital<br>5 id10433_m___5 Government health center or clinic<br>6 id10433_m___6 Private hospital<br>7 id10433_m___7 Community-based practitioner associated with health system<br>8 id10433_m___8 Trained birth attendant<br>9 id10433_m___9 Private physician<br>10 id10433_m___10 Relative, friend (outside household)<br>11 id10433_m___11 Pharmacy<br>99 id10433_m___99 Doesn't know<br>88 id10433_m___88 Refused to answer

	544	[id10433_check_m]<br><br>Show the field ONLY if:<br>([id10433_m(1)]='1' or [id10433_m(2)]='1' or [id10433_m(3)]='1' or [id10433_m(4)]='1' or [id10433_m(5)]='1' or [id10433_m(6)]='1' or [id10433_m(7)]='1' or [id10433_m(8)]='1' or [id10433_m(9)]='1' or [id10433_m(10)]='1' or [id10433_m(11)]='1') and ([id10433_m(99)]='1' or [id10433_m(88)]='1')	It is not possible to select "Don't know" or "Refuse" together with other options. Please go back and correct the selection.	descriptive
	545	[id10435_m]	Did a health care worker tell you the cause of death?	radio<br>1 Yes<br>0 No<br>99 Doesn't know<br>88 Refused to answer
	546	[id10436_m]<br><br>Show the field ONLY if:<br>[id10435_m]='1'	What did the health care worker say?	text
	547	[id10437_m]<br><br>Show the field ONLY if:<br>[id10432_m]='1'	Do you have any health records that belonged to the deceased?	radio<br>1 Yes<br>0 No<br>99 Doesn't know<br>88 Refused to answer<br><br>Field Annotation: @HIDDEN
	548	[id10438_m]<br><br>Show the field ONLY if:<br>[id10437_m]='1'	Can I see the health records?	radio<br>1 Yes<br>0 No<br>99 Doesn't know<br>88 Refused to answer<br><br>Field Annotation: @HIDDEN
	549	[id10439_check_m]<br><br>Show the field ONLY if:<br>[id10438_m]='1'	[Is the date of the most recent (last) visit available?]	radio<br>1 Yes<br>0 No<br><br>Field Annotation: @HIDDEN
	550	[id10439_m]<br><br>Show the field ONLY if:<br>[id10439_check_m]='1'	[Record the date of the most recent (last) visit]	text (date_dmy)<br>Field Annotation: @HIDDEN
	551	[id10440_check_m]<br><br>Show the field ONLY if:<br>[id10438_m]='1'	[Is the date of the second most recent visit available?]	radio<br>1 Yes<br>0 No<br><br>Field Annotation: @HIDDEN
	552	[id10440_m]<br><br>Show the field ONLY if:<br>[id10440_check_m]='1'	[Record the date of the second most recent visit]	text (date_dmy)<br>Field Annotation: @HIDDEN
	553	[id10441_check_m]<br><br>Show the field ONLY if:<br>[id10438_m]='1'	[Is the date of the last note on the health records available?]	radio<br>1 Yes<br>0 No<br><br>Field Annotation: @HIDDEN

	554	[id10441_m]<br><br>Show the field ONLY if:<br>[id10441_check_m]='1'	[Record the date of the last note on the health records]	text (date_dmy)<br>Field Annotation: @HIDDEN
	555	[id10442_m]<br><br>Show the field ONLY if:<br>[id10438_m]='1'	[Record the weight (in kilograms) written at the most recent (last) visit]<br>*(enter e.g. 3.5)*	text<br>Field Annotation: @HIDDEN
	556	[id10443_m]<br><br>Show the field ONLY if:<br>[id10438_m]='1'	[Record the weight (in kilograms) written at the second most recent visit]	text<br>Field Annotation: @HIDDEN
	557	[id10444_m]<br><br>Show the field ONLY if:<br>[id10438_m]='1'	[Transcribe the last note on the health records]	notes<br>Field Annotation: @HIDDEN
	558	[id10450_m]	Section Header: *Background and Context*<br>In the final days before death, did she travel to a hospital or health facility?	radio<br>1 Yes<br>0 No<br>99 Doesn't know<br>88 Refused to answer
	559	[id10451_m]<br><br>Show the field ONLY if:<br>[id10450_m]='1'	Did she use motorised transport to get to the hospital or health facility?	radio<br>1 Yes<br>0 No<br>99 Doesn't know<br>88 Refused to answer
	560	[id10452_m]<br><br>Show the field ONLY if:<br>[id10450_m]='1'	Were there any problems during admission to the hospital or health facility?	radio<br>1 Yes<br>0 No<br>99 Doesn't know<br>88 Refused to answer
	561	[id10453_m]<br><br>Show the field ONLY if:<br>[id10450_m]='1'	Were there any problems with the way she was treated (medical treatment, procedures, interpersonal attitudes, respect, dignity) in the hospital or health facility?	radio<br>1 Yes<br>0 No<br>99 Doesn't know<br>88 Refused to answer
	562	[id10454_m]<br><br>Show the field ONLY if:<br>[id10450_m]='1'	Were there any problems getting medications or diagnostic tests in the hospital or health facility?	radio<br>1 Yes<br>0 No<br>99 Doesn't know<br>88 Refused to answer
	563	[id10455_m]<br><br>Show the field ONLY if:<br>[id10450_m]='1'	Does it take more than 2 hours to get to the nearest hospital or health facility from the deceased's household?	radio<br>1 Yes<br>0 No<br>99 Doesn't know<br>88 Refused to answer
	564	[id10456_m]	In the final days before death, were there any doubts about whether medical care was needed?	radio<br>1 Yes<br>0 No<br>99 Doesn't know<br>88 Refused to answer

	565	[id10457_m]	In the final days before death, was traditional medicine used?	radio<br>1 Yes<br>0 No<br>99 Doesn't know<br>88 Refused to answer
	566	[id10458_m]	In the final days before death, did anyone use a telephone or cell phone to call for help?	radio<br>1 Yes<br>0 No<br>99 Doesn't know<br>88 Refused to answer
	567	[id10459_m]	Over the course of illness, did the total costs of care and treatment prohibit other household payments?	radio<br>1 Yes<br>0 No<br>99 Doesn't know<br>88 Refused to answer
	568	[id10476_m]	Section Header: *Open Narrative*<br>Thank you for your information. Now can you please tell me in your own words about the events that led to the death?	notes
	569	[id10477_m]	[Select any of the following words that were mentioned as present in the narrative.]	checkbox<br>1 id10477_m___1 Chronic kidney disease<br>2 id10477_m___2 Dialysis<br>3 id10477_m___3 Fever<br>4 id10477_m___4 Heart attack<br>5 id10477_m___5 Heart problem<br>6 id10477_m___6 Jaundice<br>7 id10477_m___7 Liver failure<br>8 id10477_m___8 Malaria<br>9 id10477_m___9 Pneumonia<br>10 id10477_m___10 Renal (kidney) failure<br>11 id10477_m___11 Suicide<br>12 id10477_m___12 None of the above words were mentioned<br>99 id10477_m___99 Don't know
	570	[id10477_check_m]<br>Show the field ONLY if: ([id10477_m(12)]='1' or [id10477_m(99)]='1') and ([id10477_m(1)]='1' or [id10477_m(2)]='1' or [id10477_m(3)]='1' or [id10477_m(4)]='1' or [id10477_m(5)]='1' or [id10477_m(6)]='1' or [id10477_m(7)]='1' or [id10477_m(8)]='1' or [id10477_m(9)]='1' or [id10477_m(10)]='1' or [id10477_m(11)]='1')	It is not possible to select "Don't know" or "None of the above" together with other options. Please go back and correct the selection.	descriptive
	571	[verbal_autopsy_mother_complete]	Section Header: *Form Status*<br>Complete?	dropdown<br>0 Incomplete<br>1 Unverified<br>2 Complete

Instrument: **Verbal Autopsy - Newborn** (verbal_autopsy_newborn)

	572	[confirm_studyid_va_n]	Confirm Study ID	text
	573	[date_va_n]	Date of verbal autopsy:	text (date_dmy)

	#	Variable	Field Label	Field Attributes
	574	[id10007]	Section Header: *Information on the respondent and background about interview*<br>What is the sex of VA respondent?	radio<br>1 Female<br>2 Male<br>3 Ambiguous/Intersex
	575	[id10008]	What is your/the respondent's relationship to the deceased?	dropdown<br>1 Parent<br>2 Child<br>3 Other family member<br>4 Friend<br>5 Spouse<br>6 Health worker<br>7 Public official<br>8 Another relationship<br>88 Refused to answer
	576	[id10009]	Did you/the respondent live with the deceased in the period leading to her/his death?	radio<br>1 Yes<br>0 No<br>99 Doesn't know<br>88 Refused to answer
	577	[id10013]	Did the respondent give consent?	radio<br>1 Yes<br>0 No
	578	[ageindays_warning]<br>Show the field ONLY if:<br>([dispbb_neo][current-instance] = '3' or [admitdisposition_neo][current-instance] = '1' or [babealive_swf][current-instance] = '0') and [babedeathdate_swf][current-instance] = ''	Age at death could not be calculated - missing date of death. Please enter date of death on Six-Week Followup form.	descriptive
	579	[ageindays]	Baby's age at death (in days):	calc<br>Calculation: if([dispbb_neo][current-instance] = '3' or [admitdisposition_neo][current-instance] = '1' or [babealive_swf][current-instance] = '0', rounddown(datediff([deldate_del], [babedeathdate_swf][current-instance], 'd')), '')
	580	[displayagechild]<br>Show the field ONLY if:<br>[ageindays]>=28	You will now be filling in the questionnaire for a baby >28 days old.	descriptive
	581	[displayageneonate]<br>Show the field ONLY if:<br>[ageindays]<28	You will now be filling in the questionnaire for a neonate (< 28 days old).	descriptive
	582	[id10487]	In the two weeks before death, did the baby live with or visit someone who had any COVID-19 symptoms or a positive COVID-19 test?	radio<br>1 Yes<br>0 No<br>99 Doesn't know<br>88 Refused to answer
	583	[id10488]	In the two weeks before death, did the baby travel to an area where COVID-19 is known to be present?<br>*Based on self-report of the respondent. If there is doubt, note the location in the narrative and check with the respective supervisor.*	radio<br>1 Yes<br>0 No<br>99 Doesn't know<br>88 Refused to answer

	584	[babeadmit_va]<br><br>Show the field ONLY if: [dispbb_neo][current-instance] != '3' and [admitdisposition_neo][current-instance] != '1'	Section Header: *Health seeking/readmissions*<br><br>Was the newborn admitted for one or more nights at a facility for any reason after being home post-delivery?	yesno<br>1 Yes<br>0 No
	585	[numbabeadmit_va]<br><br>Show the field ONLY if: [babeadmit_va] = '1'	How many times was the baby admitted in the first 6 weeks after birth	dropdown<br>1 1<br>2 2<br>3 3<br>4 >3
	586	[babeadmitage_va]<br><br>Show the field ONLY if: [babeadmit_va] = '1'	How many days old was the baby during the first admission?	text (integer, Min: 1, Max: 42)
	587	[babenightsadm_va]<br><br>Show the field ONLY if: [babeadmit_va] = '1'	For how many nights were they admitted?	text (integer, Min: 1)
	588	[babeadmitpathway_va]<br><br>Show the field ONLY if: [babeadmit_va] = '1'	What was the care-seeking pathway to this admission?	checkbox<br>1 babeadmitpathway_va___1 Routine well-baby visit identified problem<br>2 babeadmitpathway_va___2 Self referral due to parental concern<br>3 babeadmitpathway_va___3 Both self-referral and well-baby visit resulted in care seeking<br>98 babeadmitpathway_va___98 Other
	589	[babeadmitpathwayother_va]<br><br>Show the field ONLY if: [babeadmitpathway_va(98)] = '1'	Other pathway:	text

	590	[babeadmitsymp_va]<br>Show the field ONLY if:<br>[babeadmit_va] = '1'	What were the symptoms the child experienced during this illness (check all that apply)?	checkbox<br>1 babeadmitsymp_va___1 Skin pustules<br>2 babeadmitsymp_va___2 Respiratory distress<br>3 babeadmitsymp_va___3 Watery stool<br>4 babeadmitsymp_va___4 Fever/hotness of body<br>5 babeadmitsymp_va___5 Bilious vomit<br>6 babeadmitsymp_va___6 projectile vomit<br>7 babeadmitsymp_va___7 Not waking up to feed or abnormally sleepy<br>8 babeadmitsymp_va___8 Swelling of both feet<br>9 babeadmitsymp_va___9 Changes in urine color<br>10 babeadmitsymp_va___10 Making less urine than usual<br>11 babeadmitsymp_va___11 Blood in stool<br>12 babeadmitsymp_va___12 Seizure/convulsions<br>13 babeadmitsymp_va___13 Coma<br>14 babeadmitsymp_va___14 Yellow soles<br>15 babeadmitsymp_va___15 Difficulty wtih breastfeeding<br>16 babeadmitsymp_va___16 Cough<br>17 babeadmitsymp_va___17 Umbilical cord problem (pain, discharge, etc.)<br>18 babeadmitsymp_va___18 Not gaining weight<br>98 babeadmitsymp_va___98 Other<br>99 babeadmitsymp_va___99 None
	591	[babeadmitsympother_va]<br>Show the field ONLY if:<br>[babeadmitsymp_va(98)] = '1'	Other symptom:	text
	592	[transfusion_va]<br>Show the field ONLY if:<br>[babeadmit_va] = '1'	Was baby transfused during any admission post-discharge	yesno<br>1 Yes<br>0 No
	593	[babeadmitcond_va]<br>Show the field ONLY if:<br>[babeadmit_va] = '1'	Were you told the child had any of the following conditions?	checkbox<br>1 babeadmitcond_va___1 Sepsis<br>2 babeadmitcond_va___2 Respiratory illness<br>3 babeadmitcond_va___3 Oncologic disease (cancer)<br>4 babeadmitcond_va___4 Congenital abnormality<br>5 babeadmitcond_va___5 Endocrine/ metabolic disease<br>6 babeadmitcond_va___6 Failure to thrive<br>7 babeadmitcond_va___7 Trauma/ injury<br>8 babeadmitcond_va___8 HIV<br>9 babeadmitcond_va___9 Sickle Cell<br>10 babeadmitcond_va___10 Anemia<br>11 babeadmitcond_va___11 Dehydration<br>98 babeadmitcond_va___98 Other<br>99 babeadmitcond_va___99 None

	594	[babeadmitcondother_va]<br>Show the field ONLY if:<br>[babeadmitcond_va(98)] = '1'	Other condition:	text
	595	[matsymp_va]<br>Show the field ONLY if:<br>[babeadmit_va]=1	Was the mother experiencing any signifcant illness at the time when baby was admitted?	yesno<br>1 Yes<br>0 No
	596	[babefeed_va]	Was the baby being exclusively breastfed at the time they died?	yesno<br>1 Yes<br>0 No
	597	[babeseek_va]<br>Show the field ONLY if:<br>[dispbb_neo][current-instance] != '3' and [admitdisposition_neo][current-instance] != '1'	Did you seek care for the newborn at a facility at any time after being home post-delivery that DID NOT result in an admission?	yesno<br>1 Yes<br>0 No
	598	[babeseekage_va]<br>Show the field ONLY if:<br>[babeseek_va] = '1'	How many days old was the baby during the first such visit?	text (integer, Min: 1, Max: 42)
	599	[babeseekpathway_va]<br>Show the field ONLY if:<br>[babeseek_va] = '1'	What was the care-seeking pathway for this visit?	checkbox<br>1 babeseekpathway_va___1 Routine well-baby visit identified problem<br>2 babeseekpathway_va___2 Self referral due to parental concern<br>3 babeseekpathway_va___3 Both self-referral and well-baby visit resulted in care seeking<br>98 babeseekpathway_va___98 Other
	600	[babeseekpathwayother_va]<br>Show the field ONLY if:<br>[babeseekpathway_va(98)] = '1'	Other pathway:	text
	601	[id10077]	Section Header: *History of injuries/accidents*<br>Did the baby suffer from any injury or accident that led to their death?	radio<br>1 Yes<br>0 No<br>99 Doesn't know<br>88 Refused to answer
	602	[id10079]<br>Show the field ONLY if:<br>[id10077]='1'	Section Header: *Injuries and accidents detail*<br>Was it a road traffic accident?	radio<br>1 Yes<br>0 No<br>99 Doesn't know<br>88 Refused to answer
	603	[id10081]<br>Show the field ONLY if:<br>[id10079] = '1'	What was the counterpart that was hit during the road traffic accident?	radio<br>1 Pedestrian<br>2 Stationary object<br>3 Car or light vehicle<br>4 Bus or heavy vehicle<br>5 Motorcycle<br>6 Pedal cycle<br>7 Other<br>99 Doesn't know<br>88 Refused to answer

	604	[id10082]<br>Show the field ONLY if:<br>[id10079] = '0' or [id10079] = '99' or [id10079] = '88'	Was the baby injured in a non-road transport accident?	radio<br>1 Yes<br>0 No<br>99 Doesn't know<br>88 Refused to answer
	605	[id10083]<br>Show the field ONLY if:<br>[id10079] = '0' or [id10079] = '99' or [id10079] = '88'	Was the baby injured in a fall?<br>*This includes accidents and cases where it is unknown if it was an accident or whether there was intentional violence.*	radio<br>1 Yes<br>0 No<br>99 Doesn't know<br>88 Refused to answer
	606	[id10084]<br>Show the field ONLY if:<br>[id10079] = '0' or [id10079] = '99' or [id10079] = '88'	Was there any poisoning?<br>*This includes accidents and cases where it is unknown if it was an accident or whether there was intentional violence.*	radio<br>1 Yes<br>0 No<br>99 Doesn't know<br>88 Refused to answer
	607	[id10085]<br>Show the field ONLY if:<br>[id10079] = '0' or [id10079] = '99' or [id10079] = '88'	Did the baby die of drowning?<br>*This includes accidents and cases where it is unknown if it was an accident or whether there was intentional violence.*	radio<br>1 Yes<br>0 No<br>99 Doesn't know<br>88 Refused to answer
	608	[id10086]<br>Show the field ONLY if:<br>[id10079] = '0' or [id10079] = '99' or [id10079] = '88'	Was the baby injured by a bite or sting by venomous animal?<br>*This includes accidents and cases where it is unknown if it was an accident or whether there was intentional violence.*	radio<br>1 Yes<br>0 No<br>99 Doesn't know<br>88 Refused to answer
	609	[id10087]<br>Show the field ONLY if:<br>[id10086] = '0' or [id10086] = '99' or [id10086] = '88'	Was the baby injured by an animal or insect (non-venomous)?	radio<br>1 Yes<br>0 No<br>99 Doesn't know<br>88 Refused to answer
	610	[id10088]<br>Show the field ONLY if:<br>[id10086] = '1' or [id10087] = '1'	What was the animal/insect?	radio<br>1 Dog<br>2 Snake<br>3 Insect or scorpion<br>4 Other<br>99 Doesn't know<br>88 Refused to answer
	611	[id10089]<br>Show the field ONLY if:<br>[id10079] = '0' or [id10079] = '99' or [id10079] = '88'	Was the baby injured by burns/fire?	radio<br>1 Yes<br>0 No<br>99 Doesn't know<br>88 Refused to answer
	612	[id10090]<br>Show the field ONLY if:<br>[id10079] = '0' or [id10079] = '99' or [id10079] = '88'	Was the baby subject to violence (homicide, abuse)?	radio<br>1 Yes<br>0 No<br>99 Doesn't know<br>88 Refused to answer

	613	[id10092]<br>Show the field ONLY if: [id10079] = '0' or [id10079] = '99' or [id10079] = '88'	Was the baby stabbed, cut or pierced?	radio<br>1 Yes<br>0 No<br>99 Doesn't know<br>88 Refused to answer
	614	[id10093]<br>Show the field ONLY if: [id10079] = '0' or [id10079] = '99' or [id10079] = '88'	Was the baby strangled?	radio<br>1 Yes<br>0 No<br>99 Doesn't know<br>88 Refused to answer
	615	[id10094]<br>Show the field ONLY if: [id10079] = '0' or [id10079] = '99' or [id10079] = '88'	Was the baby injured by a blunt force?	radio<br>1 Yes<br>0 No<br>99 Doesn't know<br>88 Refused to answer
	616	[id10095]<br>Show the field ONLY if: [id10079] = '0' or [id10079] = '99' or [id10079] = '88'	Was the baby injured by a force of nature?	radio<br>1 Yes<br>0 No<br>99 Doesn't know<br>88 Refused to answer
	617	[id10096]<br>Show the field ONLY if: [id10079] = '0' or [id10079] = '99' or [id10079] = '88'	Was it electrocution?	radio<br>1 Yes<br>0 No<br>99 Doesn't know<br>88 Refused to answer
	618	[id10097]<br>Show the field ONLY if: [id10079] = '0' or [id10079] = '99' or [id10079] = '88'	Did the baby encounter any other injury?	radio<br>1 Yes<br>0 No<br>99 Doesn't know<br>88 Refused to answer
	619	[id10098]<br>Show the field ONLY if: [id10079] = '0' or [id10079] = '99' or [id10079] = '88'	Was the injury accidental?	radio<br>1 Yes<br>0 No<br>99 Doesn't know<br>88 Refused to answer
	620	[id10100]<br>Show the field ONLY if: [id10098] = '0' or [id10098] = '99' or [id10098] = '88'	Was the injury intentionally inflicted by someone else?	radio<br>1 Yes<br>0 No<br>99 Doesn't know<br>88 Refused to answer
	621	[id10408]	Section Header: *Health History*<br>Before the illness that led to death, was the baby growing normally?	radio<br>1 Yes<br>0 No<br>99 Doesn't know<br>88 Refused to answer
	622	[id10101]<br>Show the field ONLY if: [ageindays]<28	How many days old was the baby when the fatal illness started?	text

	623	[id10352]<br>Show the field ONLY if: [ageindays]>=28	How old was the child when the fatal illness started? (in days or weeks)	text
	624	[id10120]	Section Header: *Duration of Illness*<br>For how long was the baby ill before death? (in days)	text (integer, Min: 0, Max: 300)
	625	[id10123]	Did the baby die suddenly?<br>*Suddenly means died unexpectedly within 24 hours of being in regular health.*	radio<br>1 Yes<br>0 No<br>99 Doesn't know<br>88 Refused to answer
	626	[id10125]<br>Show the field ONLY if: [ageindays]>=28	Section Header: *Medical History Associated With Final Illness Remind the respondent that we are asking for the diagnosis assessed by a doctor, health worker, or other health professional during the final illness.*<br>Was there any diagnosis by a health professional of tuberculosis?	radio<br>1 Yes<br>0 No<br>99 Doesn't know<br>88 Refused to answer
	627	[id10128]<br>Show the field ONLY if: [ageindays]>=28	Did the baby have a recent positive test by a health professional for malaria?	radio<br>1 Yes<br>0 No<br>99 Doesn't know<br>88 Refused to answer
	628	[id10129]<br>Show the field ONLY if: [ageindays]>=28	Did the baby have a recent negative test by a health professional for malaria?	radio<br>1 Yes<br>0 No<br>99 Doesn't know<br>88 Refused to answer
	629	[id10131]<br>Show the field ONLY if: [ageindays]>=28	Was there any diagnosis by a health professional of measles?	radio<br>1 Yes<br>0 No<br>99 Doesn't know<br>88 Refused to answer
	630	[id10133]<br>Show the field ONLY if: [ageindays]>=28	Was there any diagnosis by a health professional of heart disease?	radio<br>1 Yes<br>0 No<br>99 Doesn't know<br>88 Refused to answer
	631	[id10134]<br>Show the field ONLY if: [ageindays]>=28	Was there any diagnosis by a health professional of diabetes?	radio<br>1 Yes<br>0 No<br>99 Doesn't know<br>88 Refused to answer
	632	[id10135]<br>Show the field ONLY if: [ageindays]>=28	Was there any diagnosis by a health professional of asthma?	radio<br>1 Yes<br>0 No<br>99 Doesn't know<br>88 Refused to answer
	633	[id10136]<br>Show the field ONLY if: [ageindays]>=28	Was there any diagnosis by a health professional of epilepsy?	radio<br>1 Yes<br>0 No<br>99 Doesn't know<br>88 Refused to answer

	634	[id10137]<br>Show the field ONLY if:<br>[ageindays]>=28	Was there any diagnosis by a health professional of cancer?	radio<br>1 Yes<br>0 No<br>99 Doesn't know<br>88 Refused to answer
	635	[id10142]<br>Show the field ONLY if:<br>[ageindays]>=28	Was there any diagnosis by a health professional of sickle cell disease?	radio<br>1 Yes<br>0 No<br>99 Doesn't know<br>88 Refused to answer
	636	[id10143]<br>Show the field ONLY if:<br>[ageindays]>=28	Was there any diagnosis by a health professional of kidney disease?	radio<br>1 Yes<br>0 No<br>99 Doesn't know<br>88 Refused to answer
	637	[id10144]<br>Show the field ONLY if:<br>[ageindays]>=28	Was there any diagnosis by a health professional of liver disease?	radio<br>1 Yes<br>0 No<br>99 Doesn't know<br>88 Refused to answer
	638	[id10147]	Section Header: *General Signs and Symptoms Associated with Final Illness*<br>Did the baby have a fever?	radio<br>1 Yes<br>0 No<br>99 Doesn't know<br>88 Refused to answer
	639	[id10147_measure]<br>Show the field ONLY if:<br>[id10147]='1'	Was the baby's temperature measured using a device?<br>*i.e. thermometer*	yesno<br>1 Yes<br>0 No
	640	[id10147_temp]<br>Show the field ONLY if:<br>[id10147_measure]='1'	What was the baby's temperature? (in Celsius)	yesno<br>1 Yes<br>0 No
	641	[id10148]<br>Show the field ONLY if:<br>[id10147]='1'	How long did the fever last? (in days)	text (number)
	642	[id10149]<br>Show the field ONLY if:<br>[id10147]='1'	Did the fever continue until death?	radio<br>1 Yes<br>0 No<br>99 Doesn't know<br>88 Refused to answer
	643	[id10150]<br>Show the field ONLY if:<br>[id10147]='1' and [ageindays] >=28	How severe was the fever?	radio<br>1 Mild<br>2 Moderate<br>3 Severe<br>99 Doesn't know<br>88 Refused to answer

	644	[id10151]<br>Show the field ONLY if: [id10147]='1' and [ageindays] >=28	What was the pattern of the fever?	radio<br>1 Continuous<br>2 On and off<br>3 Only at night<br>99 Doesn't know<br>88 Refused to answer
	645	[id10152]<br>Show the field ONLY if: [ageindays]>=28	Did the baby have night sweats?	radio<br>1 Yes<br>0 No<br>99 Doesn't know<br>88 Refused to answer
	646	[id10153]	Did the baby have a cough?	radio<br>1 Yes<br>0 No<br>99 Doesn't know<br>88 Refused to answer
	647	[id10154]<br>Show the field ONLY if: [id10153]='1' and [ageindays] >=28	For how long did the baby have a cough? (in days)	text (number)
	648	[id10155]<br>Show the field ONLY if: [id10153]='1' and [ageindays] >=28	Was the cough productive, with sputum?	radio<br>1 Yes<br>0 No<br>99 Doesn't know<br>88 Refused to answer
	649	[id10156]<br>Show the field ONLY if: [id10153]='1' and [ageindays] >=28	Was the cough very severe?	radio<br>1 Yes<br>0 No<br>99 Doesn't know<br>88 Refused to answer
	650	[id10157]<br>Show the field ONLY if: [id10153]='1' and [ageindays] >=28	Did the baby cough up blood?	radio<br>1 Yes<br>0 No<br>99 Doesn't know<br>88 Refused to answer
	651	[id10158]<br>Show the field ONLY if: [id10153]='1' and [ageindays] >=28	Did the baby make a whooping sound when coughing?	radio<br>1 Yes<br>0 No<br>99 Doesn't know<br>88 Refused to answer
	652	[id10159]	Did the baby have any difficulty breathing?	radio<br>1 Yes<br>0 No<br>99 Doesn't know<br>88 Refused to answer
	653	[id10161]<br>Show the field ONLY if: [id10159]='1'	For how many days did the difficulty breathing last?	text (number)

	654	[id10165]<br>Show the field ONLY if: [id10159]='1' and [ageindays] >=28	Was the difficulty continuous or on and off?	radio<br>1 Continuous<br>2 On and off<br>99 Doesn't know<br>88 Refused to answer
	655	[id10166]	During the illness that led to death, did the baby have fast breathing?	radio<br>1 Yes<br>0 No<br>99 Doesn't know<br>88 Refused to answer
	656	[id10167]<br>Show the field ONLY if: [id10166]='1'	For how many days did the fast breathing last? (in days)	text (number)
	657	[id10168]	Did the baby have breathlessness?	radio<br>1 Yes<br>0 No<br>99 Doesn't know<br>88 Refused to answer
	658	[id10169]<br>Show the field ONLY if: [id10168]='1'	For how many days did the baby have breathlessness?	text (number)
	659	[id10172]	Did you see the lower chest wall/ribs being pulled in as the child breathed in?	radio<br>1 Yes<br>0 No<br>99 Doesn't know<br>88 Refused to answer
	660	[id10173]	During the illness that led to death did their breathing sound like any of the following:	checkbox<br>1 id10173___1 Stridor<br>2 id10173___2 Grunting<br>3 id10173___3 Wheezing<br>4 id10173___4 No<br>99 id10173___99 Doesn't know<br>88 id10173___88 Refused to answer
	661	[id10181]	Did the baby have more frequent loose or liquid stools than usual?<br>*In other words, was the stool was "watery" or "water-like"?*	radio<br>1 Yes<br>0 No<br>99 Doesn't know<br>88 Refused to answer
	662	[id10182]<br>Show the field ONLY if: [id10181]='1' and [ageindays] >=28	How long did the baby have frequent loose or liquid stools? (in days)	text (number)
	663	[id10183]<br>Show the field ONLY if: [id10181]='1' and [ageindays]<28	How many stools did the baby have on the day that loose liquid stools were most frequent?	text (number)
	664	[id10184]<br>Show the field ONLY if: [id10181]='1' and [ageindays]<28	How many days before death did the frequent loose or liquid stools start?	text (number)

	665	[id10185]<br>Show the field ONLY if: [id10181]='1' and [ageindays] >=28	Did the frequent loose or liquid stools continue until death?	radio<br>1 Yes<br>0 No<br>99 Doesn't know<br>88 Refused to answer
	666	[id10186]	At any time during the final illness was there blood in the stools?	radio<br>1 Yes<br>0 No<br>99 Doesn't know<br>88 Refused to answer
	667	[id10187]<br>Show the field ONLY if: [id10186]='1' and [ageindays] >=28	Was there blood in the stool up until death?	radio<br>1 Yes<br>0 No<br>99 Doesn't know<br>88 Refused to answer
	668	[id10188]	Did the baby vomit?	radio<br>1 Yes<br>0 No<br>99 Doesn't know<br>88 Refused to answer
	669	[id10194]	To clarify: Did the baby vomit in the week preceding the death?	radio<br>1 Yes<br>0 No<br>99 Doesn't know<br>88 Refused to answer
	670	[id10190]<br>Show the field ONLY if: [id10188]='1' or [id10188]='2' or [id10188]='3'	How long before death did the baby vomit? (days)	text (number)
	671	[id10188_projectile]<br>Show the field ONLY if: [id10188]='1'	Did the baby projectile vomit?<br>*Explain to the respondent that projectile vomiting is when spit-up or vomit forcefully flies out of a baby's mouth. This is different from simple regurgitation.*	radio<br>1 Yes<br>0 No<br>99 Doesn't know<br>88 Refused to answer
	672	[id10191]<br>Show the field ONLY if: [id10188]='1'	Was there blood in the vomit?	radio<br>1 Yes<br>0 No<br>99 Doesn't know<br>88 Refused to answer
	673	[id10188_billious]<br>Show the field ONLY if: [id10188]='1'	Was the vomit green?<br>*Either light or dark green.*	radio<br>1 Yes<br>0 No<br>99 Doesn't know<br>88 Refused to answer
	674	[id10192]<br>Show the field ONLY if: [id10188]='1' and [ageindays] >=28	Was the vomit black?	radio<br>1 Yes<br>0 No<br>99 Doesn't know<br>88 Refused to answer

	675	[id10193]<br>Show the field ONLY if: [ageindays]>=28	Did the baby have any belly (abdominal) problem?<br>*Explain to the respondent that problems could be pain, protruding abdomen or a mass.*	radio<br>1 Yes<br>0 No<br>99 Doesn't know<br>88 Refused to answer
	676	[id10200]<br>Show the field ONLY if: [id10193]='1' and [ageindays] >=28	Did the baby have a more than usually protruding belly (abdomen)?	radio<br>1 Yes<br>0 No<br>99 Doesn't know<br>88 Refused to answer
	677	[id10201]<br>Show the field ONLY if: [id10200]='1'	For how long before death did the baby have a more than usually protruding belly (abdomen)? (in days)	text (number)
	678	[id10203]<br>Show the field ONLY if: [id10200]='1'	How rapidly did the baby develop the protruding belly (abdomen)?	radio<br>1 Rapidly<br>2 Slowly<br>99 Doesn't know<br>88 Refused to answer
	679	[id10204]<br>Show the field ONLY if: [ageindays]>=28	Did the baby have any mass in the belly (abdomen)?	radio<br>1 Yes<br>0 No<br>99 Doesn't know<br>88 Refused to answer
	680	[id10205]<br>Show the field ONLY if: [id10204]='1'	For how long did the baby have a mass in the belly (abdomen)? (in days)	text (number)
	681	[id10214]	Was the baby unconscious during the illness that led to death?	radio<br>1 Yes<br>0 No<br>99 Doesn't know<br>88 Refused to answer
	682	[id10215]<br>Show the field ONLY if: [id10214]='1'	Was the baby unconscious for more than 24 hours before death?	radio<br>1 Yes<br>0 No<br>99 Doesn't know<br>88 Refused to answer
	683	[id10216]<br>Show the field ONLY if: [ageindays]>=28 and [id10214] = '1' and ([id10215] = '0' or [id10215] = '99' or [id10215] = '88')	How many hours before death did the unconsciousness start?	text (number)
	684	[id10217]<br>Show the field ONLY if: [id10214]='1' and [ageindays] >=28	Did the unconsciousness start suddenly, quickly (at least within a single day)?	radio<br>1 Yes<br>0 No<br>99 Doesn't know<br>88 Refused to answer

	685	[id10218]<br><br>Show the field ONLY if: [id10214]='1' and [ageindays] >=28	Did the unconsciousness continue until death?	radio<br>1 Yes<br>0 No<br>99 Doesn't know<br>88 Refused to answer
	686	[id10219]	Did the baby have convulsions?	radio<br>1 Yes<br>0 No<br>99 Doesn't know<br>88 Refused to answer
	687	[id10220]<br><br>Show the field ONLY if: [id10219]='1' and [ageindays] >=28	Did the baby experience any generalized convulsions or fits during the illness that led to death?	radio<br>1 Yes<br>0 No<br>99 Doesn't know<br>88 Refused to answer
	688	[id10221]<br><br>Show the field ONLY if: [id10219]='1' and [ageindays] >=28	For how many minutes did the convulsions last?<br>*The answer could be given in another unit, but for data entry use minutes. Less than 1 minute=0; 1 hour=60 minutes. Enter "99" for "don't know." Enter "88" for "refuse."*	text
	689	[id10222]<br><br>Show the field ONLY if: [id10219]='1' and [ageindays] >=28	Did the baby become unconscious immediately after the convulsion?	radio<br>1 Yes<br>0 No<br>99 Doesn't know<br>88 Refused to answer
	690	[id10223]<br><br>Show the field ONLY if: [ageindays]>=28	Did the baby have any urine problems?<br>*Explain to the respondent that urine problems refer to urinating a lot or not at all, and blood in the urine.*	radio<br>1 Yes<br>0 No<br>99 Doesn't know<br>88 Refused to answer
	691	[id10225]<br><br>Show the field ONLY if: [id10223]='1' and [ageindays] >=28	Did the baby urinate more often than usual?	radio<br>1 Yes<br>0 No<br>99 Doesn't know<br>88 Refused to answer
	692	[id10226]<br><br>Show the field ONLY if: [id10223]='1' and [ageindays] >=28	During the final illness did the baby ever pass blood in the urine?	radio<br>1 Yes<br>0 No<br>99 Doesn't know<br>88 Refused to answer
	693	[id10224]<br><br>Show the field ONLY if: [id10223]='1' and [ageindays] >=28	Did the baby stop urinating?<br>*This means that the deceased stopped urinating.*	radio<br>1 Yes<br>0 No<br>99 Doesn't know<br>88 Refused to answer
	694	[id10227]<br><br>Show the field ONLY if: [ageindays]>=28	Did the baby have sores or ulcers anywhere on the body?	radio<br>1 Yes<br>0 No<br>99 Doesn't know<br>88 Refused to answer

	695	[id10229]<br>Show the field ONLY if: [id10227]='1' and [ageindays] >=28	Did the sores have clear fluid or pus?	radio<br>1 Yes<br>0 No<br>99 Doesn't know<br>88 Refused to answer
	696	[id10230]<br>Show the field ONLY if: [ageindays]>=28	Did the baby have an ulcer (pit) on the foot?	radio<br>1 Yes<br>0 No<br>99 Doesn't know<br>88 Refused to answer
	697	[id10231]<br>Show the field ONLY if: [id10230]='1' and [ageindays] >=28	Did the ulcer on the foot ooze pus?	radio<br>1 Yes<br>0 No<br>99 Doesn't know<br>88 Refused to answer
	698	[id10232]<br>Show the field ONLY if: [id10231]='1' and [ageindays] >=28	For how many days did the ulcer on the foot ooze pus?	text
	699	[id10233]	During the illness that led to death, did the baby have any skin rash?	radio<br>1 Yes<br>0 No<br>99 Doesn't know<br>88 Refused to answer
	700	[id10234]<br>Show the field ONLY if: [id10233]='1' and [ageindays] >=28	For how many days did the baby have the skin rash?<br>*The answer could be given in another unit, but for data entry use days. Less than 1 day or 24 hours = 0 days; 1 week=7 days; 1 month=30 days. Enter "99" for "don't know." Enter "88" for "refuse."*	text
	701	[id10235]<br>Show the field ONLY if: [id10233]='1' and [ageindays] >=28	Where was the rash?	checkbox<br>1 id10235___1 Face<br>2 id10235___2 Trunk or abdomen<br>3 id10235___3 Extremities<br>4 id10235___4 Everywhere
	702	[id10236]<br>Show the field ONLY if: [id10233]='1' and [ageindays] >=28	Did the baby have measles rash (use local term)?	radio<br>1 Yes<br>0 No<br>99 Doesn't know<br>88 Refused to answer
	703	[id10238]<br>Show the field ONLY if: [ageindays]>=28	During the illness that led to death, did their skin flake off in patches?	radio<br>1 Yes<br>0 No<br>99 Doesn't know<br>88 Refused to answer
	704	[id10239]	During the illness that led to death, did the baby have areas of the skin that turned black?	radio<br>1 Yes<br>0 No<br>99 Doesn't know<br>88 Refused to answer

	705	[id10240]	During the illness that led to death, did the baby have areas of the skin with redness and swelling?	radio<br>1 Yes<br>0 No<br>99 Doesn't know<br>88 Refused to answer
	706	[id10241]	During the illness that led to death, did the baby bleed from anywhere?	radio<br>1 Yes<br>0 No<br>99 Doesn't know<br>88 Refused to answer
	707	[id10242]<br>Show the field ONLY if: [id10241]='1'	Did the baby bleed from the nose, mouth or anus (bum)?	radio<br>1 Yes<br>0 No<br>99 Doesn't know<br>88 Refused to answer
	708	[id10243]<br>Show the field ONLY if: [ageindays]>=28	Did the baby have noticeable weight loss?	radio<br>1 Yes<br>0 No<br>99 Doesn't know<br>88 Refused to answer
	709	[id10244]<br>Show the field ONLY if: [ageindays]>=28	Was the baby severely thin or wasted?	radio<br>1 Yes<br>0 No<br>99 Doesn't know<br>88 Refused to answer
	710	[id10245]<br>Show the field ONLY if: [ageindays]>=28	During the illness that led to death, did the baby have a whitish rash inside the mouth or on the tongue?	radio<br>1 Yes<br>0 No<br>99 Doesn't know<br>88 Refused to answer
	711	[id10246]<br>Show the field ONLY if: [ageindays]>=28	Did the baby have stiffness of the whole body or was unable to open the mouth?	radio<br>1 Yes<br>0 No<br>99 Doesn't know<br>88 Refused to answer
	712	[id10247]<br>Show the field ONLY if: [ageindays]>=28	Did the baby have puffiness of the face?	radio<br>1 Yes<br>0 No<br>99 Doesn't know<br>88 Refused to answer
	713	[id10248]<br>Show the field ONLY if: [id10247]='1'	For how many days did the baby have puffiness of the face?	text
	714	[id10249]<br>Show the field ONLY if: [ageindays]>=28	During the illness that led to death, did the baby have swollen legs or feet?	radio<br>1 Yes<br>0 No<br>99 Doesn't know<br>88 Refused to answer

	715	[id10250]<br>Show the field ONLY if: [id10249]='1'	How many days did the swelling last?	text
	716	[id10251]<br>Show the field ONLY if: [id10249]='1'	Did the baby have both feet swollen?	radio<br>1 Yes<br>0 No<br>99 Doesn't know<br>88 Refused to answer
	717	[id10252]<br>Show the field ONLY if: [ageindays]>=28	Did the baby have general puffiness all over their body?	radio<br>1 Yes<br>0 No<br>99 Doesn't know<br>88 Refused to answer
	718	[id10253]<br>Show the field ONLY if: [ageindays]>=28	Did the baby have any lumps?	radio<br>1 Yes<br>0 No<br>99 Doesn't know<br>88 Refused to answer
	719	[id10255]<br>Show the field ONLY if: [id10253]='1'	Did the baby have any lumps on the neck?	radio<br>1 Yes<br>0 No<br>99 Doesn't know<br>88 Refused to answer
	720	[id10256]<br>Show the field ONLY if: [id10253]='1'	Did the baby have any lumps on the armpit?	radio<br>1 Yes<br>0 No<br>99 Doesn't know<br>88 Refused to answer
	721	[id10257]<br>Show the field ONLY if: [id10253]='1'	Did the baby have any lumps on the groin?	radio<br>1 Yes<br>0 No<br>99 Doesn't know<br>88 Refused to answer
	722	[id10258]<br>Show the field ONLY if: [ageindays]>=28	Was the baby in any way paralysed?	radio<br>1 Yes<br>0 No<br>99 Doesn't know<br>88 Refused to answer
	723	[id10259]<br>Show the field ONLY if: [id10258]='1'	Did the baby have paralysis of only one side of the body?	radio<br>1 Yes<br>0 No<br>99 Doesn't know<br>88 Refused to answer

	724	[id10260]<br><br>Show the field ONLY if:<br>[id10258]='1'	Which were the limbs or body parts paralysed?	checkbox<br>1 id10260___1 Right side<br>2 id10260___2 Left side<br>3 id10260___3 Lower part of body<br>4 id10260___4 Upper part of body<br>5 id10260___5 One leg only<br>6 id10260___6 One arm only<br>7 id10260___7 Whole body<br>8 id10260___8 Other
	725	[id10260_check]<br><br>Show the field ONLY if:<br>[id10258]='1' AND (([id10260(1)]='1' AND [id10260(2)]='1') OR [id10260(7)]='1')	It is not possible to select "only one side paralysed" and "left and right side" or "whole body" together. Please go back and correct the selection.	descriptive
	726	[id10261]<br><br>Show the field ONLY if:<br>[ageindays]>=28	Did the baby have difficulty swallowing?<br>*Explain to the respondent that this could mean choking or coughing each time the baby fed.*	radio<br>1 Yes<br>0 No<br>99 Doesn't know<br>88 Refused to answer
	727	[id10262]<br><br>Show the field ONLY if:<br>[id10261]='1'	For how many days before death did the baby have difficulty swallowing?	text
	728	[id10265]	Did the baby have yellow discoloration of the eyes?	radio<br>1 Yes<br>0 No<br>99 Doesn't know<br>88 Refused to answer
	729	[id10266]<br><br>Show the field ONLY if:<br>[id10265]='1' and [ageindays] >=28	For how many days did the baby have the yellow discoloration?	text
	730	[id10268]<br><br>Show the field ONLY if:<br>[ageindays]>=28	Did the baby look pale (thinning/lack of blood) or have pale palms, eyes or nail beds?<br>*Long term deficiency of the blood results in a pale, whitish appearance of the lips, tongue, and eye sac. Sometime it is referred to as thinning or lack of blood, or pallor.*	radio<br>1 Yes<br>0 No<br>99 Doesn't know<br>88 Refused to answer
	731	[id10269]<br><br>Show the field ONLY if:<br>[ageindays]>=28	Did the baby have sunken eyes?	radio<br>1 Yes<br>0 No<br>99 Doesn't know<br>88 Refused to answer
	732	[id10271]	Was the baby able to suckle or bottle-feed within the first 24 hours after birth?	radio<br>1 Yes<br>0 No<br>99 Doesn't know<br>88 Refused to answer
	733	[id10272]	Did the baby ever suckle in a normal way?	radio<br>1 Yes<br>0 No<br>99 Doesn't know<br>88 Refused to answer

	734	[id10273]	Did the baby stop suckling?	radio<br>1 Yes<br>0 No<br>99 Doesn't know<br>88 Refused to answer
	735	[id10274]<br>Show the field ONLY if: [id10273]='1'	How many days after birth did the baby stop suckling?	text
	736	[id10275]	Did the baby have convulsions starting within the first 24 hours of life?	radio<br>1 Yes<br>0 No<br>99 Doesn't know<br>88 Refused to answer
	737	[id10276]<br>Show the field ONLY if: [id10275]='0' or [id10275]='99' or [id10275]='88'	Did the baby have convulsions starting more than 24 hours after birth?	radio<br>1 Yes<br>0 No<br>99 Doesn't know<br>88 Refused to answer
	738	[id10277]	Did the baby's body become stiff, with the back arched backwards?	radio<br>1 Yes<br>0 No<br>99 Doesn't know<br>88 Refused to answer
	739	[id10278]	During the illness that led to death, did the baby have a bulging or raised fontanelle?<br>*Show photo if available.*	radio<br>1 Yes<br>0 No<br>99 Doesn't know<br>88 Refused to answer
	740	[id10279]	During the illness that led to death, did the baby have a sunken fontanelle?<br>*Show photo if available.*	radio<br>1 Yes<br>0 No<br>99 Doesn't know<br>88 Refused to answer
	741	[id10281]	During the illness that led to death, did the baby become unresponsive or unconscious?	radio<br>1 Yes<br>0 No<br>99 Doesn't know<br>88 Refused to answer
	742	[id10282]<br>Show the field ONLY if: [id10281]='1'	Did the baby become unresponsive or unconscious soon after birth, within less than 24 hours?	radio<br>1 Yes<br>0 No<br>99 Doesn't know<br>88 Refused to answer
	743	[id10283]<br>Show the field ONLY if: [id10281]='1' and ([id10282]='0' or [id10282]='99' or [id10282]='88')	Did the baby become unresponsive or unconscious more than 24 hours after birth?	radio<br>1 Yes<br>0 No<br>99 Doesn't know<br>88 Refused to answer

	744	[id10284]	During the illness that led to death, did the baby become cold to touch?	radio<br>1 Yes<br>0 No<br>99 Doesn't know<br>88 Refused to answer
	745	[id10285]<br>Show the field ONLY if: [id10284]='1'	How many days old was the baby when it started feeling cold to touch?<br>*If less than 1 day or 24 hours, enter 0 days. Use 1 week = 7 days to determine the number of days. For don't know, enter "99." For refused, enter "88."*	text
	746	[id10286]	During the illness that led to death, did the baby become lethargic after a period of normal activity?	radio<br>1 Yes<br>0 No<br>99 Doesn't know<br>88 Refused to answer
	747	[id10287]	Did the baby have redness or pus drainage from the umbilical cord stump?	radio<br>1 Yes<br>0 No<br>99 Doesn't know<br>88 Refused to answer
	748	[id10288]	During the illness that led to death, did the baby have skin ulcer(s) or pits?	radio<br>1 Yes<br>0 No<br>99 Doesn't know<br>88 Refused to answer
	749	[id10289]	During the illness that led to death, did the baby have yellow skin, palms (hand) or soles (foot)?	radio<br>1 Yes<br>0 No<br>99 Doesn't know<br>88 Refused to answer
	750	[id10354]	Section Header: *Other Medical History*<br>Was the child part of a multiple birth?<br>*If two or more children are born at the same time, it is counted as a multiple birth, even if one or more of the babies are born dead.*	radio<br>1 Yes<br>0 No<br>99 Doesn't know<br>88 Refused to answer
	751	[id10355]<br>Show the field ONLY if: [id10354]='1'	Was the child the first, second, or later in the birth order?	radio<br>1 First<br>2 Second or later<br>99 Doesn't know<br>88 Refused to answer
	752	[id10435]	Did a health care worker tell you the cause of death?	radio<br>1 Yes<br>0 No<br>99 Doesn't know<br>88 Refused to answer
	753	[id10436]<br>Show the field ONLY if: [id10435]='1'	What did the health care worker say?	text

	754	[id10437]	Do you have any health records that belonged to the deceased?	radio<br>1 Yes<br>0 No<br>99 Doesn't know<br>88 Refused to answer<br><br>Field Annotation: @HIDDEN
	755	[id10438]<br><br>Show the field ONLY if:<br>[id10437]='1'	Can I see the health records?	radio<br>1 Yes<br>0 No<br>99 Doesn't know<br>88 Refused to answer<br><br>Field Annotation: @HIDDEN
	756	[id10439_check]<br><br>Show the field ONLY if:<br>[id10438]='1'	[Is the date of the most recent (last) visit available?]	radio<br>1 Yes<br>0 No<br><br>Field Annotation: @HIDDEN
	757	[id10439]<br><br>Show the field ONLY if:<br>[id10439_check]='1'	[Record the date of the most recent (last) visit]	text (date_dmy)<br>Field Annotation: @HIDDEN
	758	[id10450]	Section Header: *Background and Context*<br><br>In the final days before death, did the mother travel with the baby to a hospital or health facility?	radio<br>1 Yes<br>0 No<br>99 Doesn't know<br>88 Refused to answer
	759	[id10451]<br><br>Show the field ONLY if:<br>[id10450]='1'	Did the baby use motorised transport to get to the hospital or health facility?<br>*For stillbirths and live births delivered in a health facility, answer based on mother's experience.*	radio<br>1 Yes<br>0 No<br>99 Doesn't know<br>88 Refused to answer
	760	[id10452]<br><br>Show the field ONLY if:<br>[id10450]='1'	Were there any problems during admission to the hospital or health facility?<br>*For stillbirths and live births delivered in a health facility, answer based on mother's experience.*	radio<br>1 Yes<br>0 No<br>99 Doesn't know<br>88 Refused to answer
	761	[id10453]<br><br>Show the field ONLY if:<br>[id10450]='1'	Were there any problems with the way the baby was treated (medical treatment, procedures, interpersonal attitudes, respect, dignity) in the hospital or health facility?<br>*For stillbirths and live births delivered in a health facility, answer based on mother's experience.*	radio<br>1 Yes<br>0 No<br>99 Doesn't know<br>88 Refused to answer
	762	[id10454]<br><br>Show the field ONLY if:<br>[id10450]='1'	Were there any problems getting medications or diagnostic tests in the hospital or health facility?<br>*For stillbirths, answer based on mother's experience.*	radio<br>1 Yes<br>0 No<br>99 Doesn't know<br>88 Refused to answer

	763	[id10455]<br>Show the field ONLY if: [id10450]='1'	Does it take more than 2 hours to get to the nearest hospital or health facility from the deceased's household?	radio<br>1 Yes<br>0 No<br>99 Doesn't know<br>88 Refused to answer
	764	[id10456]	In the final days before death, were there any doubts about whether medical care was needed?	radio<br>1 Yes<br>0 No<br>99 Doesn't know<br>88 Refused to answer
	765	[id10457]	In the final days before death, was traditional medicine used?	radio<br>1 Yes<br>0 No<br>99 Doesn't know<br>88 Refused to answer
	766	[id10458]	In the final days before death, did anyone use a telephone or cell phone to call for help?	radio<br>1 Yes<br>0 No<br>99 Doesn't know<br>88 Refused to answer
	767	[id10459]	Over the course of illness, did the total costs of care and treatment prohibit other household payments?	radio<br>1 Yes<br>0 No<br>99 Doesn't know<br>88 Refused to answer
	768	[id10476]	Section Header: *Open Narrative*<br>Thank you for your information. Now can you please tell me in your own words about the events that led to the death?	notes
	769	[id10478]	[Select any of the following words that were mentioned as present in the narrative.]	checkbox<br>1 id10478___1 Asphyxia<br>2 id10478___2 Incubator<br>3 id10478___3 Lung problem<br>4 id10478___4 Pneumonia<br>5 id10478___5 Preterm delivery<br>6 id10478___6 Respiratory distress<br>7 id10478___7 Abdomen<br>8 id10478___8 Cancer<br>9 id10478___9 Dehydration<br>10 id10478___10 Dengue fever<br>11 id10478___11 Diarrhea<br>12 id10478___12 Fever<br>13 id10478___13 Heart problems<br>14 id10478___14 Jaundice (yellow skin or eyes)<br>15 id10478___15 Rash<br>16 id10478___16 None of the above words were mentioned<br>99 id10478___99 Don't know

	770	[id10478_check]<br><br>Show the field ONLY if: ([id10478(1)]='1' or [id10478(2)]='1' or [id10478(3)]='1' or [id10478(4)]='1' or [id10478(5)]='1' or [id10478(6)]='1' or [id10478(7)]='1' or [id10478(8)]='1' or [id10478(9)]='1' or [id10478(10)]='1' or [id10478(11)]='1' or [id10478(12)]='1' or [id10478(13)]='1' or [id10478(14)]='1' or [id10478(15)]='1') and ([id10478(16)]='1' or [id10478(99)]='1')	It is not possible to select "Don't know" or "None of the above" together with other options. Please go back and correct the selection.	descriptive
	771	[verbal_autopsy_newborn_complete]	Section Header: *Form Status*<br><br>Complete?	dropdown<br>0 Incomplete<br>1 Unverified<br>2 Complete
